# Subclonal diversity arises early even in small colorectal tumours and contributes to differential growth fates

**DOI:** 10.1136/gutjnl-2016-312232

**Published:** 2016-09-08

**Authors:** Chelsie K Sievers, Luli S Zou, Perry J Pickhardt, Kristina A Matkowskyj, Dawn M Albrecht, Linda Clipson, Jeffery W Bacher, B Dustin Pooler, Fouad J Moawad, Brooks D Cash, Mark Reichelderfer, Tien N Vo, Michael A Newton, Bret R Larget, Richard B Halberg

**Affiliations:** 1 Department of Oncology, McArdle Laboratory for Cancer Research, University of Wisconsin School of Medicine and Public Health, Madison, Wisconsin, USA; 2 Division of Gastroenterology and Hepatology, Department of Medicine, University of Wisconsin School of Medicine and Public Health, Madison, Wisconsin, USA; 3 Department of Radiology, University of Wisconsin School of Medicine and Public Health, Madison, Wisconsin, USA; 4 Department of Pathology and Laboratory Medicine, University of Wisconsin School of Medicine and Public Health, Madison, Wisconsin, USA; 5 US Department of Veterans Affairs, William S. Middleton Memorial Veterans Hospital, Madison, Wisconsin, USA; 6 Genetic Analysis Group, Promega Corporation, Madison, Wisconsin, USA; 7 Gastroenterology Service, Department of Medicine, Walter Reed National Military Medical Center, Bethesda, Maryland, USA; 8 Gastroenterology Division, Department of Medicine, University of South Alabama, Mobile, Alabama, USA; 9 Department of Statistics, University of Wisconsin–Madison, Madison, Wisconsin, USA; 10 Department of Biostatistics and Medical Informatics, University of Wisconsin–Madison, Madison, Wisconsin, USA; 11 Department of Botany, University of Wisconsin–Madison, Madison, Wisconsin, USA; 12 Carbone Cancer Center, University of Wisconsin–Madison, Madison, Wisconsin, USA

**Keywords:** COLORECTAL CANCER, COLONIC POLYPS, IMAGE ANALYSIS, MOLECULAR GENETICS, STATISTICS

## Abstract

**Objective and design:**

The goal of the study was to determine whether the mutational profile of early colorectal polyps correlated with growth behaviour. The growth of small polyps (6–9 mm) that were first identified during routine screening of patients was monitored over time by interval imaging with CT colonography. Mutations in these lesions with known growth rates were identified by targeted next-generation sequencing. The timing of mutational events was estimated using computer modelling and statistical inference considering several parameters including allele frequency and fitness.

**Results:**

The mutational landscape of small polyps is varied both within individual polyps and among the group as a whole but no single alteration was correlated with growth behaviour. Polyps carried 0–3 pathogenic mutations with the most frequent being in *APC*, *KRAS/NRAS*, *BRAF*, *FBXW7* and *TP53*. In polyps with two or more pathogenic mutations, allele frequencies were often variable, indicating the presence of multiple populations within a single tumour. Based on computer modelling, detectable mutations occurred at a mean polyp size of 30±35 crypts, well before the tumour is of a clinically detectable size.

**Conclusions:**

These data indicate that small colon polyps can have multiple pathogenic mutations in crucial driver genes that arise early in the existence of a tumour. Understanding the molecular pathway of tumourigenesis and clonal evolution in polyps that are at risk for progressing to invasive cancers will allow us to begin to better predict which polyps are more likely to progress into adenocarcinomas and which patients are at greater risk of developing advanced disease.

**Significance of this study**

**What is already known on this subject?**

- Colorectal tumours progress slowly over time from a benign to malignant state through a well-defined adenoma-to-carcinoma sequence.
- Some tumours might be ‘born to be bad’ as not all polyps will progress to invasive cancers.
- Growth rate prior to resection is correlated with tumour stage at resection.
- Vast genetic intratumoral heterogeneity is present in colorectal cancers (CRC), but has not been well documented in adenomas outside of mutations and copy number changes at the *APC* locus.

**What are the new findings?**

- Small polyps can have multiple pathogenic mutations. Traditional adenomas with multiple pathogenic mutations are more likely to be growing, but a specific mutation did not predict the growth fate.
- Pathogenic mutations can be present as private mutations, indicating the presence of one or more subclones.
- Subclonal mutations that are detectable by next-generation sequencing had to arise when the tumour was small.

**How might it impact on clinical practice in the foreseeable future?**

- Understanding the process of clonal evolution in polyps will allow us to better predict which polyps are likely to progress into adenocarcinomas and which patients are predisposed to developing invasive cancers while simultaneously decreasing the burden of CRC screening and decreasing the incidence of metastatic CRC.

## Background

**Figure 1:**
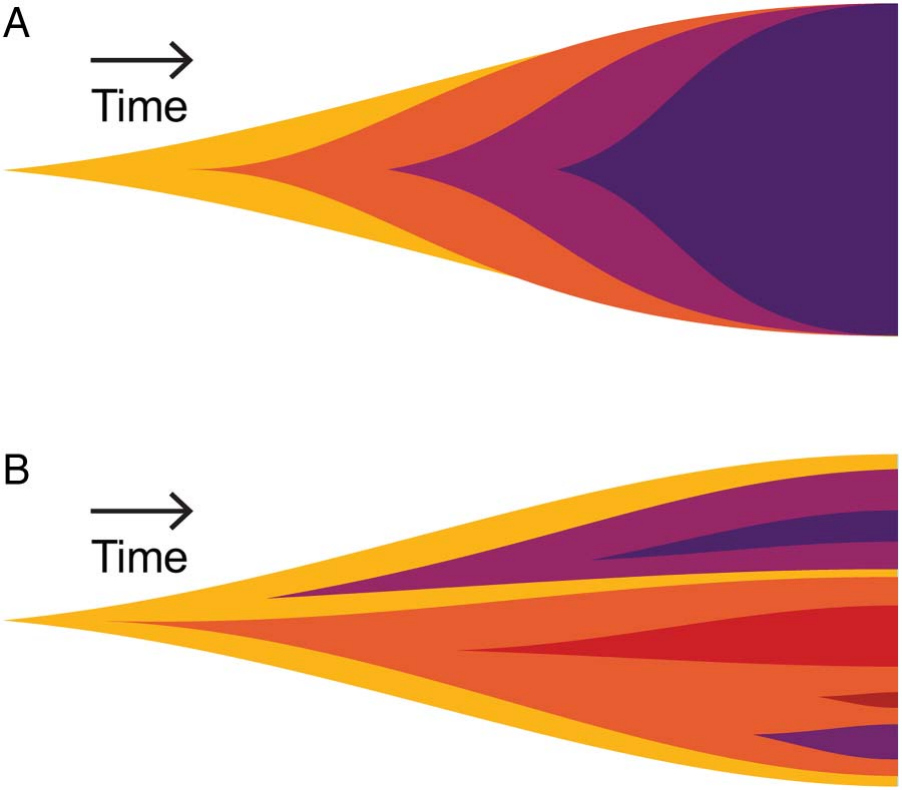
Different models of tumour evolution have been proposed. (A) In the stepwise accumulation of mutations model, sequential acquisition of mutations drives the fittest clone towards a metastatic phenotype. (B) In the Big Bang model, many mutations happen early during tumourigenesis. Major subclones are expanded and maintained over time. Additional mutations can be acquired as late events, but the population of cells carrying these late mutations do not reach a significant proportion of the tumour.

For years, cancer biologists have accepted that colorectal cancers slowly progress over time from a benign to malignant state through a well-defined adenoma-to-carcinoma sequence in which molecular changes have been linked to specific pathological states.^1^ This theory was based on the observation of many tumours in various stages of disease across many different individuals. However, we now know that not all adenomas will progress to invasive adenocarcinomas: some remain static in size and some ultimately regress and completely resolve.^2^ ^3^ The accumulation of pathogenic mutations has been thought to drive tumour progression with each new mutation conferring the transition to the next pathological state in the adenoma-to-carcinoma sequence. The resulting tumour grows as the subclone with the most advantageous mutations outcompetes less fit clones in a Darwinian fashion (figure 1A). The tumour continues to grow until a more deleterious combination of mutations is acquired and consequently the tumour invades and spreads.

In 80%–90% of colorectal tumours, tumourigenesis appears to be initiated following the loss of activity of the tumour suppressor gene *APC* via two inactivating mutations or one mutation followed by a loss-of-heterozygosity event.^4^ A member of the β-catenin destruction complex, loss of functional APC results in aberrant nuclear localisation of β-catenin, and dysregulated WNT signalling. Mutations in *APC* are then followed by mutations in *KRAS/NRAS,* the TGFβ pathway, *PIK3CA* or *TP53* or any combination of several of these events.^5^ This stepwise accumulation of mutations has been thought to be responsible for driving traditional colon polyps to advanced cancers.

With the emergence of next-generation sequencing, investigators are finding overwhelming genetic heterogeneity across many cancer types. Multiple populations can be resolved comparing allelic frequencies^6^ ^7^ or spatial distribution.^8^ ^9^ Even phenotypically normal tissue has been found to have pathogenic mutations that do not confer a visible phenotype.^10–13^ These observations indicate that the slow stepwise accumulation of mutations driving adenoma progression might instead be a rapid acquisition of mutations during the earliest cell divisions during tumourigenesis in some tumours (figure 1B).^14^ ^15^ In this study, we profiled the genetic landscape of 48 initially small (<9 mm) colorectal polyps with known growth fates^2^ and sought to more fully understand the relationship between the number of driver mutations, the timing of mutation acquisition and polyp growth.

## Methods

### Cohort selection and DNA isolation

All human studies were performed under the Institutional Review Board approval at the University of Wisconsin. Pickhardt *et al*^2^ serially monitored 306 polyps ranging in starting size from 6 to 9 mm by CT colonography. Per cent volumetric grow rate per year was determined as previously described.^2^ Of the 306 with known growth fates, 48 resected polyps were selected based on amount of remaining formalin-fixed paraffin-embedded (FFPE) tissue and from a variety of growth fates. These selected tumours were removed from 36 asymptomatic patients identified at normal colorectal cancer screening from the University of Wisconsin Hospital and Clinics as well as the Walter Reed National Military Medical Center. These individuals had a mean age of 57±8 years and 28 (78%) were male. DNA was isolated from FFPE tissue scraped from 5 µm sections using the Maxwell DNA FFPE Kit (Promega, Madison, Wisconsin, USA) and eluted into a volume of 30 µL of buffer following the manufacturer's instructions.

### Targeted sequencing and variant calling

Isolated genomic DNA was submitted to the University of Wisconsin-Madison Biotechnology Center. DNA concentration was verified using the Qubit dsDNA HS Assay Kit (Life Technologies, Carlsbad, California, USA). Samples were prepared as described in the Ion AmpliSeq Library Preparation User Guide, Publication #MAN0006735 Rev. A.0 (Life Technologies) using the Ion AmpliSeq Library Kit V.2.0 with Ion AmpliSeq Cancer Hotspot Panel V.2 (Life Technologies). This targeted sequencing panel covers 50 cancer-related genes and is similar to the sequencing panels used in the clinic. Ion Xpress Barcode Adapters 1-16 Kit (Life Technologies) was used during the adapter ligation step of the library preparation to uniquely barcode each sample. Following option 3 of the user guide, libraries were amplified prior to a quality and quantity check. Initial quantity was assessed with the Qubit dsDNA HS Assay Kit. Quantity and quality were further assessed with an Agilent High Sensitivity DNA Kit. Libraries were diluted to 100 pM based on molarity values from the Agilent High Sensitivity DNA Assay prior to pooling. An equimolar mix of barcoded libraries was prepared and then diluted to 8 pM. The 8 pM library pool was used in preparation of template-positive Ion Sphere Particles (ISPs) containing clonally amplified DNA using the Ion PGM Template OT2 200 Kit on the Ion OneTouch 2 System (Life Technologies). Template-positive ISPs were enriched using the Ion OneTouch ES all as described in the Ion PGM Template OT2 200 Kit User Guide, Publication #MAN0007220 Rev. 5.0 (Life Technologies). Enriched ISPs were loaded onto an Ion 318 Chip V.2 and sequenced with the Ion PGM Sequencing 200 Kit v2 on an Ion PGM System as described in the Ion PGM Sequencing 200 Kit V.2 User Guide, Publication #MAN0007273 Rev. 3.0 (Life Technologies).

Data analysis was performed using the Torrent Suite Software V.4.0.2 (Life Technologies) for samples 1–26 and V.4.4.2 for samples 239–298. CHP2.20131001 was used for the target and hotspot regions. The variant calling was done with Somatic-PGM using low stringency settings. Variants in samples 1–26 were eliminated due to likelihood of false positives with FFPE samples if the allelic frequency was <5%, the quality score was <10 and there were <10 reads, strand bias or known mispriming events.^16^ Similarly, variants in samples 239–298 were eliminated if they had allelic frequencies <5%, a quality score <30 or had <10 reads as well as all known mispriming events. Differences in quality control were used based on collection site, University of Wisconsin Hospital and Clinics or Walter Reed National Military Medical Center, owing to differences in the concentration and quality of FFPE DNA. All polyps were evaluated for tumour cellularity, a measure of the percentage of tumour cells present in the sample, by a board-certified clinical pathologist. Cellularity was used to calculate adjusted allelic frequencies (see online supplementary files S1 and S2). Variant annotation was performed using Ensemble Variant Effect Predictor^17^ and cross-referenced with the Catalogue of Somatic Mutations in Cancer^18^ and the International Agency for Research on Cancer TP53 Database.^19^ Only mutations that are known to have an adverse effect on the resulting protein are listed in this study. These are described as ‘pathogenic mutations’. Variants of unknown significance, regardless of their allelic frequency, are not represented in these data owing to a lack of non-tumour tissue controls. Mutations were described as public, indicating that they are likely present in every neoplastic cell and are thus clonal, if the adjusted allelic frequency was >30%. Mutations were classified as private if they fell above the quality control thresholds but were present at adjusted allelic frequencies <30%, indicating that they are subclonal.

10.1136/gutjnl-2016-312232.supp2 supplementary file

10.1136/gutjnl-2016-312232.supp3 supplementary file

### Low-frequency variant mutation validation

Low-frequency variants for which commercial primers were available were validated with TaqMan Mutation Detection Assays (Thermo Fisher) (see online supplementary figure S1). Mutation detection was performed according to assay instructions. Briefly, FFPE tissue was microdissected from multiple regions of each tumour (see online supplementary figure S1A–C) under a dissection microscope and DNA was purified using the Maxwell DNA FFPE Kit (Promega). Samples were run in duplicate or triplicate as per manufacturer's instructions to determine the presence of or the frequency of the variant DNA, respectively. The qPCR reactions were run on Bio-Rad CFX96 Real-Time PCR and data were analysed using the Mutation Detector software (Thermo Fisher, last revised April 2012). Variants that fell below our variant calling cut-offs for sequencing, but that were validated by qPCR were included in the dataset. This included the *KRAS* variant for polyp PF24 and the removal of the *CTNNB1* variant in PF11.

10.1136/gutjnl-2016-312232.supp1 supplementary figures and tables

### Microsatellite instability testing

DNA that remained after sequencing, prepared as noted above, underwent microsatellite instability (MSI) testing as previously described.^20^ Without matching normal tissue samples, tumour only samples were classified as MSI-High (MSI-H) that had three or more alleles per marker, as this is a rare event in normal cells, in at least two of the five markers in the panel.

### Computational framework and statistical modelling

We adapted a previously described statistical inference framework^14^ to accommodate targeted sequencing mutation data that is acquired from a representative slice of the whole tumour. This framework uses Approximate Bayesian Computation (ABC) to estimate the distribution of the size of the tumour at which mutations occur by comparing a three-dimensional model of tumour growth to the targeted next-generation sequencing data obtained from our cohort of human colon polyps. This method models tumour growth by crypt fission.^21^ ^22^ Given that colon tumours maintain their glandular structures and that private mutations from bulk sequencing are present on a clonal or public level when individual crypts are sequenced,^15^ this is an appropriate method of modelling colon tumour growth to a realistic size on a computationally manageable scale. This framework allows for input of mutation rate, the tracking of variable fitness changes after mutation occurrence, size when novel mutations arose as well as the mutation profile of every crypt in the matrix.

ABC inference involves the repeated forward simulation of synthetic tumours followed by selection of those in silico tumours that have mutation profiles matching the observed tumour profiles. Single crypts are seeded into a three-dimensional matrix with each sequence beginning with the random choice of a crypt in the matrix. The crypt has a chance to die and be removed from the matrix with a probability=0.2/fitness. Fitness, in the absence of novel mutations, is set to 1 with the default probability of death=20%. If the crypt survives, it then undergoes fission and a daughter crypt is generated. Fission can happen to any crypt in the tumour mass; it is not restricted to the periphery. Each new daughter crypt either fills an empty adjacent space to the parent crypt or displaces the crypt occupying an adjacent space, pushing existing crypts outwards. The daughter crypt can acquire *n* new mutations drawn at random from the Poisson probability distribution:
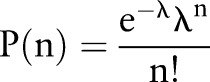


where λ represents the probability of n independent mutations occurring (λ=5×10^−4^ and 5×10^−2^ were used in this study). If no new mutation is acquired, the daughter crypt maintains the mutation profile of its parent. If a new mutation is acquired, a change in fitness is drawn from a normal distribution (µ=0, σ=0.2). This allows for negative, neutral and positive fitness changes. This sequence is repeated until the desired size is reached. A final size of 333 333 crypts was used here, which corresponds to a 10 mm^3^ tumour mass given each crypt contains 3000 cells.

Once the final size is reached, each generated tumour is sectioned and the mutation profile is sampled. Crypts with an x coordinate of zero are taken as a representative of a slice down the middle of the tumour. Mutant allele frequencies are calculated by dividing the total number of crypts in the slice containing each mutation by the total number of crypts captured in the slice. Tumours with mutant allele frequencies ≥10% were accepted as matching the observed sequencing data from our cohort of polyps. This cut-off was used to represent the lower limits of detection of targeted sequencing. While mutations with allelic frequencies between 5% and 10% can be reliably detected from targeted next-generation sequencing, we used a 10% cut-off for our model. Given the context of monoallelic mutations, of which the model does not account for, this 10% cut-off would be equivalent to 5%.

While other models have addressed local spread, the effects of spatial constraints and differences in tumour microenvironment,^23^ these parameters were not specifically modelled in this study. C++ code for the three-dimensional tumour growth model as well as the sampling and reading of mutation profiles are available, see online supplementary files S3–S5.

10.1136/gutjnl-2016-312232.supp4 supplementary file

10.1136/gutjnl-2016-312232.supp5 supplementary file

10.1136/gutjnl-2016-312232.supp6 supplementary file

## Results

### Natural history of small polyps in humans

**Figure 2:**
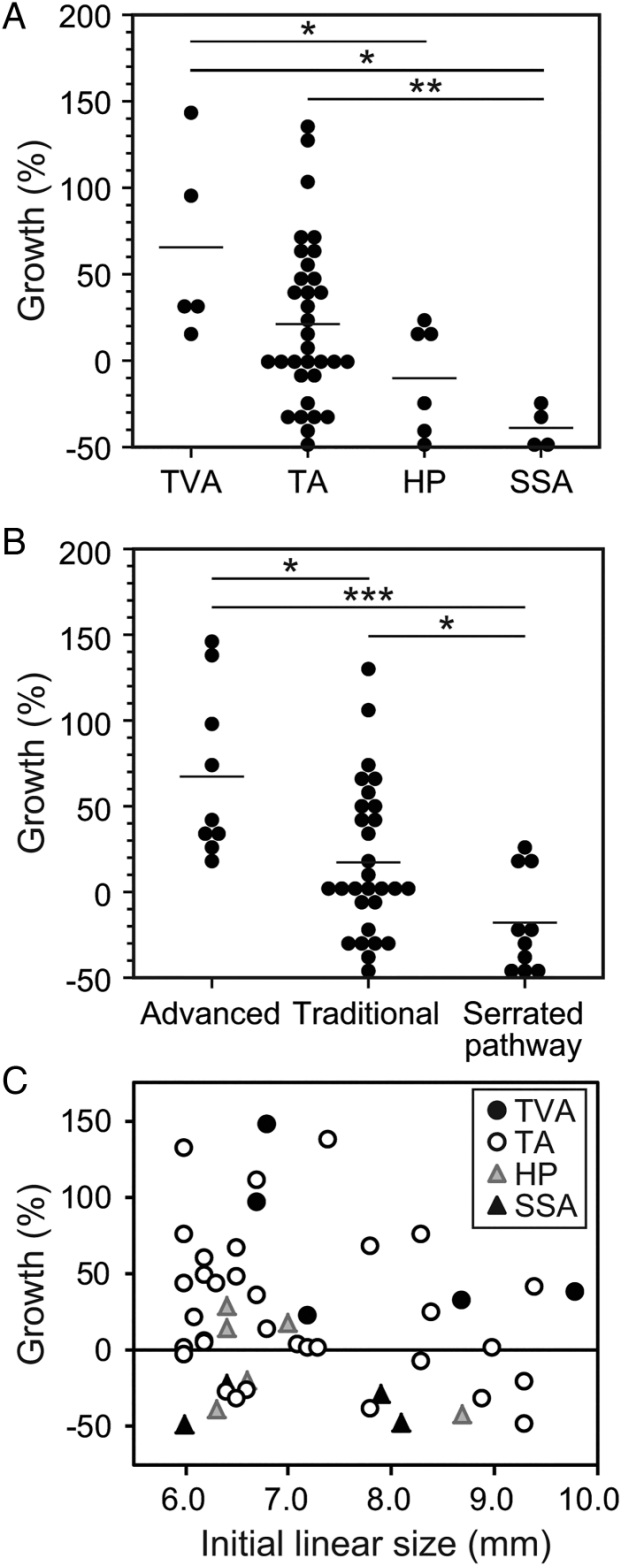
Pathology correlates with per cent volumetric growth. (A) Per cent volumetric growth rates are shown for individual polyps classified as tubulovillous adenomas (TVAs), tubular adenomas (TAs), hyperplastic polyps (HPs) or sessile serrated adenomas (SSAs). Horizontal lines represent the means (p value=0.006, Kruskal-Wallis test). Pairwise comparisons based on the Wilcoxon rank-sum test are shown as *p≤0.05, **p≤0.01, ***p≤0.001. (B) Per cent volumetric growth rates for individual polyps are classified as advanced adenomas (≥10 mm in linear size, villous component or high-grade dysplasia), traditional adenomas or serrated pathway polyps (HPs and SSAs) are shown. Horizontal lines represent the mean (p value=0.0008, Kruskal-Wallis test) with pairwise p values as represented in (A). Advanced adenomas differ from traditional adenomas (p value=0.013, Wilcoxon rank-sum test). (C) Initial linear size is not correlated with per cent volumetric growth (R^2^=0.038, p value=0.265, Kendall's rank correlation). Note the 48 resected polyps shown here were selected from the original Pickhardt *et al* study.^2^

Small polyps in humans were followed at 1–3 year intervals for 1–6 years (mean 1.6 ±1.0 years) by CT colonography as previously described.^2^ Polyps were assigned to one of three growth fate categories: 46% (22/48) were classified as growing (>20% volumetric growth per year), 25% (12/48) were classified as static (20% to −20% volumetric growth per year) and 29% (14/48) were classified as regressing (<−20% volumetric growth per year). Additionally, polyps had different pathologies: 69% (33/48) were classified as tubular adenomas (TAs), one of which had high-grade dysplasia (2%, 1/48); 10% (5/48) were tubulovillous adenomas (TVAs); 8% (4/48) were sessile serrated adenomas (SSAs); and 13% (6/48) were hyperplastic polyps (HPs) (figure 2A). Pathology correlated with growth fate (p value=0.006, Kruskal-Wallis test) although this relationship was primarily due to the difference between TVAs, HPs and SSAs. TVAs, TAs, HPs and SSAs had mean growth rates of 66.8%, 24.2%, −7.2% and −37.6%, respectively. While all four SSAs were regressing and this was statistically significant, we recognise that this small subset is unlikely to represent all SSAs and their possible fates.

Advanced adenomas are considered the clinically relevant step between small adenomas and stage 1 adenocarcinomas and are the primary target of screening colonoscopy and polypectomy. Advanced adenomas in this setting are classified as TAs with a villous component, high-grade dysplasia, >10 mm in maximum diameter or sessile serrated polyps/SSAs with cytological dysplasia. At resection, nine of the traditional polyps (TAs and TVAs) in this study were classified as advanced adenomas based on the above criteria. Advanced adenomas had a mean growth rate of 68%, compared with 18% for all traditional adenomas; these classifications were significantly correlated with growth (p value=0.013, Wilcoxon rank-sum test) (figure 2B). Despite the inclusion of final maximum diameter (>10 mm) in advanced adenoma classification and the correlation of advanced adenoma classification and growth, initial linear size was not correlated with volumetric polyp growth (figure 2C) (R^2^=0.038, p value=0.265, Kendall's rank correlation). Additionally, polyp location was not associated with growth (p value=0.141, Kruskal-Wallis test) (see online supplementary files S1 and S2).

### Small polyps can have multiple pathogenic mutations

**Figure 3:**
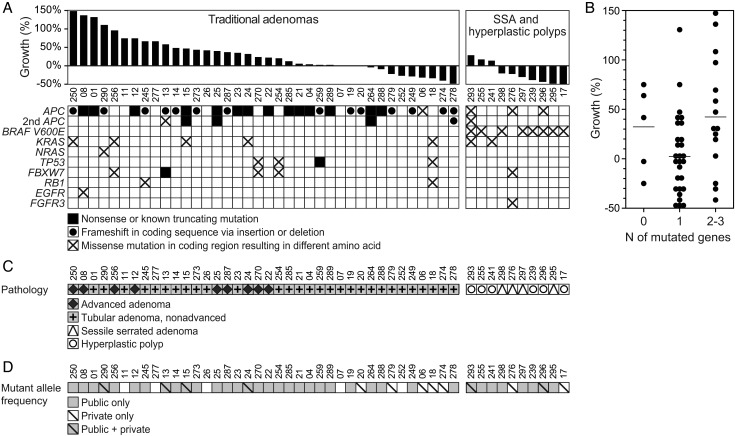
Small polyps often carried multiple pathogenic mutations. (A) Mutation profile of polyps with known growth fates is shown. Only well-annotated, known pathogenic variants are included. (B) Small polyps had 0–3 pathogenic mutations. Horizontal lines represent the mean (p value=0.044, Kruskal-Wallis test). The difference between polyps with one mutation and those with two or more was significant (p value=0.020, Wilcoxon rank-sum test). (C) The pathology of polyps with known growth fates (A) compared with mutation frequency. (D) The mutations can be classified as public, that is, clonal with an adjusted allele frequency of ≥30% or private, that is, subclonal with an adjusted allele frequency of 5%–30%. Small polyps with only private mutation(s) tended to regress. Private only versus public only and public and private were significantly different (p values=0.002 and 0.032, respectively, Wilcoxon rank-sum test).

Similar to their malignant counterparts, small (6–9 mm) colorectal polyps harbour mutations in traditional driver genes including *APC, KRAS, TP53* and *BRAF* (figure 3A, see online supplementary table S1, and supplementary file S1). Sixty-seven per cent (32/48) of all polyps, regardless of pathology, had mutations in the *APC* gene. Eight per cent (4/48) had codon 12 or 13 mutations in *KRAS,* with 15% (7/48) having any mutation in *KRAS*. One polyp carried a codon 12 mutation in *NRAS* (p.N12C). Eight per cent (4/48) polyps carried mutations in *TP53*. Ten per cent (5/48) had mutations in *FBXW7*, similar to the incidence in colorectal cancers.^24^ Seventeen per cent (8/48) of all polyps carried the *BRAF p.V600E* mutation; however, this was restricted to SSAs and HPs and found in the majority 80% (8/10) of these polyps.

No genetic features were unique to, nor consistent across, the nine advanced adenomas (figure 3). Advanced adenomas had one to two known pathogenic mutations, the most common (7/9) being truncating or frameshift mutations in *APC*. Three out of nine advanced adenomas had *KRAS* mutations in codon 12, 13 or 61; however, *KRAS* mutations were not exclusive to advanced adenomas (figure 3). Furthermore, *KRAS* mutations were not exclusive to growing polyps; 2/48 samples had mutations in *KRAS* yet remained static in size or regressed in size.

Contrary to the stepwise accumulation of mutations, small colon polyps can have multiple pathogenic mutations in crucial driver genes (figure 3 and see online supplementary file S1). Six per cent (3/48) had mutations in three different driver genes, 25% (12/48) had mutations in two different driver genes, 58% (28/48) had mutations in only one driver gene and 10% (5/48) had zero pathogenic mutations detected. Pathogenic mutation burden was correlated with polyp growth across all histological subtypes (p value=0.044, Kruskal-Wallis test) and this observed difference accounted for the difference between two or more pathogenic mutations and those with one (p value=0.02, Wilcoxon rank-sum test) (figure 3B). There was no difference between those that had zero detected pathogenic mutations and either one or two to three detected (p value=0.16 and 0.76, respectively, Wilcoxon rank-sum test). When data were divided into traditional versus serrated pathways, this trend held true for the traditional adenomas (figure 3C) with two to three pathogenic mutations having a mean growth rate of 60% compared with those with one pathogenic mutation, which had a mean growth rate of 13% (p value=0.01, Wilcoxon rank-sum test). This trend, however, did not hold true for the serrated pathway polyps (p value=0.667, Wilcoxon rank-sum test).

While the mechanism of clinical regression is unknown, we hypothesised that early loss of mismatch repair (MMR) activity could create more immunogenic polyps. No polyps carried mutations in the MMR gene *hMLH1*; however, deficient MMR in the setting of non-familial cases of colorectal cancer primarily occurs via methylation of *hMLH1*.^25^ ^26^ Deficiency of the MMR genes is manifested in changes in the length of microsatellite repeat sequences, which can be detected in colon polyps by using a novel panel of long mononucleotide repeats.^20^ Only two polyps displayed MSI: PF18 (regressing) and PF19 (stable); therefore, no relationship between MSI status and growth could be determined (see online supplementary table S3). Additional samples would be needed to determine if a relationship between polyp regression and MSI status exists.

### Adenomas contain subpopulations at lower allelic frequencies

Even small adenomas can have subpopulations containing unique pathogenic variants (figure 3D, see online supplementary table S1). Variants were classified as public mutations, common to every tumour cell, or as private mutations, present in only a subset of tumour cells. Public mutations included variants with adjusted allelic frequencies ranging from 70% to 100%, indicating the variant was present in both alleles or in one allele in combination with a loss-of-heterozygosity event, or at approximately 50% (range 30%–70%) indicating that the variant was present in only one allele. Polyps fell into four categories: those with no detectable mutations, public mutations only, private mutations only and those with both public and private mutations (figure 3D). The polyps which only contained private mutations at low allelic frequencies (<30%) had variants in driver mutations that were classified as public mutations in other polyps, including frameshift and truncating mutations in *APC* and the *BRAF p.V600E* variant. Tumours with only public mutations and those that contained both public and private mutations did not have statistically different per cent volumetric growth rates, 26±52% and 39±50%, respectively (p value=0.329, Wilcoxon rank-sum test). Hypothesising that a more malignant subclone could be responsible for overall tumour growth, and given that polyps that contained two or more pathogenic mutations were more likely to be growing, the clonality of these polyps was compared. Polyps which harboured at least one public and one private mutation were not more likely to be growing when compared with polyps which had two or more public only mutations (p value=0.568, Wilcoxon rank-sum test). Furthermore, the growth rates of polyps which harboured at least one public and one private mutation were not statistically different from those polyps which contained only a single public mutation (p value=0.132, Wilcoxon rank-sum test), while polyps that contained multiple public mutations were more likely to be growing when compared with those with only a single public mutation (p value=0.015, Wilcoxon rank-sum test). None of the clonality groups were associated with advanced adenoma classification. Interestingly, tumours in which only private mutations were detected had a significantly lower mean growth rate of −28±16% (Kruskal-Wallis test, p value=0.006).

### Statistical inference predicts that detectable intratumoral heterogeneity arises early

**Figure 4:**
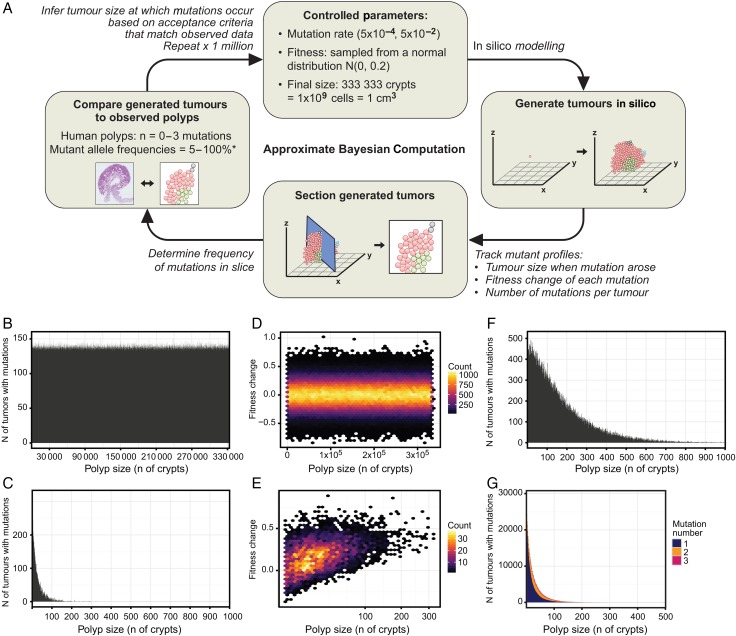
Statistical inference predicts that detectable mutations arise early. (A) The Approximate Bayesian Computation (ABC) framework is shown. (B) Prior distribution of all mutations acquired by in silico tumours demonstrates that mutations can occur at any size in the model. (C) Posterior distribution of mutations that fit acceptance criteria (≥10% mutant allele frequency) and the size of the tumour when that mutation arose are shown. (D) Prior distribution of the fitness change of all mutations acquired by in silico tumours demonstrates that fitness can be positive, neutral or negative. (E) Posterior distribution of the fitness change conferred by mutations that fit acceptance criteria and the size of the tumour when that mutation arose are shown. (F) Posterior distribution of mutations that fit acceptance criteria and the size of the tumour when that mutation arose when fitness is modelled as strong positive selection demonstrate that even tumour-promoting mutations still must arise when the tumour is small. (G) Posterior distribution of mutations that fit acceptance criteria and the size of the tumour when that mutation arose in which mutation rate was increased to model multiple private mutations are shown.

Intrigued by the finding that even small polyps could contain multiple pathogenic mutations at various allelic frequencies, we sought to estimate the size of the tumour at the time these private mutations arose. Using three-dimensional mathematical modelling and ABC, in silico tumours, on reaching a mass of 333 333 crypts, equal to an approximate tumour volume of 10 mm^3^, were virtually sectioned and compared with our cohort of polyps (figure 4A). An acceptance criterion of at least 10% frequency in the sampled region, which represents the limit of detection for targeted sequencing, was used to determine which polyps matched our observed data. The number of detectable private mutations, along with the size of the polyp when that mutation arose, was assessed.

Using these acceptance criteria, only 0.8% (6438/749 822) of all mutations generated were detectable in the section of the tumour used for sampling (see online supplementary table S2). Although private mutations occurred uniformly across all tumour sizes (figure 4B), mutations with a detectable frequency in the virtually sampled region arose exclusively early on in the lifespan of the tumour at a mean size of 30±36 crypts (median=18) (figure 4C). These detectable mutations were associated with modest positive changes in fitness. Mutations that were detectable in the sampled region had a mean fitness change of 17±17% (median=17%) (figure 4E) as compared with a mean change of 0±20% for all mutations generated (figure 4D).

To further investigate the finding that detectable mutations had to arise early, the original parameters were modified so that fitness change was forced to be positive, instead sampled from the normal distribution, N (µ=2, σ=0.2) (see Methods) (see online supplementary figure S2). Even under the conditions of strong positive fitness, mutations that were detectable in the section arose when the tumour was a mean size of 169±157 crypts (median=124) (figure 4F), indicating that although increased fitness does increase the time at which mutations can arise, they still arise relatively early on in the tumour's lifespan. Additionally, to model those polyps that acquired multiple private mutations, an increased mutation rate was used. Again, even with an increased mutation rate, mutations that were detectable in the slice arose when the tumour was small, at a mean size of 30±45 crypts (figure 4G, see online supplementary figures S3 and S4). To test whether a larger tumour size was contributing to this result, an end size of 3333 crypts, 1/100th of the size of the original, was used (see online supplementary figure S5). Under both neutral-centred and strong positive fitness change parameters, detectable mutations arose when the tumour was a mean size of 16±16 crypts (median=11) and 39±31 crypts (median=31), respectively.

## Discussion

Historically, many investigators have believed that colorectal adenocarcinomas form through a stepwise progression from benign adenomatous polyps to premalignant polyps with foci of high-grade dysplasia, to locally invasive cancer and eventually to metastatic disease. This progression was thought to be driven by the sequential accumulation of mutations, perturbing specific genetic pathways at each step in the process: WNT signalling, RAS/RAF signalling, inhibition of apoptosis and TGFβ signalling. While these perturbations themselves are undoubtedly critical to colon tumourigenesis, the timing of the acquisition of these genomic changes is still under investigation and continued debate.

Advances in sequencing technology have revealed vast intratumoral heterogeneity in many solid tumours, including colorectal cancers. These lower frequency mutations were undetectable at the time when this stepwise progression model was hypothesised. The data presented here demonstrate that even small adenomas and HPs can have multiple pathogenic mutations, indicating that mutation profile does not define colon polyp stage. Interestingly, mouse models with simultaneous induction of pathogenic mutations in *Apc*, *Kras* and *Pik3ca* still progress through a histological adenoma-to-carcinoma sequence,^27^ further demonstrating that mutational profile of the tumour is not predictive of the stage of the disease. Similarly, others have shown that mutation profiles are relatively stable even when comparing primary and corresponding metastatic tumour.^28^ In addition to genetic profiles, longitudinal gene expression studies in mouse models show minimal expression differences between the early adenoma and intramucosal carcinoma stage.^3^ Collectively, these data point to non-genetic changes as the benchmarks between tumour stages; however, this conclusion depends on the assumption that all polyps have the same capacity for progression if given enough time.

Basic and clinical research has shown that not all polyps have the same growth fates.^2^ ^3^ ^29^ While the majority of adenomas had public only mutations, consistent with a single expansion model of tumour evolution, from these data alone we cannot determine whether those with subclonal mutations arose via an early second expansion or were the result of a single expansion of a population with early genetic diversity. However, recent sequencing studies of primary colorectal cancers indicate that tumour evolution does not happen in a sequential manner with a selective sweep and instead favours coevolution of multiple populations.^30^ This results in a large number of genetically diverse small populations which likely share some common pathogenic mutations. These observations of the natural history of polyp growth combined with patterns of intratumoral heterogeneity do not fit with the conventional stepwise accumulation of mutations hypothesis. The presence of private, pathogenic and presumably driver mutations in small polyps presented here favours the hypothesis that some tumours are ‘born to be bad’ and form via a ‘Big Bang’, either acquiring additional private driver mutations at the time of transformation or when the tumour is very small.^14^ ^15^

Additional evidence in favour of a ‘Big Bang’ model of tumour growth comes from recent studies investigating differential fitness of cancer cell populations. Both Ling *et al*^31^ and Williams *et al*^32^ report that a significant proportion of human cancers display non-Darwinian or neutral evolutionary dynamics. While Williams *et al* demonstrate that some cancer types do display non-neutral dynamics and while it is conceivable that strong Darwinian selection is occurring at times such as the establishment of metastases and treatment with targeted therapies, the data presented here endorse the notion that the addition of a single oncogenic mutation does not drastically change the fitness advantage of that population resulting in a clonal sweep. Furthermore, Humphries *et al*^33^ used methylation patterns as lineage markers of colon adenomas and concluded that recent clonal sweeps have not occurred, again lending support to a model of neutral or limited positive selection based on genotype alone.

An alternate hypothesis merging both the stepwise accumulation of mutations theory and the ‘Big Bang’ model might be feasible for some tumours: some public and private mutations could occur prior to neoplastic transformation, with the resulting tumour arising from a field of genetically heterogeneous cells.^34^ This mechanism would remove the need for colon polyps to exist for long periods of time to undergo sequential evolution with selective sweeps in the stepwise progression model as well as the need for a greatly increased mutation rate occurring only in the first few neoplastic cell divisions as predicted by the ‘Big Bang’ model. Indeed, mutations in genes associated with tumour progression have been found in patches of epithelial cells which appear histologically normal. Multiple research groups have identified histologically normal-appearing colon epithelial cells containing *KRAS* codon 12 or 13 mutations.^10–12^ Furthermore, multiple groups have found patches of normal-appearing skin, lung and breast cells that contain *TP53* mutations, classically thought of as a late-stage mutation in cancer.^13^

As with most retrospective studies, this study does have some notable limitations. First, because only tissue that was removed by polypectomy at the end of the study was available for analysis, non-tumour tissue was unavailable to distinguish germline variants from somatic variants that were present at a high allelic frequency. To address this issue, only known pathogenic mutations were included due to the lack of normal control. Variants of unknown significance were present, but not included in these analyses. Whole exome sequencing of the tumour with paired normal tissue would have yielded a more complete assessment of the mutation status of these polyps. Second, because of limited sample availability, copy number analysis could not be performed in addition to mutational analysis. An estimate of tumour cell content was done by a board-certified pathologist and was used to adjust the mutant allele frequency. Third, while this study included longitudinal imaging data, tissue was only collected at the conclusion of the study for molecular analyses. Future studies investigating clonal evolution should ideally include multiple time points for tissue collection. Finally, due to unanswered questions regarding basic tumour biology, some assumptions must be made about fitness and growth for in silico modelling. To be unbiased as possible, we allowed for any crypt in the matrix to divide and for the fitness change of each newly acquired mutation to be sampled from a normal distribution. Since the exact location of proliferation or self-renewal within a tumour is not universal and the relative fitness of subclonal neoplastic populations is not known, we believed that these were fair assumptions that could be made for an ABC model, in which knowledge is gained from millions of iterations.

In summary, we have demonstrated that human colon polyps have different growth fates that are independent of specific mutations and that even small polyps can have multiple pathogenic mutations. We further investigated the mechanism of adenoma growth by applying statistical inference to a three-dimensional computer model and found that these additional private, pathogenic mutations likely arise early in the lifespan of a tumour. Understanding the molecular pathway of tumourigenesis and clonal evolution in polyps will allow us to better predict which polyps are likely to progress into adenocarcinomas and which patients are predisposed to developing advanced disease. Understanding the biological phenomenon of tumour formation and evolution may allow for changes in how patients are screened for colon cancer and ultimately decrease the incidence of this disease.
