# Supplementary material for: Subclonal diversity arises early even in small colorectal tumours and contributes to differential growth fates
Source: Gut. 2016 Sep 8;66(12):2132–40. doi: 10.1136/gutjnl-2016-312232 (PMC5342955; doi:10.1136/gutjnl-2016-312232)
Supplement: supplementary file [file gutjnl-2016-312232supp002.pdf]

Sample	Volume Change/year	Adjusted Allelic Frequency	Gene.ID	Codon	Variant Call	Consequence and Notes
PF01	131.00%	37%	APC	1319	frameshift	Codon 1319, Frameshift causing a premature stop at codon 1320
PF04	4.00%	67%	APC	1317	Nonsense	p.E1317*
PF06	-32%	9%	APC	1433	missense	Codon 1433, Missense (P>L) - Deleterious
PF07	0%					no pathogenic identified
PF08	136.40%	48%	APC	1433	frameshift	Codon 1433, Frameshift causing a premature stop
		59%	EGFR	596	missense	p.P596L - Deleterious (activating)
PF11	75.20%					no pathogenic identified
PF12	75%	40%	APC	1322	nonsense	p.E1322*
PF13	59%	5%	FBXW7	393	Nonsense	Stop gained, R>stop (in the Notch pathway) p.R393*
		12%	APC	1433	missense	Missense (P>L), codon 1433
		33%	APC	1467	frameshift	Truncation?, codon p.PX1467X
PF14	47.70%	78%	APC	1309	frameshift	codon p.E1309fs*4
PF15	47.30%	43%	APC	876	nonsense	Truncation, codon 876 p.R876*
		47%	APC	1450	nonsense	Truncation, Codon 1450, p.R1450*
		6%	KRAS	146	missense	Missense variant (ala>val) codon 146, deleterious, p.A146V
PF17	-49.90%	24%	BRAF	600	missense	Codon 600, Missense (V>E) - Pathogenic
PF18	-33.10%	8%	KRAS	12	missense	Missense variant (G/C), codon 12, predicted to be deleterious, p.G12C
		26%	RB1	548	missense	Missense variant (K>N) codon 548, predicted to be deleterious
		5%	TP53	175	missense	Codon 175, Missense (R>H) - Pathogenic p.R175H
PF19	0.00%	36%	APC	1554	frameshift	Insertion at codon 1554, effect unknown
PF20	0.00%	17%	APC	1488	frameshift	p.L1488fs*18, codon 1488
PF21	4.80%	38%	APC	1510	frameshift	Truncation, codon 1510 p.S1510X
PF22	22.00%	37%	APC	1319	truncating frameshift	Codon 1319, Frameshift causing a premature stop at codon 1320 p.P1319fs*2
PF23	35.20%	35%	APC	1286	nonsense	Truncation codon p.E1286*
PF24	32%	86%	APC	1464	truncating frameshift	Truncation codon 1462, p.E1464fs*8, results in stop at 1471
		12%	KRAS	12	missense	p.G12D missense variant, (G>D) codon 12, predicted to be deleterious
PF25	40.30%	58%	APC	876	nonsense	Truncation, codon 876, p.R876*
		55%	APC	1450	nonsense	Truncation, codon 1450, p.R1450*
PF26	42%					no pathogenic identified
PF239	-38.90%	52%	BRAF	600	Missense	Missense (V>E) - Pathogenic
PF241	14.20%	79%	KRAS	12	Missense	Missense variant, (G>V) codon 12, predicted to be deleterious
PF245	66.90%	88%	APC	1309	Frameshift Del	p.E1309fs*4
		92%	RB1	656	Missense	p.R656W SIFT = deleterious, PolyPhen = benign

Sample	Volume Change/year	Adjusted Allelic Frequency	Gene.ID	Codon	Variant Call	Consequence and Notes
PF249	-28.60%	188%	APC	1309	Frameshift Del	p.E1309fs*4
PF250	147.40%	113%	APC	1379	Insertion	p.E1379ETX effect unknown
		34%	KRAS	13	Missense	p.G13D pathogenic
PF252	-27.40%					no pathogenic variants
PF254	20.80%	38%	FBXW7	482	Missense	p.T482A, deleterious by both SIFT and PolyPhen
		116%	TP53	90	missense	p.S?90F?
PF255	17.20%	131%	BRAF	600	Missense	p.V600E
PF256	95.30%	57%	FBXW7	385	Missense	p.R385C
		108%	KRAS	61	Missense	p.Q61L
PF259	3%	205%	APC	1309	Frameshift Del	p.E1309fs*4
		31%	TP53	306	Nonsense	p.R306*
264	-4.40%	46%	APC	1429	Nonsense	p.Q1429*
		41%	APC	1450	Nonsense	p.R1450*
PF270	23.70%	103%	FBXW7	[illegible]26/385/46[illegible]	Missense	p.R385H
		193%	TP53	195	Missense	p.I195T
PF273	42.50%	76%	APC	1455	Frameshift Ins	p.N1455fs*2
PF274	-39.60%	28%	APC	1455	Frameshift Ins	p.N1455fs*2
276	-22.80%	11%	FGFR3	532/644	Missense	p.N532/644D
		10%	FBXW7	482	Missense	p.T482A, deleterious by both SIFT and PolyPhen
		10%	APC	1433	Missense	Missense (P>L) Deleterious
277	66.20%					no pathogenic variants
PF278	-49%	89%	APC	1363	Deletion	p.KS?1363? Out of frame deletion, effect unknown
		89%	APC	1455	Frameshift Ins	p.N1455fs*2
PF279	-21.70%	22%	APC	1451	insertion	p.E1451A?, probably pathogenic?
PF285	12.30%	50%	APC	1291	Nonsense	p.Q1291*
PF287	37.50%	62%	APC	1556	Frameshift del	p.T1556fs*9
PF288	-8.40%	80%	APC	1096	Nonsense	p.Q1096*
PF289	1.00%	150%	APC	1353	Nonsense	p.E1353*
PF290	109.80%	25%	NRAS	12	Missense	p.G12C
		98%	APC	1492	Deletion	ATES?/A deletes a T,E,S at codons 1492, 1493, 1494 ?
PF293	28.30%	10%	APC	1373	Missense	p.P1373L
		12%	APC	1558	Missense	p.D1558N
		45%	BRAF	600	Missense	p.V600E
		9%	KRAS			splice donor variant - predicted to have high impact
PF295	-47.80%	56%	BRAF	600	Missense	p.V600E
PF296	-43.10%	13%	APC	1433	Missense	Missense (P>L) Deleterious
PF296	-43.10%	55%	BRAF	600	Missense	p.V600E
PF297	-29.90%	69%	BRAF	600	Missense	p.V600E
PF298	-20.80%	48%	BRAF	600	Missense	p.V600E

Colors indicate the growth category each polyp was classified as: green = growing polyps (≥20%), orange = static polyps (20% to -20%), red = regressing polyps (≤ -20%).
