# Supplementary material for: Subclonal diversity arises early even in small colorectal tumours and contributes to differential growth fates
Source: Gut. 2016 Sep 8;66(12):2132–40. doi: 10.1136/gutjnl-2016-312232 (PMC5342955; doi:10.1136/gutjnl-2016-312232)
Supplement: supplementary file [file gutjnl-2016-312232supp003.pdf]

Sample	Volume Change	Pathology	Sample Notes	Tumor Cellularity Estimate	Chromosome	Position	Reference	Variant	Allelic Frequency	Adjusted Allelic Frequency	Quality	Origional Coverage	Allele Coverage (# allele reads)	Allele Name	Gene ID	Call	Codon	Annotation
PF01	131.00%	Tubular Adenoma		50%	chr2	212812097	T	C	53%	107%	7560.26	1351	721	---	ERBB4	SNP		Intronic Variant (33%)
				50%	chr4	1807894	G	A	100%	200%	15859.9	995	995	---	FGFR3	SNP		Protein coding, synonymous variant
				50%	chr4	55141055	A	G	100%	200%	28940.2	1813	1813	---	PDGFRA	SNP		Protein coding, synonymous variant
				50%	chr4	55980239	C	T	37%	73%	5257.68	1693	620	---	KDR	SNP		Intronic variant
				50%	chr5	112175247	CT	-	19%	37%	831.42	1769	328	---	APC	Deletion pathogenic	1319	Codon 1319, Frameshift causing a premature stop
				50%	chr5	149433596	T	G	100%	200%	6787.94	594	594	---	CSF1R	SNP		3' UTR variant
				50%	chr5	149433597	G	A	95%	191%	8952.71	625	596	---	CSF1R	SNP		3' UTR variant
				50%	chr7	55249063	G	A	51%	102%	5759.33	1106	565	---	EGFR	SNP		protein coding, synonymous variant
				50%	chr10	43613843	G	T	49%	98%	9850.17	1998	980	---	RET	SNP	769	Codon 769, Synonymous variant (28%)
				50%	chr11	534242	A	G	49%	98%	4824.38	978	481	COSM249860	HRAS	SNP	27	Codon 27, Synonymous variant (30%)
				50%	chr13	28610183	A	G	100%	200%	32002.2	1998	1998	---	FLT3	SNP		Splice region variant (38%)
				50%	chr17	7579472	G	C	50%	99%	4515.7	885	439	---	TP53	Missense tolerated	72	Missense variant, proline to argine, non-pathogenic
				50%	chr18	48586344	C	T	55%	110%	11810.3	2000	1102	---	SMAD4	SNP		Intronic variant
PF03	62.80%	Tubular Adenoma Descending Colon	Control: two small polyps mixed together	35%	chr2	209113192	G	A	51%	145%	10367.7	2000	1013	NOCOSMIC105	IDH1	SNP	105	Codon 105, Synonymous variant (5%)
				35%	chr4	1807894	G	A	100%	286%	18833.3	1180	1180	---	FGFR3	SNP		Protein coding, Synonymous
				35%	chr4	55141055	A	G	100%	286%	31865.7	1988	1988	---	PDGFRA	SNP		Protein coding, synonymous
				35%	chr4	55593464	A	C	49%	140%	9803.62	1997	977	COSM28026	KIT	Missense tolerated	537	Codon 537, Missense (M>L) - Tolerated
				35%	chr4	55962546	-	G	43%	122%	5062.97	1993	848	---	KDR	insertion		Noncoding region, unknown
				35%	chr4	55972974	T	A	48%	138%	9643.22	1994	966	---	KDR	Missense tolerated	472	Codon 472, Missense (Q/H) - Tolerated
				35%	chr4	55980239	C	T	33%	94%	4545.64	1740	573	---	KDR	SNP		Intronic variant
				35%	chr5	112173917	C	T	2%	6%	16.9265	2000	41	COSM18852	APC	nonsense pathogenic	876	Codon 876, Stop gained
				35%	chr5	112175576	C	T	2%	6%	19.0173	1998	42	COSM18836	APC	nonsense pathogenic	1429	Codon 1429, Stop gained
				35%	chr5	112175770	G	A	60%	171%	13460.4	1998	1196	---	APC	SNP	1493	Codon 1493, Synonymous variant
				35%	chr5	149433596	T	G	100%	286%	11239.6	986	986	---	CSF1R	SNP		3' UTR variant
				35%	chr5	149433597	G	A	97%	278%	15140.3	1012	986	---	CSF1R	SNP		3' UTR variant
				35%	chr7	55249063	G	A	100%	286%	20003.1	1262	1262	---	EGFR	SNP		Protein coding, Synonymous
				35%	chr10	43613843	G	T	100%	286%	32012.5	1996	1996	---	RET	SNP	769	Codon 769, Synonymous variant (28%)
				35%	chr11	534242	A	G	44%	127%	5840.92	1388	616	COSM249860	HRAS	SNP	27	Codon 27, Synonymous variant
				35%	chr13	28610183	A	G	100%	286%	32079.4	2000	2000	---	FLT3	SNP		Splice region variant (38%)
				35%	chr17	7579472	G	C	57%	163%	7110.42	1123	639	---	TP53	Missense tolerated	72	Missense variant (P>R) - Non-pathogenic
				35%	chr19	1220321	T	C	52%	150%	6458.69	1192	625	---	STK11	SNP		Intronic variant (35%)
PF04	4.00%	Tubular Adenoma Descending Colon		85%	chr2	209113192	G	A	47%	55%	9235.82	2000	941	NOCOSMIC105	IDH1	SNP	105	Codon 105, Synonymous variant
				85%	chr4	1807894	G	A	100%	118%	23088.4	1452	1452	---	FGFR3	SNP	769	Codon 769, Synonymous variant (28%)
				85%	chr4	55141055	A	G	100%	118%	31813.9	1988	1988	---	PDGFRA	SNP	567	Codon 567, Synonymous variant (4%)
				85%	chr4	55593464	A	C	52%	61%	10788.1	1997	1038	COSM28026	KIT	Missense tolerated	537	Codon 537, Missense (M>L) - Tolerated
				85%	chr4	55972974	T	A	50%	59%	10274.7	1996	1006	---	KDR	Missense tolerated	472	Codon 472, Missense (Q/H) - Tolerated
				85%	chr5	112175240	G	T	57%	67%	10278.4	1654	944	COSM19253	APC	nonsense pathogenic	1317	Codon 1317, Stop gained (E>stop)
				85%	chr5	112175770	G	A	78%	91%	20380.4	1998	1553	---	APC	SNP	1493	Codon 1493, Synonymous variant (34%)
				85%	chr5	149433596	T	G	100%	118%	12312.3	1075	1075	---	CSF1R	SNP		Downstream gene variant (27%)
				85%	chr5	149433597	G	A	97%	114%	16430.8	1110	1075	---	CSF1R	SNP		Downstream gene variant (29%)

				85%	chr7	55249063	G	A	100%	118%	22232.5	1390	1390	---	EGFR	SNP	742	Codon 742, Synonymous variant (42%)
				85%	chr10	43613843	G	T	100%	118%	31936.4	1991	1991	---	RET	SNP	769	Codon 769, Synonymous variant (28%)
				85%	chr11	534242	A	G	47%	56%	7675.63	1655	784	COSM249860	HRAS	SNP	27	Codon 27, Synonymous variant (30%)
				85%	chr13	28610183	A	G	100%	118%	31925.3	1996	1996	---	FLT3	SNP		Splice region variant (38%)
				85%	chr17	7579472	G	C	59%	70%	8137.54	1206	717	---	TP53	Missense tolerated	72	Codon 72, Missense variant (P/R) Tolerated
				85%	chr19	1220321	T	C	49%	58%	7158.33	1447	716	---	STK11	SNP		Intronic variant (35%)
PF05	1157.00%	Invasive Carcinoma	Not used in final dataset because of pathology	35%	chr2	212812097	T	C	61%	174%	6096.98	884	538	---	ERBB4	SNP		Intronic variant (33%)
				35%	chr3	178917005	A	G	66%	189%	7521.24	962	636	---	PIK3CA	SNP		Downstream gene variant (22%)
				35%	chr3	178947827	G	T	36%	102%	5970.27	1999	715	COSM769	PIK3CA	Missense pathogenic	901	Codon 901, Missense (C>F) - Deleterious
				35%	chr4	1807894	G	A	100%	286%	14778.5	927	927	---	FGFR3	SNP	651	Codon 651, Synonymous variant
				35%	chr4	55141055	A	G	100%	286%	22135.5	1385	1385	---	PDGFRA	SNP	567	Codon 567, Synonymous variant
				35%	chr4	55593464	A	C	47%	134%	9128.8	1998	934	COSM28026	KIT	Missense tolerated	541	Codon 541, Missense (M>L) - Tolerated
				35%	chr4	55980239	C	T	100%	286%	12875.1	810	810	---	KDR	SNP		Intronic variant (49%)
				35%	chr5	112175770	G	A	99%	282%	31135	1999	1974	---	APC	SNP	1493	Codon 1493, Synonymous variant (34%)
				35%	chr5	149433597	G	A	31%	87%	2837.92	1226	375	---	CSF1R	SNP		Downstream gene variant (29%)
				35%	chr7	55249063	G	A	55%	159%	6475.81	1085	602	---	EGFR	SNP	742	Codon 742, Synonymous variant (42%)
				35%	chr7	140453136	A	T	25%	71%	3259.2	1994	493	COSM476	BRAF	Missense pathogenic	600	Codon 600, Missense (V>E) - Pathogenic
				35%	chr10	43613843	G	T	57%	162%	12408.6	1997	1135	---	RET	SNP	769	Codon 769, Synonymous variant (28%)
				35%	chr11	534242	A	G	100%	286%	19696.9	1247	1247	COSM249860	HRAS	SNP	27	Codon 27, Synonymous variant
				35%	chr13	28610183	A	G	100%	286%	32037.3	1999	1999	---	FLT3	SNP		Splice region variant (38%)
				35%	chr17	7578406	C	T	37%	106%	6348.42	1999	742	COSM99914	TP53	Missense pathogenic	175	Codon 175, Missense (R>H) - Pathogenic
				35%	chr17	7579472	G	C	93%	265%	13946.1	1014	942	---	TP53	Missense tolerated	72	Codon 72, Missense variant (P>R) - Tolerated
				35%	chr17	7579473	G	C	6%	18%	104.53	1014	63	---	TP53	Missense tolerated	72	Codon 72, Missense variant (P>A) - Tolerated
				35%	chr18	48591919	G	A	23%	66%	2911.01	1998	462	COSM14122	SMAD4	Missense pathogenic	361	Codon 361, Missense (R>H) - Pathogenic
PF06	-32.30%	Tubular Adenoma Ascending Colon		75%	chr2	212812097	T	C	50%	67%	500.97	103	52	---	ERBB4	SNP		Intronic variant (33%)
				75%	chr3	178917005	A	G	100%	133%	1785.86	117	117	---	PIK3CA	SNP		Downstream gene variant (22%)
				75%	chr3	178927410	A	G	61%	81%	1092.86	165	100	---	PIK3CA	Missense tolerated	391	Codon 391, Missense (I>M) - Tolerated
				75%	chr4	1807894	G	A	100%	133%	31887.1	1995	1995	---	FGFR3	SNP	651	Codon 651, Synonymous variant (4%)
				75%	chr4	55141055	A	G	100%	133%	6400.18	407	407	---	PDGFRA	SNP	567	Codon 567, Synonymous variant (4%)
				75%	chr4	55972974	T	A	58%	77%	1904.72	306	177	---	KDR	Missense tolerated	472	Codon 472, Missense (Q>H) - Tolerated
				75%	chr4	55980239	C	T	33%	44%	475.18	183	60	---	KDR	SNP		Intronic variant (48%)
				75%	chr5	112175589	C	T	6%	9%	49.37	371	24	---	APC	Missense Deleterious	1433	Codon 1433, Missense (P>L) - Deleterious
				75%	chr5	149433597	G	A	18%	24%	679.5	700	127	---	CSF1R	SNP		Downstream gene variant (29%)
				75%	chr7	55249063	G	A	51%	68%	1239.17	237	121	---	EGFR	SNP		Intronic variant (42%)
				75%	chr13	28610183	A	G	58%	77%	12684.9	2000	1152	---	FLT3	SNP		Splice region variant (38%)
				75%	chr17	7579472	G	C	59%	78%	986.38	157	92	---	TP53	Missense tolerated	72	Codon 72, Missense (P>R) - Tolerated
				75%	chr19	1220321	T	C	100%	133%	9379.76	596	596	---	STK11	SNP		Intronic variant (35%)
PF07	0%	Tubular Adenoma Sigmoid Colon		65%	1	43815035	C	T	51%	79%	7435.67	1413	725	---	MPL	Missense Deleterious	524	Codon 524, Missense (R>C) - Deleterious, benign
				65%	4	1807894	G	A	100%	154%	15121.5	956	956	---	FGFR3	SNP	651	Codon 651, Synonymous variant
				65%	4	55972974	T	A	100%	154%	10428.7	1327	1327	---	KDR	Missense tolerated	472	Codon 472, Missense (Q>H) - Tolerated

				65%	4	55141055	A	G	51%	78%	21200.1	1994	1015	---	CSF1R	SNP	567	Codon 567, Synonymous variant
				65%	5	112175770	G	A	98%	151%	30794.5	1999	1964	---	APC	SNP	1439	Codon 1493, Synonymous variant
				65%	5	149433596	T	G	100%	154%	5096.06	565	564	---	CSF1R	SNP		Downstream gene variant
				65%	5	149433597	G	A	97%	149%	8848.3	592	574	---	CSF1R	SNP		Downstream gene variant
				65%	10	43613843	G	T	48%	75%	9668.84	1998	968	---	RET	SNP	769	Codon 769, Synonymous variant (28%)
				65%	10	43615633	C	G	50%	77%	7504.11	1476	739	---	RET	SNP	904	Codon 904, Synonymous variant (16%)
				65%	11	534242	A	G	46%	71%	5228.01	1171	541	COSM249860	HRAS	SNP	27	Codon 27, Synonymous variant
				65%	13	28610183	A	G	100%	154%	31886.9	1995	1995	---	FLT3	SNP		Splice region variant (38%)
				65%	17	7579472	G	C	92%	142%	12837.1	942	868	---	TP53	Missense tolerated	72	Codon 72, Missense (P/R) - Tolerated
PF08	136.40%	Tubular Adenoma Sigmoid Colon		55%	chr2	212812097	T	C	41%	74%	3859.67	1060	431	---	ERBB4	SNP		Intronic variant (33%)
				55%	chr4	1807894	G	A	100%	182%	20002.8	1259	1259	---	FGFR3	SNP	651	Codon 651, Synonymous variant (4%)
				55%	chr4	55141055	A	G	100%	182%	20202.5	1270	1270	---	PDGFRA	SNP	567	Codon 567, Synonymous variant (3%)
				55%	chr4	55152040	C	T	47%	85%	9101.31	2000	932	COSM22413	PDGFRA	SNP	584	Codon 584, Synonymous variant (21%)
				55%	chr4	55593464	A	C	50%	90%	10072.8	1995	993	COSM28026	KIT	Missense tolerated	541	Codon 541, Missense (M>L) - Tolerated
				55%	chr4	55962546	-	G	100%	182%	16238.7	1344	1344	---	KDR	insertion		Noncoding region, unknown
				55%	chr4	55972974	T	A	100%	182%	31976.2	1995	1995	---	KDR	Missense tolerated	472	Codon 472, Missense (Q/H) - Tolerated
				55%	chr4	55980239	C	T	100%	182%	14334.2	910	910	---	KDR	SNP		Intronic variant (49%)
				55%	chr5	112175588	C	-	27%	48%	1963.31	1932	512	---	APC	Deletion pathogenic	1433	Codon 1433, Frameshift causing a premature stop
				55%	chr5	112175770	G	A	98%	178%	30546.9	2000	1957	---	APC	SNP	1439	Codon 1493, Synonymous variant (34%)
				55%	chr5	149433597	G	A	21%	38%	1673.7	1352	284	---	CSF1R	SNP		Downstream gene variant
				55%	chr7	55233037	C	T	32%	59%	5123.81	1998	649	COSM21689	EGFR	Missense Deleterious	596	Codon 596, Missesnse (P/L) - Deleterious
				55%	chr10	43613843	G	T	100%	182%	31972.6	1994	1994	---	RET	SNP	769	Codon 769, Synonymous variant (28%)
				55%	chr10	43615633	C	G	56%	101%	7233.27	1197	668	---	RET	SNP	904	Codon 904, Synonymous variant (16%)
				55%	chr11	534242	A	G	47%	86%	6239.18	1341	634	COSM249860	HRAS	SNP	27	Codon 27, Synonymous variant (30%)
				55%	chr13	28610183	A	G	100%	182%	31881.4	1995	1995	---	FLT3	SNP		Splice region variant (38%)
				55%	chr17	7579472	G	C	57%	103%	5807.83	932	528	---	TP53	Missense tolerated	72	Codon 72, Missense variant (P>R) - Tolerated
PF11	75.20%	Tubular Adenoma Ascending Colon	Note: CTNNB1 p.S45F variant (cosmic 5667)failed to be validated by qPCR and was thus eliminated	45%	chr1	43815035	C	T	39%	86%	3410.82	1014	392	---	MPL	Missense pathogenic	524	Missense variant (arg>cys), codon 524, mH3K27Ac mark, conflicting predicted effects
				45%	chr2	209113192	G	A	44%	97%	8215.44	1998	874	NOCOSMIC105	IDH1	SNP	105	Synonymous variant, codon 105
				45%	chr2	212812097	T	C	53%	117%	10989.6	1999	1051	---	ERBB4	SNP		Intronic variant (33%)
				45%	chr3	41266137	C	T	4%	10%	62.02	1998	86	COSM5667	CTNNB1	Missense pathogenic	45	Missense variant (Ser>Phe), pathogenic, codon 45
				45%	chr3	178917005	A	G	41%	91%	7444.32	1998	820	---	PIK3CA	SNP		Downstream gene variant (22%)
				45%	chr4	1807894	G	A	100%	222%	7109.2	453	453	---	FGFR3	SNP	651	Codon 651, Synonymous variant (4%)
				45%	chr4	55141055	A	G	100%	222%	31912	1991	1991	---	PDGFRA	SNP	567	Codon 567, Synonymous variant (4%)
				45%	chr4	55152040	C	T	81%	181%	22040.4	1998	1628	COSM22413	PDGFRA	SNP		Codon 584, Synonymous variant (21%)
				45%	chr4	55972974	T	A	42%	93%	7615.76	1992	831	---	KDR	Missense tolerated	472	Codon 472, Missense (Q>H) - Tolerated
				45%	chr5	112175770	G	A	91%	202%	26451.2	1998	1812	---	APC	SNP	1493	Synonymous, codon 1493
				45%	chr5	149433597	G	A	70%	156%	8096.22	935	657	---	CSF1R	SNP		Downstream gene variant
				45%	chr7	55249063	G	A	54%	121%	4889.59	843	459	---	EGFR	SNP		Intronic variant (42%)

				45%	chr10	43613843	G	T	84%	186%	21910.2	1900	1594	---	RET	SNP	769	Codon 769, Synonymous variant (28%)
				45%	chr10	43615633	C	G	37%	82%	4266.92	1368	504	---	RET	SNP	904	Codon 904, Synonymous variant (16%)
				45%	chr13	28602292	T	C	41%	91%	7448.02	1995	821	---	FLT3	SNP		Intronic variant (4%)
				45%	chr13	28610183	A	G	93%	206%	27621.2	2000	1858	---	FLT3	SNP		Splice region variant (38%)
				45%	chr17	7579472	G	C	65%	145%	5109.71	672	437	---	TP53	Missense tolerated	72	Codon 72, Missense (P/R) - Tolerated
				45%	chr19	1220321	T	C	18%	41%	391.55	391	72	---	STK11	SNP		Intronic variant (35%)
PF12	75.00%	Tubular Adenoma Sigmoid Colon		80%	chr2	212812097	T	C	57%	71%	9502.44	1540	872	---	ERBB4	SNP		Intronic variant (33%)
				80%	chr4	1807894	G	A	100%	125%	19470.8	1226	1226	---	FGFR3	SNP	651	Codon 651, Synonymous variant (4%)
				80%	chr4	55141055	A	G	100%	125%	31802.1	1991	1991	---	PDGFRA	SNP	567	Codon 567, Synonymous variant (4%)
				80%	chr4	55962546	-	G	46%	57%	5704.05	1983	903	---	KDR	Insertion		Noncoding region, unknown
				80%	chr4	55972974	T	A	52%	65%	10704.7	1993	1032	---	KDR	Missense tolerated	472	Codon 472, Missense (Q>H) - Tolerated
				80%	chr5	112175255	G	T	32%	40%	5039.7	2000	643	COSM18702	APC	Nonsense pathogenic	1322	Truncation, codon 1322 (E>stop)
				80%	chr5	112175770	G	A	99%	124%	31251.5	1993	1974	---	APC	SNP	1493	Synonymous, codon 1493
				80%	chr5	149433597	G	A	32%	40%	5041.74	1999	643	---	CSF1R	SNP		Downstream gene variant
				80%	chr7	55249063	G	A	50%	63%	5562.47	1084	546	---	EGFR	SNP		Intronic variant (42%)
				80%	chr10	43613843	G	T	100%	125%	31970.9	1993	1993	---	RET	SNP	769	Codon 769, Synonymous variant (28%)
				80%	chr11	108138003	T	C	49%	61%	9905.56	1999	983	COSM21826	ATM	Missense tolerated	858	Missense variant (phe>leu) protein position 858, tolerated
				80%	chr13	28610183	A	G	100%	125%	31986.1	1997	1997	---	FLT3	SNP		Splice region variant (38%)
				80%	chr17	7579472	G	C	96%	120%	15461.7	1044	1006	---	TP53	Missense tolerated	72	Codon 72, Missense (P/R) - Tolerated
				80%	chr19	1220321	T	C	44%	55%	4102.36	994	436	---	STK11	SNP		Intronic variant (35%)
PF13	58.80%	Tubular Adenoma		78%	chr4	1807894	G	A	100%	128%	31725.4	1986	1986	---	FGFR3	SNP	651	Codon 651, Synonymous variant
				78%	chr4	55141055	A	G	100%	128%	16443.4	1031	1031	---	PDGFRA	SNP	567	Codon 567, Synonymous variant (4%)
				78%	chr4	55946354	G	T	49%	63%	9830.42	2000	980	---	KDR	SNP		Intronic variant
				78%	chr4	55962546	-	G	100%	128%	24427.9	1987	1987	---	KDR	Insertion unknown		Noncoding region, unknown
				78%	chr4	55972974	T	A	100%	128%	10160.9	639	639	---	KDR	Missense tolerated	472	Codon 472, Missense (Q>H) - Tolerated
				78%	chr4	153250883	G	A	4%	5%	42.61	1996	78	COSM22973	FBXW7	Nonsense pathogenic?	393	Stop gained, R>stop (in the Notch pathway)
				78%	chr5	112175589	C	T	9%	12%	430.31	1592	151	---	APC	Missense Deleterious	1433	Codon 1433, Missense (P>L) - Deleterious
				78%	chr5	112175690	CC	-	26%	33%	1779.84	1849	475	---	APC	Deletion unknown	1467	frameshift variant, codon 1467
				78%	chr5	112175770	G	A	50%	64%	6133.12	1213	607	---	APC	SNP	1493	Synonymous, codon 1493
				78%	chr5	149433596	T	G	100%	128%	9068.15	793	793	---	CSF1R	SNP		Downstream gene variant
				78%	chr5	149433597	G	A	97%	125%	12172.3	814	791	---	CSF1R	SNP		Downstream gene variant
				78%	chr7	55249063	G	A	100%	128%	7589.93	479	479	---	EGFR	SNP		Intronic variant (42%)
				78%	chr11	534242	A	G	48%	61%	9380.27	1995	949	COSM249860	HRAS	SNP	27	Codon 27, Synonymous variant (30%)
				78%	chr13	28610183	A	G	100%	128%	31970.2	1996	1996	---	FLT3	SNP		Splice region variant (38%)
				78%	chr17	7579472	G	C	95%	122%	3939.05	276	263	---	TP53	Missense tolerated	72	Codon 72, Missense (P/R) - Tolerated
				78%	chr19	1220321	T	C	46%	59%	3948.08	897	413	---	STK11	SNP		Intronic variant (35%)
				78%	chr19	1223125	C	G	51%	65%	5353.21	1031	524	COSM21360	STK11	Missense pathogenic	110/354	Missense variant (phe>leu) reported to be associated with Peutz-Jeghers syndrome
PF14	47.70%	Tubular Adenoma Rectum		90%	chr3	178917005	A	G	49%	55%	7460.72	1513	745	---	PIK3CA	SNP		Downstream gene variant (22%)
				90%	chr4	1807894	G	A	100%	111%	24012.5	1509	1509	---	FGFR3	SNP	651	Codon 651, Synonymous variant (4%)
				90%	chr4	55141055	A	G	100%	111%	31842.3	1988	1988	---	PDGFRA	SNP	567	Codon 567, Synonymous variant (4%)
				90%	chr4	55972974	T	A	50%	56%	10273.7	1997	1006	---	KDR	Missense tolerated	472	Codon 472, Missense (Q>H) - Tolerated

				90%	chr5	112175216	GAAAA	-	71%	78%	9776.48	1544	1090	COSM19145	APC	Missense pathogenic	1307	Truncation, codon 1307
				90%	chr5	112175217	AAAAG	-	71%	78%	9776.48	1544	1090	COSM18701	APC	Missense pathogenic	1307	Truncation, codon 1307
				90%	chr5	112175213	AAAGA	-	71%	78%	9776.48	1544	1090	COSM19263	APC	Missense pathogenic	1307	Truncation, codon 1307
				90%	chr5	112175218	AAAGA	-	71%	78%	9776.48	1544	1090	COSM13113	APC	Missense pathogenic	1307	Truncation, codon 1307
				90%	chr5	112175212	AAAAG	-	71%	78%	9776.48	1544	1090	COSM18764	APC	Missense pathogenic	1307	Truncation, codon 1307
				90%	chr5	112175770	G	A	99%	110%	31159.2	1996	1973	---	APC	SNP	1493	Synonymous, codon 1493
				90%	chr5	149433596	T	G	100%	111%	12205.4	1068	1068	---	CSF1R	SNP		Downstream gene variant
				90%	chr5	149433597	G	A	97%	108%	16431.7	1109	1074	---	CSF1R	SNP		Downstream gene variant
				90%	chr7	55249063	G	A	100%	111%	20626.3	1293	1293	---	EGFR	SNP		Intronic variant (42%)
				90%	chr10	43613843	G	T	54%	60%	11373.1	1998	1075	---	RET	SNP	769	Codon 769, Synonymous variant (28%)
				90%	chr11	534242	A	G	50%	56%	8545.54	1685	842	COSM249860	HRAS	SNP	27	Codon 27, Synonymous variant (30%)
				90%	chr13	28610183	A	G	100%	111%	31992.9	1996	1996	---	FLT3	SNP		Splice region variant (38%)
				90%	chr17	7579472	G	C	96%	107%	14422.4	980	941	---	TP53	Missense tolerated	72	Codon 72, Missense (P/R) - Tolerated
PF15	47.30%	Tubular Adenoma Cecum		55%	chr2	212812097	T	C	67%	122%	7642.22	946	635	---	ERBB4	SNP		Intronic variant (33%)
				55%	chr4	1807894	G	A	100%	182%	18628.5	1170	1170	---	FGFR3	SNP	651	Codon 651, Synonymous variant (4%)
				55%	chr4	55141055	A	G	100%	182%	31604.7	1983	1983	---	PDGFRA	SNP	567	Codon 567, Synonymous variant (4%)
				55%	chr4	55980239	C	T	30%	55%	2475.25	1102	331	---	KDR	SNP		Intronic variant (48%)
				55%	chr5	112173917	C	T	24%	43%	3018.88	2000	472	COSM18852	APC	Nonsense pathogenic	876	Truncation, codon 876, (R>stop)
				55%	chr5	112175639	C	T	26%	47%	3513.93	1996	516	COSM13127	APC	Nonsense pathogenic	1450	Truncation, Codon 1450 (R>stop)
				55%	chr5	112175770	G	A	98%	179%	30829.6	1997	1964	---	APC	SNP	1439	Synonymous, codon 1493
				55%	chr5	149433596	T	G	99%	181%	12344.8	1104	1098	---	CSF1R	SNP		Downstream gene variant
				55%	chr5	149433597	G	A	98%	178%	17211.8	1127	1105	---	CSF1R	SNP		Downstream gene variant
				55%	chr10	43613843	G	T	100%	182%	31946.4	1992	1992	---	RET	SNP	769	Codon 769, Synonymous variant (28%)
				55%	chr10	43615633	C	G	52%	94%	10727.1	1997	1034	---	RET	SNP	904	Codon 904, Synonymous variant (16%)
				55%	chr12	25378561	G	A	3%	6%	11.85	1536	47	COSM19900	KRAS	Missense deleterious	146	Missense variant (ala>val) codon 146, deleterious
				55%	chr13	28610183	A	G	55%	100%	11751.5	2000	1097	---	FLT3	SNP		Splice region variant (38%)
				55%	chr17	7579472	G	C	98%	179%	16904.8	1097	1078	---	TP53	Missense tolerated	72	Codon 72, Missense (P/R) - Tolerated
				55%	chr19	1220321	T	C	52%	94%	5859.56	1090	565	---	STK11	SNP		Intronic variant (35%)
PF17	-49.90%	Hyperplastic Polyp Rectum		90%	chr3	178917005	A	G	51%	57%	5440.2	1030	529	---	PIK3CA	SNP		Downstream gene variant (22%)
				90%	chr4	1807894	G	A	100%	111%	15065.3	944	944	---	FGFR3	SNP	651	Codon 651, Synonymous variant (4%)
				90%	chr4	55141055	A	G	100%	111%	29010.9	1820	1820	---	PDGFRA	SNP	567	Codon 567, Synonymous variant (4%)
				90%	chr5	112175770	G	A	52%	58%	10833	1999	1041	---	APC	SNP	1439	synonymous variant, codon 1493
				90%	chr7	55249063	G	A	100%	111%	12490.9	783	783	---	EGFR	SNP		Intronic variant (42%)
				90%	chr7	140453136	A	T	21%	24%	2545.31	1999	427	COSM476	BRAF	Missense pathogenic	600	Codon 600, Missense (V>E) - Pathogenic
				90%	chr10	43613843	G	T	100%	111%	31935.7	1993	1993	---	RET	SNP	769	Codon 769, Synonymous variant (28%)
				90%	chr13	28610183	A	G	54%	60%	11405.9	2000	1076	---	FLT3	SNP		Splice region variant (38%)
				90%	chr17	7579472	G	C	60%	67%	4270.51	627	376	---	TP53	Missense tolerated	72	Codon 72, Missense (P/R) - Tolerated
PF18	-33%	Tubular Adenoma Rectum		83%	chr3	178917005	A	G	49%	59%	8842.82	1809	885	---	PIK3CA	SNP		Downstream gene variant (22%)
				83%	chr4	1807894	G	A	100%	120%	18399.1	1158	1158	---	FGFR3	SNP	651	Codon 651, Synonymous variant (4%)
				83%	chr4	55141055	A	G	100%	120%	31775.4	1988	1988	---	PDGFRA	SNP	567	Codon 567, Synonymous variant (4%)
				83%	chr4	55946171	G	A	49%	59%	3716.87	747	368	---	KDR	SNP		Synonymous variant (1.5%) codon 1336

				83%	chr4	55972974	T	A	48%	58%	9456.5	1995	954	---	KDR	Missense tolerated	472	Codon 472, Missense (Q>H) - Tolerated
				83%	chr4	55980239	C	T	100%	120%	13465.9	843	843	---	KDR	SNP		Intronic variant (48%)
				83%	chr5	149433596	T	G	100%	120%	7232.03	610	610	---	CSF1R	SNP		Downstream gene variant
				83%	chr5	149433597	G	A	96%	116%	9207.62	633	609	---	CSF1R	SNP		Downstream gene variant
				83%	chr7	55249063	G	A	100%	120%	13489	852	852	---	EGFR	SNP		Intronic variant (42%)
				83%	chr10	43613843	G	T	51%	61%	10379.9	1999	1013	---	RET	SNP		Codon 769, Synonymous variant (28%)
				83%	chr11	534242	A	G	49%	59%	6976.82	1421	697	COSM249860	HRAS	SNP	27	Codon 27, Synonymous variant (30%)
				83%	chr12	25398285	C	A	6%	8%	215.59	1991	129	COSM516	KRAS	Missense	12	Missense variant (G/C), codon 12,
				83%	chr13	28610183	A	G	100%	120%	31871.5	1994	1994	---	FLT3	SNP		Splice region variant (38%)
				83%	chr13	48955528	A	C	21%	26%	2507.28	2000	424	---	RB1	Missense	548	Missense variant (K>N) codon
				83%	chr17	7578406	C	T	4%	5%	40.06	2000	77	COSM99914	TP53	Missense pathogenic	175	Codon 175, Missense (R>H) - Pathogenic
				83%	chr17	7579472	G	C	45%	54%	3521.77	792	357	---	TP53	Missense tolerated	72	Codon 72, Missense (P/R) - Tolerated
PF19	0.00%	Tubular Adenoma Distal Traverse		45%	chr2	212812097	T	C	54%	120%	11512.3	1998	1082	---	ERBB4	SNP		Intronic variant (33%)
				45%	chr4	1807894	G	A	100%	222%	15840.3	1001	1001	---	FGFR3	SNP	651	Codon 651, Synonymous variant (4%)
				45%	chr4	55141055	A	G	100%	222%	31642.9	1979	1979	---	PDGFRA	SNP	567	Codon 567, Synonymous variant (4%)
				45%	chr4	55962546	-	G	44%	99%	5481.9	1989	884	---	KDR	insertion		Noncoding region, unknown
				45%	chr4	55972974	T	A	48%	107%	9541.46	1993	959	---	KDR	Missense tolerated	472	Codon 472, Missense (Q>H) - Tolerated
				45%	chr4	55980239	C	T	33%	74%	3970.61	1487	496	---	KDR	SNP		Intronic variant (48%)
				45%	chr5	112175770	G	A	47%	104%	9165.54	2000	936	---	APC	SNP	1493	Synonymous, codon 1493
				45%	chr5	112175952	-	A	16%	36%	258.44	1966	315	---	APC	insertion	1554	Insertion at codon 1554, effect unknown
				45%	chr5	149433596	T	G	100%	222%	8567.36	745	743	---	CSF1R	SNP		Downstream gene variant
				45%	chr5	149433597	G	A	97%	215%	11444.4	771	747	---	CSF1R	SNP		Downstream gene variant
				45%	chr7	55249063	G	A	100%	222%	13146.8	828	828	---	EGFR	SNP		Intronic variant (42%)
				45%	chr10	43613843	G	T	49%	108%	9714.32	1993	970	---	RET	SNP	769	Codon 769, Synonymous variant (28%)
				45%	chr11	534242	A	G	50%	112%	5350.6	1043	525	COSM249860	HRAS	SNP	27	Codon 27, Synonymous variant (30%)
				45%	chr13	28610183	A	G	100%	222%	32024.8	1998	1998	---	FLT3	SNP		Splice region variant (38%)
				45%	chr17	7579472	G	C	54%	120%	2985.83	515	277	---	TP53	Missense tolerated	72	Codon 72, Missense (P/R) - Tolerated
PF20	0.00%	Tubular Adenoma Cecum		78%	chr3	178917005	A	G	100%	128%	31491.3	1983	1983	---	PIK3CA	SNP		Downstream gene variant (22%)
				78%	chr4	1807894	G	A	100%	128%	16567.7	1038	1038	---	FGFR3	SNP	651	Codon 651, Synonymous variant (4%)
				78%	chr4	55141055	A	G	100%	128%	31589.5	1982	1982	---	PDGFRA	SNP	567	Codon 567, Synonymous variant (4%)
				78%	chr4	55973048	G	A	52%	66%	10610.8	1996	1030	---	KDR	SNP		Intronic variant
				78%	chr5	112175754	TATT	-	13%	17%	387.04	1981	262	COSM41618	APC	deletion pathogenic	1488	Truncation, codon 1488
				78%	chr5	149433596	T	G	100%	128%	9657.23	822	822	---	CSF1R	SNP		Downstream gene variant
				78%	chr5	149433597	G	A	98%	126%	12802.2	843	828	---	CSF1R	SNP		Downstream gene variant
				78%	chr7	55249063	G	A	100%	128%	13941.8	877	877	---	EGFR	SNP		Intronic variant (42%)
				78%	chr10	43613843	G	T	50%	63%	9985.02	1993	987	---	RET	SNP	769	Codon 769, Synonymous variant (28%)
				78%	chr11	534242	A	G	50%	65%	6769.56	1322	666	COSM249860	HRAS	SNP	27	Codon 27, Synonymous variant (30%)
				78%	chr13	28610183	A	G	100%	128%	31980.3	1998	1998	---	FLT3	SNP		Splice region variant (38%)
				78%	chr17	7579472	G	C	97%	124%	9948.04	671	649	---	TP53	Missense tolerated	72	Codon 72, Missense (P/R) - Tolerated
				78%	chr22	24143260	C	T	7%	9%	87.44	1627	115	---	SMARCB1	SNP		Synonymous variant
PF21	4.80%	Tubular Adenoma Transverse Colon		45%	chr2	29432625	C	A	32%	71%	2774.88	1116	357	---	ALK	SNP		Intronic variant (10%)
				45%	chr4	1807894	G	A	100%	222%	13453.1	852	852	---	FGFR3	SNP	651	Codon 651, Synonymous variant (4%)
				45%	chr4	55141055	A	G	100%	222%	31505.9	1979	1979	---	PDGFRA	SNP	567	Codon 567, Synonymous variant (4%)
				45%	chr4	55593481	A	G	52%	115%	10716.4	2000	1034	COSM21983	KIT	SNP		Synonymous variant (2.2%)
				45%	chr5	112175770	G	A	99%	220%	31295.9	1998	1978	---	APC	SNP	1493	Synonymous, codon 1493

				45%	chr5	112175821	C	-	17%	38%	775.94	1986	342	---	APC	deletion pathogenic	1510	Truncation, codon 1510
				45%	chr10	43613843	G	T	51%	114%	10577.3	1994	1025	---	RET	SNP	769	Codon 769, Synonymous variant (28%)
				45%	chr11	534242	A	G	48%	108%	4688.22	975	472	COSM249860	HRAS	SNP	27	Codon 27, Synonymous variant (30%)
				45%	chr13	28610183	A	G	100%	222%	32063	1999	1999	---	FLT3	SNP		Splice region variant (38%)
				45%	chr17	7579472	G	C	60%	133%	3113.73	459	275	---	TP53	Missense tolerated	72	Codon 72, Missense (P/R) - Tolerated
				45%	chr19	1220321	T	C	47%	105%	2860.65	622	293	---	STK11	SNP		Intronic variant (35%)
				45%	chr19	1223125	C	G	52%	116%	6077.35	1126	587	COSM21360	STK11	Missense pathogenic	110/354	Missense variant (phe>leu) reported to be associated with Peutz-Jeghers syndrome
PF22	22.00%	Tubulovillous Adenoma		83%	chr2	212812097	T	C	57%	68%	7251.47	1178	667	---	ERBB4	SNP		Intronic variant (33%)
				83%	chr4	1807894	G	A	100%	120%	17489	1102	1102	---	FGFR3	SNP	651	Codon 651, Synonymous variant (4%)
				83%	chr4	55141055	A	G	100%	120%	31829.6	1987	1987	---	PDGFRA	SNP	567	Codon 567, Synonymous variant (4%)
				83%	chr4	55972974	T	A	48%	57%	9413.89	1998	952	---	KDR	Missense tolerated	472	Codon 472, Missense (Q>H) - Tolerated
				83%	chr5	112175247	C	-	31%	37%	1887.78	1337	416	COSM18700	APC	Deletion pathogenic	1319	Truncation, codon 1319
				83%	chr5	112175247	C	-	31%	37%	1887.78	1337	416	COSM32201	APC	Deletion pathogenic	1319	Truncation, codon 1319
				83%	chr5	112175770	G	A	49%	59%	9920.58	1998	984	---	APC	SNP	1439	Synonymous, codon 1493
				83%	chr5	149433596	T	G	100%	120%	9848.81	857	857	---	CSF1R	SNP		Downstream gene variant
				83%	chr5	149433597	G	A	99%	119%	13347.4	872	859	---	CSF1R	SNP		Downstream gene variant
				83%	chr10	43613843	G	T	50%	60%	10096.1	1993	994	---	RET	SNP	769	Codon 769, Synonymous variant (28%)
				83%	chr10	43615633	C	G	51%	62%	10207.8	1939	992	---	RET	SNP	904	Codon 904, Synonymous variant (16%)
				83%	chr13	28610183	A	G	100%	120%	31893.1	1994	1994	---	FLT3	SNP		Splice region variant (38%)
				83%	chr17	7579472	G	C	50%	60%	3093.87	591	296	---	TP53	Missense tolerated	72	Codon 72, Missense (P/R) - Tolerated
				83%	chr18	48586344	C	T	100%	120%	31922.8	1993	1993	---	SMAD4	SNP		Intronic variant (5%)
				83%	chr22	24176287	G	A	48%	58%	4709.92	980	474	COSM1090	SMARCB1	SNP		Intronic variant (11%)
				83%	chr22	24176330	G	A	35%	43%	2814.09	950	337	---	SMARCB1	Missense deleterious		Missense variant (R>Q), predicted to be deleterious (tumor suppressor)
PF23	35.20%	Tubular Adenoma Sigmoid Colon		65%	chr2	212578395	G	A	3%	5%	17.62	1983	64	---	ERBB4	SNP		Intronic variant
				65%	chr3	178917005	A	G	52%	80%	7331.12	1375	711	---	PIK3CA	SNP		Downstream gene variant (22%)
				65%	chr4	1807894	G	A	100%	154%	22249.8	1401	1401	---	FGFR3	SNP	651	Codon 651, Synonymous variant (4%)
				65%	chr4	55141055	A	G	100%	154%	31527.7	1975	1975	---	PDGFRA	SNP	567	Codon 567, Synonymous variant (4%)
				65%	chr4	55946171	G	A	53%	81%	6928.87	1265	666	---	KDR	SNP	1336	Synonymous variant (1.5%) codon 1336
				65%	chr4	55972974	T	A	49%	75%	9812.31	1997	977	---	KDR	Missense tolerated	472	Codon 472, Missense (Q>H) - Tolerated
				65%	chr4	55980239	C	T	99%	152%	11454.9	735	727	---	KDR	SNP		Intronic variant (48%)
				65%	chr5	112175147	G	T	23%	35%	2465.03	1713	394	---	APC	Nonsense pathogenic	1286	Truncation codon 1286, (E>stop)
				65%	chr5	149433596	T	G	100%	154%	12104.1	1059	1059	---	CSF1R	SNP		Downstream gene variant
				65%	chr5	149433597	G	A	97%	149%	16314	1087	1056	---	CSF1R	SNP		Downstream gene variant
				65%	chr7	55249063	G	A	100%	154%	17121.4	1080	1079	---	EGFR	SNP		Intronic variant (42%)
				65%	chr10	43613843	G	T	50%	77%	10143.8	1995	998	---	RET	SNP	769	Codon 769, Synonymous variant (28%)
				65%	chr11	534242	A	G	50%	77%	9801.53	1940	967	COSM249860	HRAS	SNP	27	Codon 27, Synonymous variant (30%)
				65%	chr13	28610183	A	G	100%	154%	32008.9	1997	1997	---	FLT3	SNP		Splice region variant (38%)
				65%	chr17	7579472	G	C	61%	94%	5186.55	734	450	---	TP53	Missense tolerated	72	Codon 72, Missense (P/R) - Tolerated
PF24	31.90%	Tubulovillous Adenoma Sigmoid		28%	chr1	43815035	C	T	50%	180%	6811.53	1326	669	---	MPL	Missense pathogenic	524	Missense variant (arg>cys), codon 524, mH3K27Ac mark, conflicting predicted effects

		Colon		28%	chr4	1807894	G	A	100%	357%	18168.1	1147	1147	---	FGFR3	SNP	651	Codon 651, Synonymous variant (4%)
				28%	chr4	55141055	A	G	100%	357%	31680.2	1983	1983	---	PDGFRA	SNP	657	Codon 567, Synonymous variant (4%)
				28%	chr4	55962546	-	G	44%	158%	5410.91	1985	877	---	KDR	insertion unknown		Noncoding region, unknown
				28%	chr4	55972974	T	A	48%	171%	9517.14	1996	958	---	KDR	Missense tolerated	472	Codon 472, Missense (Q>H) - Tolerated
				28%	chr4	55980239	C	T	26%	92%	1969.42	1135	291	---	KDR	SNP		Intronic variant (48%)
				28%	chr5	112175677	GAGA	-	24%	86%	1618.88	1932	466	COSM19694	APC	Deletion pathogenic	1462	Truncation codon 1462
				28%	chr5	112175679	GAGA	-	24%	86%	1618.88	1932	466	COSM41622	APC	Deletion pathogenic	1462	Truncation codon 1,462
				28%	chr5	112175682	AGAG	-	24%	86%	1618.88	1932	466	COSM18838	APC	Deletion pathogenic	1462	Truncation codon 1,462
				28%	chr5	112175770	G	A	100%	357%	31449	1983	1983	---	APC	SNP	1493	Synonymous, codon 1493
				28%	chr5	149433596	T	G	100%	356%	8629.36	1278	1275	---	CSF1R	SNP		Downstream gene variant
				28%	chr5	149433597	G	A	98%	350%	21353	1397	1368	---	CSF1R	SNP		Downstream gene variant
				28%	chr10	43613843	G	T	50%	179%	10136.9	1994	997	---	RET	SNP	769	Codon 769, Synonymous variant (28%)
				28%	chr10	43615633	C	G	50%	179%	10207.9	1995	1002	---	RET	SNP	904	Codon 904, Synonymous variant (16%)
				28%	chr11	534242	A	G	50%	177%	7799.2	1564	775	COSM249860	HRAS	SNP	27	Codon 27, Synonymous variant (30%)
				28%	chr12	25398284	C	T	3%	12%	18.78	1995	65	COSM521	KRAS	Missense	12	missense variant, (G>D) codon
				28%	chr13	28610183	A	G	100%	357%	31778.3	1991	1991	---	FLT3	SNP		Splice region variant (38%)
				28%	chr17	7579472	G	C	97%	345%	12388.8	832	804	---	TP53	Missense tolerated	72	Codon 72, Missense (P>R) - Tolerated
PF25	40.30%	Tubular Adenoma Ascending Colon		30%	chr2	212812097	T	C	53%	178%	11226.9	1999	1065	---	ERBB4	SNP		Intronic variant (33%)
				30%	chr3	178917005	A	G	99%	330%	31224.6	1998	1976	---	PIK3CA	SNP		Downstream gene variant (22%)
				30%	chr3	178927410	A	G	55%	184%	11874.4	2000	1104	---	PIK3CA	Missense tolerated	391	Codon 391, Missense (I>M) - Tolerated
				30%	chr4	1807894	G	A	100%	333%	16946.7	1068	1068	---	FGFR3	SNP	651	Codon 651, Synonymous variant (4%)
				30%	chr4	55141055	A	G	100%	333%	31759.8	1986	1986	---	PDGFRA	SNP	567	Codon 567, Synonymous variant (4%)
				30%	chr4	55962546	-	G	45%	150%	5605.1	1991	896	---	KDR	insertion unknown		Noncoding region, unknown
				30%	chr4	55972974	T	A	50%	166%	10094	1992	994	---	KDR	Missense tolerated	472	Codon 472, Missense (Q>H) - Tolerated
				30%	chr4	55980239	C	T	30%	101%	3495.64	1530	464	---	KDR	SNP		Intronic variant (48%)
				30%	chr5	112173917	C	T	18%	58%	1792.85	1999	350	COSM18852	APC	Nonsense pathogenic	876	Truncation, codon 876, (R>stop)
				30%	chr5	112175639	C	T	17%	55%	1629.62	1998	332	COSM13127	APC	Nonsense pathogenic	1450	Truncation, Codon 1450 (R>stop)
				30%	chr5	149433597	G	A	26%	85%	3075.71	1782	456	---	CSF1R	SNP		Downstream gene variant
				30%	chr7	55249063	G	A	46%	152%	3950.15	903	413	---	EGFR	SNP		Intronic variant (42%)
				30%	chr13	28610183	A	G	56%	187%	12131.4	1999	1119	---	FLT3	SNP		Splice region variant (38%)
				30%	chr17	7579472	G	C	62%	205%	4240.01	601	370	---	TP53	Missense tolerated	72	Codon 72, Missense (P/R) - Tolerated
				30%	chr19	1220321	T	C	100%	333%	10189.4	648	648	---	STK11	SNP		Intronic variant (35%)
PF26	42.30%	Tubular Adenoma Rectum		83%	chr2	212812097	T	C	50%	60%	10222.4	1998	1003	---	ERBB4	SNP		Intronic variant (33%)
				83%	chr4	1807894	G	A	100%	120%	17163.1	1075	1075	---	FGFR3	SNP	651	Codon 651, Synonymous variant (4%)
				83%	chr4	55141055	A	G	100%	120%	31495.5	1974	1974	---	PDGFRA	SNP	567	Codon 567, Synonymous variant (4%)
				83%	chr4	55152040	C	T	98%	118%	30764.7	1998	1963	COSM22413	PDGFRA	SNP	584	Codon 584, Synonymous variant (21%)
				83%	chr4	55593464	A	C	51%	62%	10524.6	1999	1022	COSM28026	KIT	Missense tolerated	541	Codon 541, Missense (M>L) - Tolerated
				83%	chr4	55972974	T	A	48%	58%	9593.12	1991	962	---	KDR	Missense tolerated	472	Codon 472, Missense (Q>H) - Tolerated
				83%	chr4	55980239	C	T	100%	120%	14041	885	885	---	KDR	SNP		Intronic variant (48%)
				83%	chr5	112175770	G	A	99%	119%	31011	2000	1971	---	APC	SNP	1493	Synonymous, codon 1493
				83%	chr5	149433597	G	A	26%	31%	2530.72	1441	372	---	CSF1R	SNP		Downstream gene variant

				83%	chr7	55249063	G	A	52%	63%	5183.54	949	496	---	EGFR	SNP		Intronic variant (42%)
				83%	chr10	43613843	G	T	100%	120%	31878.5	1990	1990	---	RET	SNP	769	Codon 769, Synonymous variant (28%)
				83%	chr10	43615633	C	G	100%	120%	28928.4	1813	1813	---	RET	SNP	904	Codon 904, Synonymous variant (16%)
				83%	chr11	534242	A	G	46%	56%	5668.55	1265	586	COSM249860	HRAS	SNP	27	Codon 27, Synonymous variant (30%)
				83%	chr13	28610183	A	G	100%	120%	31982.6	1997	1997	---	FLT3	SNP		Splice region variant (38%)
				83%	chr17	7579472	G	C	95%	115%	8978.33	623	594	---	TP53	Missense tolerated	72	Codon 72, Missense (P/R) - Tolerated
				83%	chr17	7579473	G	C	4%	4%	13.84	622	22	---	TP53	Missense tolerated	72	Codon 72, Missense variant (P>A) - Tolerated
				83%	chr19	1220321	T	C	46%	56%	2880.82	655	302	---	STK11	SNP		Intronic variant (35%)
				83%	chr22	24143260	C	T	6%	8%	69.45	1324	86	---	SMARCB1	SNP		Synonymous variant

All variants post quality control processing. Variants were eliminated based on the following: (1) quality score less than 10, (2)allelic frequency less than 5% or number of allele reads less than 10, (3) a known mispriming event for this panel [PMID:25017478], or (4) strand bias was recorded.

Samples that failed to amplify due to insufficient DNA: 02, 09,10, and 1[illegible]

Sample	Volume Change/ year	Pathology	Sample Notes	Tumor Cellularity Estimate	Chromosome	Position	Ref	Variant	Allelic Frequency	Adjusted Allelic Frequency	Quality	Original Coverage	Allele Coverage (# allele reads)	Allele.Name	Gene.ID	Codon	Variant Call	Consequence and Notes
PF239	-39%	Hyperplastic		13%	chr2	212812097	T	C	45%	344%	405.69	94	42	---	ERBB4	?	SNP	Intronic variant (33%)
				13%	chr4	1807894	G	A	100%	769%	8216.08	508	518	---	FGFR3	651	SNP	Synonymous variant (4%)
				13%	chr4	55141055	A	G	100%	769%	4454.32	248	251	---	PDGFRA	567	SNP	Synonymous variant (4%)
				13%	chr4	55152040	C	T	53%	407%	4401.12	661	351	COSM22413	PDGFRA	824	Silent	p.V824V
				13%	chr4	55946354	G	T	50%	381%	3700.63	738	366	---	KDR	?	SNP	Intronic variant
				13%	chr4	55972974	T	A	48%	368%	1650.62	355	170	---	KDR	472	Missense	Missense (Q>H) - Tolerated
				13%	chr4	55980239	C	T	100%	769%	2224.42	140	140	---	KDR	?	SNP	Intronic variant (48%)
				13%	chr5	112175770	G	A	100%	769%	7220.15	404	408	---	APC	1493	SNP	Synonymous variant
				13%	chr7	55249063	G	A	100%	769%	2320.52	152	152	---	EGFR	?	SNP	Intronic variant (42%)
				13%	chr7	140453136	A	T	7%	52%	105.26	425	29		BRAF	600	Missense	Missense (V>E) - Pathogenic
				13%	chr10	43613843	G	T	100%	769%	8290.31	523	523	---	RET	769	SNP	Synonymous variant (28%)
				13%	chr11	534242	A	G	49%	376%	3834.62	648	318	COSM249860	HRAS	27	Silent	p.H27H
				13%	chr13	28610183	A	G	54%	415%	8155.84	1429	771	---	FLT3	?	SNP	Splice region variant (38%)
				13%	chr17	7579472	G	C	100%	769%	4163.95	223	281	---	TP53	72	Missense	Missense (P/R) - Tolerated
				13%	chr18	48586344	C	T	48%	372%	3595.95	743	361	---	SMAD4	?	SNP	Intronic variant (5%)
				13%	chr19	1220321	T	C	98%	752%	4833.4	316	314	---	STK11	?	SNP	Intronic variant (35%)
PF241	14%	Hyperplastic		60%	chr10	43613843	G	T	100%	167%	130.46	12	12	---	RET	769	SNP	Synonymous variant (28%)
				60%	chr11	534242	A	G	75%	125%	76.4	12	9	COSM249860	HRAS	27	Silent	p.H27H
				60%	chr12	25398284	C	A	47%	79.00%	70.25	18	9	---	KRAS	12	Missense	Missense variant, (G>D) codon 12, predicted to be deleterious
				60%	chr12	25398284	C	A	45%	75%	68.2	21	9	COSM520	KRAS	12	Missense	p.G12V
				60%	chr13	28610183	A	G	72%	120%	307.55	36	26	---	FLT3	?	SNP	Splice region variant (38%)
PF245	67%	ubular Adenon		40%	chr2	209113192	G	A	53%	131%	12169	1839	969	NOCOSMIC105	IDH1	105	SNP	Synonymous variant (5%)
				40%	chr2	212812097	T	C	48%	121%	2123.47	439	213	---	ERBB4	?	SNP	Intronic variant (33%)
				40%	chr3	178917005	A	G	53%	132%	2581.01	471	249	---	PIK3CA	?	SNP	Downstream gene variant (22%)
				40%	chr3	178952020	C	T	50%	126%	5561.1	907	457	COSM21451	PIK3CA	1025	Silent	p.T1025T
				40%	chr4	1807894	G	A	100%	250%	6159.46	386	387	---	FGFR3	651	SNP	Synonymous variant (4%)
				40%	chr4	55141055	A	G	100%	250%	7520.23	423	424	---	PDGFRA	567	SNP	Synonymous variant (4%)
				40%	chr4	55980239	C	T	32%	80%	920.36	373	119	---	KDR	?	SNP	Intronic variant (49%)
				40%	chr5	112175212	AAAAG	-	35%	89%	1223.37	338	122	---	APC	1307	Missense	Missense pathogenic, truncation
				40%	chr5	112175212	AAAAG	-	35%	88%	1245.53	348	122	COSM18764	APC	1309	Frameshift Del	p.E1309fs*4
				40%	chr5	112175213	AAAGA	-	35%	88%	1245.53	348	122	COSM19263	APC	1309	Frameshift Del	p.E1309fs*4
				40%	chr5	112175216	GAAAA	-	35%	88%	1245.53	348	122	COSM19145	APC	1309	Frameshift Del	p.E1309fs*4
				40%	chr5	112175217	AAAAG	-	35%	88%	1245.53	348	122	COSM18701	APC	1309	Frameshift Del	p.E1309fs*4
				40%	chr5	112175218	AAAGA	-	35%	88%	1245.53	348	122	COSM13113	APC	1309	Frameshift Del	p.E1309fs*4
				40%	chr5	112175770	G	A	100%	250%	15659.5	865	864	---	APC	1493	SNP	Synonymous
				40%	chr5	149433596	TG	GA	100%	250%	3453.4	220	220	---	CSF1R	?	SNP	Downstream gene variant
				40%	chr7	55249063	G	A	33%	83%	810.85	308	103	---	EGFR	?	SNP	Intronic variant (42%)
				40%	chr10	43613843	G	T	51%	128%	2619.93	503	258	---	RET	769	SNP	Synonymous variant (28%)
				40%	chr10	43615633	C	G	58%	146%	2457.7	381	223	---	RET	904	SNP	Synonymous variant (16%)
				40%	chr13	28602292	T	C	49%	123%	5917.92	1196	588	---	FLT3	?	SNP	Intronic variant (4%)
				40%	chr13	28610183	A	G	100%	250%	22911.5	1430	1432	---	FLT3	?	SNP	Splice region variant (38%)
				40%	chr13	49033829	C	T	37%	92%	1252.51	394	146	---	RB1	656	Missense	p.R656W SIFT = deleterious, PolyPhen = benign
				40%	chr17	7579472	G	C	46%	115%	2137.26	281	180	---	TP53	72	Missense	Missense (P/R) - Tolerated
				40%	chr22	24176287	G	A	52%	131%	2449.35	375	196	COSM1090	SMARCB1	?	Intronic Substitution	p.?
PF249	-29%	ubular Adenon		30%	chr3	178917005	A	G	46%	154%	1476.11	331	153	---	PIK3CA	?	SNP	Downstream gene variant (22%)
				30%	chr4	1807894	G	A	100%	333%	4912.24	313	313	---	FGFR3	651	SNP	Synonymous variant (4%)
				30%	chr4	55141055	A	G	100%	333%	6988.38	396	396	---	PDGFRA	567	SNP	Synonymous variant (4%)
				30%	chr4	55152040	C	T	52%	173%	4104.5	639	332	COSM22413	PDGFRA	824	Silent	p.V824V
				30%	chr4	55946171	G	A	58%	193%	1009.23	164	95	---	KDR	1336	SNP	Synonymous variant (1.5%)
				30%	chr4	55962546	-	G	45%	148%	1535.56	763	245	---	KDR	?	Insertion	Noncoding region, unknown
				30%	chr4	55972974	T	A	47%	156%	2936.82	640	300	---	KDR	472	Missense	Missense (Q>H) - Tolerated
				30%	chr4	55980239	C	T	34%	114%	1006.06	365	125	---	KDR	?	SNP	Intronic variant (48%)
				30%	chr5	112175212	AAAAG	-	57%	189%	2156.23	280	165	---	APC	1307	Missense	Truncation
				30%	chr5	112175212	AAAAG	-	57%	188%	2161.97	297	165	COSM18764	APC	1309	Frameshift Del	p.E1309fs*4
				30%	chr5	112175213	AAAGA	-	57%	188%	2161.97	297	165	COSM19263	APC	1309	Frameshift Del	p.E1309fs*4

				30%	chr5	112175216	GAAAA	-	57%	188%	2161.97	297	165	COSM19145	APC	1309	Frameshift Del	p.E1309fs*4
				30%	chr5	112175217	AAAAG	-	57%	188%	2161.97	297	165	COSM18701	APC	1309	Frameshift Del	p.E1309fs*4
				30%	chr5	112175218	AAAGA	-	57%	188%	2161.97	297	165	COSM13113	APC	1309	Frameshift Del	p.E1309fs*4
				30%	chr5	112175770	G	A	25%	83%	1830.23	851	212	---	APC	1493	SNP	Synonymous
				30%	chr5	149433596	TG	GA	100%	333%	3101.87	194	194	---	CSF1R	?	SNP	Downstream gene variant
				30%	chr7	116339672	C	T	52%	172%	6190.73	1163	602	---	MET	178	Silent	p.S178S COSM1579024
				30%	chr11	534242	A	G	53%	177%	2705.85	395	212	COSM249860	HRAS	27	Silent	p.H27H
				30%	chr13	28610183	A	G	61%	202%	10527.5	1533	930	---	FLT3	?	SNP	Splice region variant (38%)
				30%	chr17	7579472	G	C	90%	301%	5883.46	389	352	---	TP53	72	Missense	Missense (P/R) - Tolerated
PF250	147%	Tubulovillous		70%	chr3	178917005	A	G	44%	63%	1017.82	251	110	---	PIK3CA	?	SNP	Downstream gene variant (22%)
				70%	chr4	1807894	G	A	100%	143%	4205.37	268	269	---	FGFR3	651	SNP	Synonymous variant (4%)
				70%	chr4	55141055	A	G	100%	143%	5244.73	297	299	---	PDGFRA	567	SNP	Synonymous variant (4%)
				70%	chr4	55152040	C	T	53%	76%	4036.17	595	317	COSM22413	PDGFRA	824	Silent	p.V824V
				70%	chr4	55946171	G	A	49%	70%	1098.26	225	111	---	KDR	1336	SNP	Synonymous variant (1.5%)
				70%	chr4	55962546	-	G	45%	64%	1245.77	646	200	---	KDR	?	Insertion	Noncoding region, unknown
				70%	chr4	55972974	T	A	50%	71%	2511.92	502	251	---	KDR	472	Missense	Missense (Q>H) - Tolerated
				70%	chr5	112175427	-	AGAC	79%	113%	6327.07	525	417	---	APC	1379	Insertion	p.E1379ETX effect unknown
				70%	chr5	112175770	G	A	92%	131%	8939.38	575	530	---	APC	1493	SNP	Synonymous
				70%	chr5	149433596	TG	GA	100%	143%	2223.45	140	140	---	CSF1R	?	SNP	Downstream gene variant
				70%	chr7	116339672	C	T	37%	52%	3939.09	1266	466	---	MET	178	Silent	p.S178S COSM1579024
				70%	chr11	534242	A	G	42%	60%	1437.68	313	132	COSM249860	HRAS	27	Silent	p.H27H
				70%	chr12	25398281	C	T	24%	34%	1807.87	867	212	---	KRAS	13	missense	p.G13D COSM532 pathogenic
				70%	chr12	25398281	C	T	24%	34%	1808.7	886	212	COSM532	KRAS	13	Missense	p.G13D pathogenic
				70%	chr13	28610183	A	G	55%	78%	5833.44	996	545	---	FLT3	?	SNP	Splice region variant (38%)
				70%	chr17	7579472	G	C	100%	143%	5550.38	305	310	---	TP53	72	Missense	Missense (P/R) - Tolerated
PF252	-27%	ıbular Adenon		20%	chr4	1807894	G	A	100%	500%	101.91	10	10	---	FGFR3	769	SNP	Synonymous variant (28%)
				20%	chr4	55961036	A	G	49%	243%	2206.95	461	224	---	KDR	968	SNP	p.S968S
				20%	chr11	534242	A	G	67%	334%	85.04	15	10	COSM249860	HRAS	27	Silent	p.H27H
				20%	chr13	28602292	T	C	52%	261%	102.51	22	12	---	FLT3	?	SNP	Intronic variant (4%)
				20%	chr13	28610183	A	G	91%	455%	403.14	33	30	---	FLT3	?	SNP	Splice region variant (38%)
PF254	21%	ıbular Adenon		15%	chr2	209113154	G	A	7%	45%	64.78	480	32	---	IDH1	118	Missense	p.P118L, conflicting predicted results
				15%	chr4	1807871	A	G	7%	45%	104.4	774	53	---	FGFR3	532/644	Missense	p.N532/644D
				15%	chr4	1807894	G	A	100%	667%	13537.5	843	852	---	FGFR3	651	SNP	Synonymous variant (4%)
				15%	chr4	55141055	A	G	100%	667%	1785.55	108	108	---	PDGFRA	567	SNP	Synonymous variant (4%)
				15%	chr4	55593481	A	G	52%	343%	5682.34	881	455	COSM21983	KIT	546	Silent	p.K546K
				15%	chr4	55980239	C	T	26%	175%	154.21	84	22	---	KDR	?	SNP	Intronic variant (48%)
				15%	chr4	153247358	T	C	6%	38%	152.18	2222	114	---	FBXW7	482	Missense	p.T482A, deleterious by both SIFT and PolyPhen
				15%	chr5	112175770	G	A	57%	379%	842.34	123	70	---	APC	1493	SNP	Synonymous variant
				15%	chr5	149433596	TG	GA	99%	661%	2065.07	133	132	---	CSF1R	?	SNP	Downstream gene variant
				15%	chr7	55221893	C	T	9%	57%	58.05	221	19	---	EGFR			intronic variant, rs374526327
				15%	chr7	55249063	G	A	99%	659%	1381.47	88	89	---	EGFR	?	SNP	Intronic variant (42%)
				15%	chr7	55249174	A	G	6%	39%	33.24	289	17	---	EGFR		SNP	intronic variant rs191420937
				15%	chr7	116339673	G	A	5%	36%	126.48	3031	107	---	MET	179	Missense	p.A179T, predicted to be benign
				15%	chr9	139399341	T	A	7%	48%	39.9	208	15	---	NOTCH1	1601	Missense	p.H1601L, predicted to be benign
				15%	chr10	43613843	G	T	100%	667%	7372.27	463	466	---	RET	769	SNP	Synonymous variant (28%)
				15%	chr11	534242	A	G	52%	347%	8995.17	1374	717	COSM249860	HRAS	27	Silent	p.H27H
				15%	chr16	68835637	A	G	8%	51%	328.8	4201	153	---	CDH1	76	Silent	p.K76K
				15%	chr17	7579419	AG	GA	17%	116%	42.31	59	12	---	TP53	90	frameshift	p.S?90F?
				15%	chr17	7579472	G	C	91%	609%	1407.89	90	84	---	TP53	72	Missense	Missense (P/R) - Tolerated
				15%	chr19	1220321	T	C	52%	345%	562.11	111	58	---	STK11	?	SNP	Intronic variant (35%)
PF255	17%	Hyperplastic		25%	chr4	1807894	G	A	100%	400%	31897.3	5770	1993	---	FGFR3	651	SNP	Synonymous variant (4%)
				25%	chr4	55141055	A	G	100%	400%	33989.7	1847	1870	---	PDGFRA	567	SNP	Synonymous variant (4%)
				25%	chr4	55593481	A	G	52%	209%	13118.9	10140	1042	COSM21983	KIT	546	Silent	p.K546K
				25%	chr4	55980239	C	T	39%	156%	3711.04	1083	422	---	KDR	?	SNP	Intronic variant (48%)
				25%	chr5	112175770	G	A	42%	170%	9648.67	4103	847	---	APC	1493	SNP	Synonymous, codon 1493
				25%	chr5	149433596	TG	GA	100%	400%	22300.8	1393	1394	---	CSF1R	?	SNP	Downstream gene variant
				25%	chr7	55249063	G	A	100%	400%	24983.7	1570	1569	---	EGFR	?	SNP	Intronic variant (42%)
				25%	chr7	140453136	A	T	33%	131%	6550.8	2732	653	COSM476	BRAF	600	Missense	p.V600E
				25%	chr9	139390805	G	-	100%	400%	32000.6	1992	1848	COSM13070	NOTCH1	2463	Frameshift Del	p.A2463fs*14
				25%	chr10	43613843	G	T	100%	400%	31999.7	10787	1995	---	RET	769	SNP	Synonymous variant (28%)

				25%	chr11	534242	A	G	58%	234%	15505.3	7077	1167	COSM249860	HRAS	27	Silent	p.H27H
				25%	chr17	7579472	G	C	100%	400%	30827	1769	1741	---	TP53	72	Missense	Missense (P/R) - Tolerated
				25%	chr19	1220321	T	C	57%	229%	12462	2336	1137	---	STK11	?	SNP	Intronic variant (35%)
PF256	95%	Tubulovillous		50%	chr2	209113192	G	A	52%	105%	5245.68	793	416	NOCOSMIC105	IDH1	105	SNP	Synonymous variant (5%)
				50%	chr4	1807894	G	A	100%	200%	3262.55	212	211	---	FGFR3	769	SNP	Synonymous variant (28%)
				50%	chr4	55141013	G	A	39%	77%	824.97	194	75	---	PDGFRA	553	Silent	p.P553P
				50%	chr4	55141055	A	G	100%	200%	3416.28	187	188	---	PDGFRA	567	SNP	Synonymous variant (4%)
				50%	chr4	153249385	G	A	29%	57%	942.44	351	100	---	FBXW7	289/**465**	Missense	R>C, codon not well annotated
				50%	chr4	153249385	G	A	29%	57%	918.12	351	100	COSM170725	FBXW7	385	Missense	p.R385C
				50%	chr4	153249385	G	A	29%	57%	918.12	351	100	COSM170726	FBXW7	226	Missense	p.R226C
				50%	chr4	153249385	G	A	29%	57%	918.12	351	100	COSM170727	FBXW7	465	Missense	p.R465C
				50%	chr4	153249385	G	A	29%	57%	918.12	351	100	COSM22932	FBXW7	465	Missense	p.R465C
				50%	chr5	112175617	T	A	46%	92%	1298.24	245	113	COSM19049	APC	1442	Silent	p.P1442P
				50%	chr5	112175770	G	A	100%	200%	7429.04	420	419	---	APC	1493	SNP	Synonymous variant (34%)
				50%	chr5	149433596	TG	GA	31%	62%	364.77	159	49	---	CSF1R	?	SNP	Downstream gene variant
				50%	chr7	55249063	G	A	100%	200%	2809.21	179	179	---	EGFR	?	SNP	Intronic variant (42%)
				50%	chr10	43613843	G	T	100%	200%	3766.15	237	237	---	RET	769	SNP	Synonymous variant (28%)
				50%	chr10	43615633	C	G	63%	126%	1286.41	177	113	---	RET	904	SNP	Synonymous variant (16%)
				50%	chr11	534242	A	G	43%	85%	1325.79	273	117	COSM249860	HRAS	27	Silent	p.H27H
				50%	chr12	25380276	T	A	54%	108%	6126.81	883	479	COSM553	KRAS	61	Missense	p.Q61L
				50%	chr13	28610183	A	G	46%	92%	3056.37	688	316	---	FLT3	?	SNP	Splice region variant (38%)
				50%	chr17	7579472	G	C	99%	197%	4022.71	235	232	---	TP53	72	Missense	Missense (P/R) - Tolerated
				50%	chr19	1220321	T	C	43%	86%	426.51	110	48	---	STK11	?	SNP	Intronic variant (35%)
PF259	3%	ubular Adenon		38%	chr2	212812097	T	C	49%	131%	1226.54	246	121	---	ERBB4	?	SNP	Intronic variant (33%)
				38%	chr4	1807894	G	A	100%	265%	3116.83	197	196	---	FGFR3	651	SNP	Synonymous variant (4%)
				38%	chr4	55141055	A	G	100%	267%	4480.21	252	252	---	PDGFRA	567	SNP	Synonymous variant (4%)
				38%	chr4	55152040	C	T	53%	141%	2742.5	420	223	COSM22413	PDGFRA	824	Silent	p.V824V
				38%	chr4	55962546	-	G	43%	114%	968.46	511	161	---	KDR	?	Insertion	Noncoding region, unknown
				38%	chr4	55972974	T	A	51%	135%	2385.76	465	236	---	KDR	472	Missense	Missense (Q>H) - Tolerated
				38%	chr4	55980239	C	T	100%	267%	2351.96	154	154	---	KDR	?	SNP	Intronic variant (48%)
				38%	chr5	112175212	AAAAG	-	77%	205%	2070.29	174	139	---	APC	1307	Missense	Truncation, missense pathogenic
				38%	chr5	112175212	AAAAG	-	77%	205%	2089.86	183	139	COSM18764	APC	1309	Frameshift Del	p.E1309fs*4
				38%	chr5	112175213	AAAGA	-	77%	205%	2089.86	183	139	COSM19263	APC	1309	Frameshift Del	p.E1309fs*4
				38%	chr5	112175216	GAAAA	-	77%	205%	2089.86	183	139	COSM19145	APC	1309	Frameshift Del	p.E1309fs*4
				38%	chr5	112175217	AAAAG	-	77%	205%	2089.86	183	139	COSM18701	APC	1309	Frameshift Del	p.E1309fs*4
				38%	chr5	112175218	AAAGA	-	77%	205%	2089.86	183	139	COSM13113	APC	1309	Frameshift Del	p.E1309fs*4
				38%	chr5	112175770	G	A	84%	224%	7483.31	562	471	---	APC	1493	SNP	Synonymous, codon 1493
				38%	chr5	149433596	TG	GA	99%	264%	1751.72	117	116	---	CSF1R	?	SNP	Downstream gene variant
				38%	chr7	55249063	G	A	56%	148%	884.89	152	86	---	EGFR	?	SNP	Intronic variant (42%)
				38%	chr10	43613843	G	T	100%	267%	4771.16	305	306	---	RET	769	SNP	Synonymous variant (28%)
				38%	chr10	43615633	C	G	47%	126%	1024.69	224	106	---	RET	904	SNP	Synonymous variant (16%)
				38%	chr11	534242	A	G	100%	267%	4275.45	242	243	COSM249860	HRAS	27	Silent	p.H27H
				38%	chr13	28610183	A	G	47%	125%	2975.9	656	308	---	FLT3	?	SNP	Splice region variant (38%)
				38%	chr17	7577022	G	A	12%	31%	122.1	180	21	COSM10663	TP53	306	Nonsense	p.R306*
				38%	chr17	7577022	G	A	12%	31%	122.1	180	21	COSM99947	TP53	306	Nonsense	p.R306*
				38%	chr19	1220321	T	C	53%	141%	1032.7	187	99	---	STK11	?	SNP	Intronic variant (35%)
				38%	chr22	24176287	G	A	53%	141%	1859.42	279	148	COSM1090	SMARCB1	?	Intronic Substitution	p.?
264	-4%	ubular Adenon		25%	chr4	1807894	G	A	100%	400%	10396.1	648	648	---	FGFR3	651	SNP	Synonymous variant (4%)
				25%	chr4	55141055	A	G	99%	396%	14055.9	795	790	---	PDGFRA	567	SNP	Synonymous variant (4%)
				25%	chr4	55962546	-	G	100%	400%	13268.7	1994	1072	---	KDR	?	Insertion	Noncoding region, unknown
				25%	chr4	55972974	T	A	100%	400%	26591.9	1665	1666	---	KDR	472	Missense	Missense (Q>H) - Tolerated
				25%	chr4	55980239	C	T	99%	395%	8308.04	536	529	---	KDR	?	SNP	Intronic variant (48%)
				25%	chr5	112175576	C	T	11%	46%	554.43	906	103	COSM18836	APC	1429	Nonsense	p.Q1429*
				25%	chr5	112175639	C	T	10%	41%	460.93	911	93	COSM13127	APC	1450	Nonsense	p.R1450*
				25%	chr5	112175770	G	A	100%	400%	33650.7	1857	1853	---	APC	1493	SNP	Synonymous
				25%	chr7	55249063	G	A	99%	395%	7745.81	496	491	---	EGFR	?	SNP	Intronic variant (42%)
				25%	chr10	43613843	G	T	100%	400%	16640.8	1038	1039	---	RET	769	SNP	Synonymous variant (28%)
				25%	chr10	43615633	C	G	50%	202%	3668.52	715	362	---	RET	904	SNP	Synonymous variant (16%)
				25%	chr11	534242	A	G	99%	396%	17690.1	983	981	COSM249860	HRAS	27	Silent	p.H27H
				25%	chr13	28602292	T	C	52%	208%	10803.6	2226	1039	---	FLT3	?	SNP	Intronic variant (4%)
				25%	chr13	28610183	A	G	100%	400%	31810.3	2602	1993	---	FLT3	?	SNP	Splice region variant (38%)

				25%	chr17	7579472	G	C	57%	228%	5031.83	622	411	---	TP53	72	Missense	Missense (P/R) - Tolerated
				25%	chr19	1220321	T	C	57%	226%	2990.46	482	275	---	STK11	?	SNP	Intronic variant (35%)
				25%	chr19	17945696	C	T	50%	199%	4324.12	722	359	COSM34213	JAK3	722	Missense	p.V722I
PF270	24%	Tubular Adenoma with High Grade Dysplasia		45%	chr4	1807894	G	A	100%	222%	160.32	14	14	---	FGFR3	651	SNP	Synonymous variant (4%)
				45%	chr4	153249384	C	T	46%	103%	99.53	26	12	COSM117308	FBXW7	385	Missense	p.R385H
				45%	chr4	153249384	C	T	46%	103%	99.53	26	12	COSM117309	FBXW7	226	Missense	p.R226H
				45%	chr4	153249384	C	T	46%	103%	99.53	26	12	COSM117310	FBXW7	465	Missense	p.R465H
				45%	chr4	153249384	C	T	46%	103%	99.53	26	12	COSM22965	FBXW7	465	Missense	p.R465H
				45%	chr7	116339672	C	T	76%	168%	459.92	53	40	---	MET	178	Silent	p.S178S COSM1579024
				45%	chr9	139390805	G	-	63%	139%	77.89	16	10	COSM13070	NOTCH1	2463	Frameshift Del	p.A2463fs*14
				45%	chr11	534242	A	G	71%	159%	149.75	21	15	COSM249860	HRAS	27	Silent	p.H27H
				45%	chr17	7578265	A	G	91%	201%	225.9	20	19	---	TP53	195	Missense	p.I195T, predicted to be deleterious
				45%	chr17	7578265	A	G	87%	193%	234.7	23	20	COSM11089	TP53	195	Missense	p.I195T
				45%	chr17	7579472	G	C	100%	222%	217.33	17	18	---	TP53	72	Missense	Missense (P/R) - Tolerated
PF273	43%	ubular Adenon		10%	chr2	212812097	T	C	62%	618%	3852.45	551	341	---	ERBB4	?	SNP	Intronic variant (33%)
				10%	chr4	1807894	G	A	100%	1000%	10586.2	666	666	---	FGFR3	651	SNP	Synonymous variant (4%)
				10%	chr4	55141055	A	G	100%	1000%	21415.6	1161	1189	---	PDGFRA	567	SNP	Synonymous variant (4%)
				10%	chr4	55593464	A	C	100%	1000%	36234.8	2657	1990	COSM28026	KIT	541	Missense	p.M541L
				10%	chr5	112175656	-	A	8%	76%	93.57	1024	77	COSM19119	APC	1455	Frameshift Ins	p.N1455fs*2
				10%	chr5	112175770	G	A	60%	601%	14447.9	1773	1087	---	APC	1493	SNP	Synonymous
				10%	chr5	149433596	TG	GA	100%	1000%	6940.08	446	444	---	CSF1R	?	SNP	Downstream gene variant
				10%	chr7	55249063	G	A	100%	1000%	10251	644	650	---	EGFR	?	SNP	Intronic variant (42%)
				10%	chr9	139390805	G	-	100%	1000%	7211.05	542	553	COSM13070	NOTCH1	2463	Frameshift Del	p.A2463fs*14
				10%	chr10	43613843	G	T	100%	1000%	21282.4	1331	1332	---	RET	769	SNP	Synonymous variant (28%)
				10%	chr11	534242	A	G	49%	492%	6554.39	1080	536	COSM249860	HRAS	27	Silent	p.H27H
				10%	chr13	28610183	A	G	100%	1000%	31867.2	3590	1995	---	FLT3	?	SNP	Splice region variant (38%)
				10%	chr17	7579472	G	C	100%	1000%	11863.8	632	687	---	TP53	72	Missense	Missense (P/R) - Tolerated
				10%	chr22	24134064	C	A	9%	87%	348.71	1567	114	---	SMARCB1	72	Missense	p.T72K - tolerated
PF274	-40%	ubular Adenon		30%	chr2	212812097	T	C	51%	170%	7217.89	1375	704	---	ERBB4	?	SNP	Intronic variant (33%)
				30%	chr4	1807894	G	A	100%	333%	10035.8	634	633	---	FGFR3	651	SNP	Synonymous variant (4%)
				30%	chr4	55141055	A	G	100%	333%	24646.7	1328	1374	---	PDGFRA	567	SNP	Synonymous variant (4%)
				30%	chr4	55593464	A	C	100%	333%	36272.8	2594	1991	COSM28026	KIT	541	Missense	p.M541L
				30%	chr5	112175656	-	A	8%	28%	112.78	1023	85	COSM19119	APC	1455	Frameshift Ins	p.N1455fs*2
				30%	chr5	112175770	G	A	79%	263%	24036.3	2351	1564	---	APC	1493	SNP	Synonymous
				30%	chr5	149433596	TG	GA	100%	333%	8089.48	510	510	---	CSF1R	?	SNP	3' UTR variant
				30%	chr7	55249063	G	A	100%	333%	9536.9	600	604	---	EGFR	?	SNP	Protein coding, synonymous variant
				30%	chr9	139390805	G	-	100%	333%	6123.92	494	501	COSM13070	NOTCH1	2463	Frameshift Del	p.A2463fs*14
				30%	chr10	43613843	G	T	100%	333%	21578.4	1347	1355	---	RET	769	SNP	Synonymous variant
				30%	chr11	534242	A	G	50%	167%	6138.89	1000	505	COSM249860	HRAS	27	Silent	p.H27H
				30%	chr13	28610183	A	G	100%	333%	31831.1	3426	1993	---	FLT3	?	SNP	Splice region variant (38%)
				30%	chr17	7579472	G	C	100%	333%	12491	656	752	---	TP53	72	Missense	Variant (P>R), not pathogenic
				30%	chr22	24134064	C	A	13%	42%	726.75	1700	178	---	SMARCB1	72	Missense	p.T72K - tolerated
276	-23%	Sessile Serrated Adenoma		60%	chr2	209113154	G	A	6%	11%	209.71	2322	128	---	IDH1	118	Missense	p.P118L, conflicting predicted results
				60%	chr4	1807871	A	G	7%	11%	219	4937	130	---	FGFR3	532/644	Missense	p.N532/644D
				60%	chr4	1807894	G	A	100%	167%	31768.9	5357	1989	---	FGFR3	651	SNP	Synonymous variant (4%)
				60%	chr4	55141055	A	G	100%	167%	17489	949	964	---	PDGFRA	567	SNP	Synonymous variant (4%)
				60%	chr4	55980239	C	T	33%	55%	1422.68	542	178	---	KDR	?	SNP	Intronic variant (48%)
				60%	chr4	153247358	T	C	6%	10%	179.96	10472	121	---	FBXW7	482	Missense	p.T482A, deleterious by both SIFT and PolyPhen
				60%	chr5	112175589	C	T	6%	10%	98.32	1151	67	---	APC	1433	Missense	Missense (P>L) Deleterious
				60%	chr5	112175770	G	A	100%	167%	16259.6	888	893	---	APC	1493	SNP	Synonymous
				60%	chr5	149433596	TG	GA	28%	47%	3227.05	1591	449	---	CSF1R	?	SNP	Downstream gene variant
				60%	chr7	55249174	A	G	7%	11%	211	2813	127	---	EGFR		SNP	intronic variant rs191420937
				60%	chr7	116339673	G	A	6%	10%	156.32	12767	115	---	MET	179	Missense	p.A179T, predicted to be benign
				60%	chr9	139399341	T	A	5%	8%	89.53	1695	85	---	NOTCH1	1601	Missense	p.H1601L, predicted to be benign
				60%	chr10	43613843	G	T	48%	80%	9557.24	4355	961	---	RET	769	SNP	Synonymous variant (28%)
				60%	chr11	534242	A	G	52%	87%	13226.9	6506	1048	COSM249860	HRAS	27	Silent	p.H27H
				60%	chr11	108236261	A	C	5%	8%	78.27	1679	81	---	ATM			3' UTR variant
				60%	chr13	28602292	T	C	49%	82%	9961.92	6462	987	---	FLT3	?	SNP	Intronic variant (4%)
				60%	chr13	28610183	A	G	100%	167%	31925.2	9350	1996	---	FLT3	?	SNP	Splice region variant (38%)
				60%	chr16	68835637	A	G	6%	10%	167.83	19974	118	---	CDH1	76	Silent	p.K76K

				60%	chr17	7579472	G	C	93%	155%	11383.1	721	675	---	TP53	72	Missense	Missense (P/R) - Tolerated
				60%	chr22	24176287	G	A	51%	84%	7210.71	1149	585	COSM1090	SMARCB1	?	Intronic Substitution	p.?
277	66%	ubular Adenon		13%	chr4	1807894	G	A	100%	800%	26155.1	1639	1642	---	FGFR3	651	SNP	Synonymous variant (4%)
				13%	chr4	55141055	A	G	100%	800%	35514.9	2229	1954	---	PDGFRA	567	SNP	Synonymous variant (4%)
				13%	chr4	55980239	C	T	35%	278%	5467.34	1917	668	---	KDR	?	SNP	Intronic variant (48%)
				13%	chr5	112175770	G	A	100%	800%	36047	4402	1980	---	APC	1439	SNP	Synonymous
				13%	chr5	149433596	TG	GA	27%	214%	2680.54	1443	387	---	CSF1R	?	SNP	Downstream gene variant
				13%	chr10	43613843	G	T	52%	414%	10711.8	2556	1033	---	RET	769	SNP	Synonymous variant (28%)
				13%	chr11	534242	A	G	48%	385%	9884.31	1702	824	COSM249860	HRAS	27	Silent	p.H27H
				13%	chr13	28602292	T	C	49%	388%	9670.64	4772	969	---	FLT3	?	SNP	Intronic variant (4%)
				13%	chr13	28610183	A	G	100%	800%	31963.7	5395	1997	---	FLT3	?	SNP	Splice region variant (38%)
				13%	chr17	7579472	G	C	100%	800%	31383.8	1695	1762	---	TP53	72	Missense	Missense (P/R) - Tolerated
				13%	chr22	24176287	G	A	48%	386%	11676.1	2473	964	COSM1090	SMARCB1	?	Intronic Substitution	p.?
PF278	-49%	ubular Adenon		13%	chr2	209113192	G	A	99%	790%	35503.2	5640	1973	NOCOSMIC105	IDH1	105	SNP	Synonymous variant (5%)
				13%	chr4	1807894	G	A	100%	800%	18303.3	1150	1152	---	FGFR3	?	SNP	Protein coding, synonymous variant
				13%	chr4	55141055	A	G	100%	800%	19040.7	1051	1073	---	PDGFRA	?	SNP	Protein coding, synonymous variant
				13%	chr4	55593481	A	G	50%	398%	12269.4	3703	996	COSM21983	KIT	546	Silent	p.K546K
				13%	chr4	55980239	C	T	22%	172%	1101.5	865	185	---	KDR	?	SNP	Intronic variant
				13%	chr5	112175379	AAAGT	-	12%	93%	254.49	2363	227	---	APC	1363	Deletion	p.KS?1363? Out of frame deletion, effect unknown
				13%	chr5	112175656	-	A	12%	93%	227.9	1286	149	COSM19119	APC	1455	Frameshift Ins	p.N1455fs*2
				13%	chr5	112175770	G	A	50%	398%	12025.5	2123	981	---	APC	1493	SNP	Synonymous variant (34%)
				13%	chr5	149433596	TG	GA	99%	791%	8319.84	530	528	---	CSF1R	?	SNP	Downstream gene variant (27%)
				13%	chr7	55249063	G	A	44%	350%	2932.14	711	314	---	EGFR	742	SNP	Synonymous variant (42%)
				13%	chr9	139390805	G	-	100%	800%	8916.63	695	702	COSM13070	NOTCH1	2463	Frameshift Del	p.A2463fs*14
				13%	chr10	43613843	G	T	51%	406%	7474.56	1437	734	---	RET	769	SNP	Synonymous variant (28%)
				13%	chr13	28602292	T	C	51%	406%	10408.3	4001	1015	---	FLT3	?	SNP	Intronic variant (4%)
				13%	chr13	28610183	A	G	54%	433%	11494.8	4636	1081	---	FLT3	?	SNP	Splice region variant (38%)
				13%	chr17	7579472	G	C	89%	715%	13220	904	816	---	TP53	72	Missense	Missense (P/R) - Tolerated
				13%	chr17	7579473	G	C	9%	74%	393.36	908	84	---	TP53	72	Missense	Missense (P/A) - Tolerated
				13%	chr18	48586344	C	T	56%	446%	12018.6	3954	1113	---	SMAD4	?	SNP	Intronic variant (5%)
				13%	chr19	1220321	T	C	51%	407%	4745.58	865	469	---	STK11	?	SNP	Intronic variant (35%)
				13%	chr22	24134064	C	A	13%	102%	521.6	1205	119	---	SMARCB1	72	Missense	p.T72K - tolerated
PF279	-22%	Hyperplastic		60%	chr4	1807894	G	A	100%	167%	29229.6	1832	1830	---	FGFR3	651	SNP	Synonymous variant (4%)
				60%	chr4	55141055	A	G	100%	167%	30353.4	1663	1676	---	PDGFRA	567	SNP	Synonymous variant (4%)
				60%	chr4	55152040	C	T	98%	163%	34730.9	2752	1953	COSM22413	PDGFRA	824	Silent	p.V824V
				60%	chr4	55980239	C	T	35%	59%	4779.03	1657	583	---	KDR	?	SNP	Intronic variant (48%)
				60%	chr5	112175643	-	CA	13%	22%	1428.91	1955	257	---	APC	1451	insertion	p.E1451A?, probably pathogenic?
				60%	chr5	112175770	G	A	52%	87%	13071.7	4431	1040	---	APC	1493	SNP	Synonymous
				60%	chr5	149433596	TG	GA	26%	43%	2847.26	1623	419	---	CSF1R	?	SNP	3' UTR variant
				60%	chr5	170837514	-	T	97%	161%	470.04	2444	1795	---	NPM1		insertion	intronic variant
				60%	chr7	55249063	G	A	51%	85%	6874.72	1312	672	---	EGFR	?	SNP	Protein coding, synonymous variant
				60%	chr7	116340269	C	T	49%	81%	9695.65	2172	970	---	MET	377	Silent	p.I377I
				60%	chr10	43613843	G	T	100%	167%	31528.7	2332	1984	---	RET	769	SNP	Synonymous variant (28%)
				60%	chr11	534242	A	G	98%	164%	35239.8	2232	1968	COSM249860	HRAS	27	Silent	p.H27H
				60%	chr13	28610183	A	G	51%	85%	10488.3	5661	1020	---	FLT3	?	SNP	Splice region variant (38%)
				60%	chr17	7579472	G	C	65%	108%	17372.9	1853	1295	---	TP53	72	Missense	Missense tolerated (P>R), non-pathogenic
				60%	chr19	1221293	C	T	48%	80%	11481.9	5508	952	COSM29005	STK11	272	Silent	p.Y272Y
				60%	chr19	17954149	A	G	57%	95%	12227.7	2209	1122	---	JAK3		SNP	intronic variant
				60%	chr19	17954215	G	T	58%	97%	15393.8	2227	1160	COSM34216	JAK3	132	Missense	p.P132T
				60%	chr22	24176287	G	A	55%	91%	14071.2	2639	1093	COSM1090	SMARCB1	?	Intronic Substitution	p.?
PF285	12%	ubular Adenon		20%	chr2	212812097	T	C	54%	272%	5057.25	879	477	---	ERBB4	?	SNP	Intronic variant (33%)
				20%	chr3	178917005	A	G	45%	224%	3134.45	736	329	---	PIK3CA	?	SNP	Downstream gene variant (22%)
				20%	chr4	1807894	G	A	100%	500%	32009.5	3100	1997	---	FGFR3	651	SNP	Synonymous variant
				20%	chr4	1807922	G	A	60%	298%	11811.5	2421	1010	---	FGFR3		SNP	intronic variant
				20%	chr4	55141055	A	G	100%	500%	30671.9	1675	1692	---	PDGFRA	567	SNP	Synonymous variant (3%)
				20%	chr5	112175162	C	T	10%	50%	372.11	756	76	COSM19072	APC	1291	Nonsense	p.Q1291*
				20%	chr5	112175770	G	A	100%	500%	35806.3	3426	1982	---	APC	1493	SNP	Synonymous
				20%	chr5	149433596	TG	GA	26%	129%	3203.21	1836	474	---	CSF1R	?	SNP	Downstream gene variant
				20%	chr5	170837514	-	T	96%	479%	313.97	1266	1507	---	NPM1		insertion	intronic variant

				20%	chr7	55249063	G	A	100%	500%	14404.8	906	906	---	EGFR	?	SNP	Intronic variant (42%)
				20%	chr10	43613843	G	T	98%	492%	30868.2	3089	1964	---	RET	769	SNP	Synonymous variant (28%)
				20%	chr10	43615633	C	G	51%	255%	9751.38	1858	952	---	RET	904	SNP	Synonymous variant (16%)
				20%	chr11	108138003	T	C	49%	247%	12074.9	9249	985	COSM21826	ATM	858	Missense	p.F858L
				20%	chr13	28610183	A	G	55%	274%	11718.5	8213	1095	---	FLT3	?	SNP	Splice region variant (38%)
				20%	chr17	7579472	G	C	90%	452%	30271.7	2042	1807	---	TP53	72	Missense	Missense (P/R) - Tolerated
				20%	chr18	48586344	C	T	49%	244%	9732.99	5388	972	---	SMAD4	?	SNP	Intronic variant (5%)
PF287	38%	Tubulovillous		50%	chr3	178917005	A	G	100%	200%	22826.5	1448	1438	---	PIK3CA	?	SNP	Downstream gene variant (22%)
				50%	chr3	178927410	A	G	56%	111%	11960.7	2122	1109	---	PIK3CA	391	SNP	Missense (I>M) - Tolerated
				50%	chr4	1807894	G	A	100%	200%	31908.9	2452	1994	---	FGFR3	651	SNP	Synonymous variant (4%)
				50%	chr4	55141055	A	G	100%	200%	30812.6	1680	1696	---	PDGFRA	567	SNP	Synonymous variant (4%)
				50%	chr4	55597458	A	G	47%	94%	9218.04	6213	939	---	KIT		SNP	intronic variant
				50%	chr4	55962546	-	G	100%	200%	24083.4	4283	1959	---	KDR	?	SNP	Noncoding region, unknown
				50%	chr4	55972996	A	C	45%	89%	8442.76	2739	887	---	KDR		SNP	intronic variant
				50%	chr4	55980239	C	T	34%	68%	3384.74	1229	418	---	KDR	?	SNP	Intronic variant (48%)
				50%	chr5	112175770	G	A	54%	108%	13576.2	3229	1065	---	APC	1493	SNP	Synonymous
				50%	chr5	112175957	A	-	31%	62%	3250.54	2939	605	COSM18576	APC	1556	Frameshift del	p.T1556fs*9
				50%	chr5	149433596	TG	GA	100%	200%	21318.7	1334	1335	---	CSF1R	?	SNP	Downstream gene variant
				50%	chr7	55211073	A	G	50%	100%	10129.4	6248	997	---	EGFR	106	Missense	p.I106V predicted to be tolerated
				50%	chr7	55249063	G	A	100%	200%	18045.3	1137	1135	---	EGFR	?	SNP	Intronic variant (42%)
				50%	chr10	43613843	G	T	55%	109%	11685.7	2412	1092	---	RET	769	SNP	Synonymous variant (28%)
PF288	-8%	ubular Adenon		40%	chr2	212812097	T	C	60%	151%	9001.86	1322	799	---	ERBB4	?	SNP	Intronic variant (33%)
				40%	chr4	1807894	G	A	100%	250%	24010.9	1501	1505	---	FGFR3	?	SNP	Protein coding, synonymous variant
				40%	chr4	1807922	G	A	54%	135%	8523.37	1350	804	---	FGFR3		SNP	intronic variant
				40%	chr4	55141055	A	G	100%	250%	34805.6	1894	1918	---	PDGFRA	?	SNP	Protein coding, synonymous variant
				40%	chr4	55152040	C	T	49%	121%	11780.1	3521	970	COSM22413	PDGFRA	824	Silent	p.V824V
				40%	chr4	55593464	A	C	50%	126%	12376	4724	1002	COSM28026	KIT	541	Missense	p.M541L
				40%	chr4	55980239	C	T	100%	250%	15778	992	989	---	KDR	?	SNP	Intronic variant
				40%	chr5	112174577	C	T	32%	80%	6271.33	5320	635	COSM13872	APC	1096	Nonsense	p.Q1096*
				40%	chr5	112175770	G	A	50%	125%	12232.3	4009	994	---	APC	1493	SNP	Synonymous variant
				40%	chr5	149433596	TG	GA	100%	250%	14067.4	887	886	---	CSF1R	?	SNP	Downstream gene variant
				40%	chr5	170837526	T	C	6%	15%	376.44	1726	113	---	NPM1		SNP	intronic variant
				40%	chr5	170837514	-	T	94%	235%	376.44	1726	1748	---	NPM1		insertion	intronic variant
				40%	chr7	55249063	G	A	100%	250%	20691.9	1286	1293	---	EGFR	?	SNP	Protein coding, synonymous variant
				40%	chr10	43613843	G	T	100%	250%	31953.9	2345	1992	---	RET	769	SNP	Synonymous variant (28%)
				40%	chr13	28610183	A	G	52%	131%	10865.3	5736	1047	---	FLT3	?	SNP	Splice region variant (38%)
				40%	chr17	7579472	G	C	100%	250%	31903.7	1887	1758	---	TP53	72	Missense	Missense variant (P/R) - Tolerated
				40%	chr19	1220321	T	C	54%	135%	7144.25	1240	673	---	STK11	?	SNP	Intronic variant (35%)
PF289	1%	ubular Adenon		30%	chr4	1807894	G	A	100%	333%	19666.9	1225	1230	---	FGFR3	651	SNP	Synonymous variant (4%)
				30%	chr4	55141055	A	G	100%	333%	34970.1	1956	1925	---	PDGFRA	567	SNP	Synonymous variant (4%)
				30%	chr4	55962546	-	G	42%	140%	4850.52	3957	833	---	KDR	?	Insertion	Noncoding region, unknown
				30%	chr4	55972974	T	A	49%	163%	9789.96	3437	975	---	KDR	472	Missense	Missense (Q/H) - Tolerated
				30%	chr4	55980239	C	T	100%	333%	17872	1122	1122	---	KDR	?	SNP	Intronic variant (49%)
				30%	chr4	55980288	G	A	20%	68%	2322.29	2132	405	---	KDR		SNP	intronic variant/splice region variant
				30%	chr5	112175348	G	T	45%	150%	10456.9	3616	893	COSM19048	APC	1353	Nonsense	p.E1353*
				30%	chr5	112175770	G	A	69%	229%	19470.6	3981	1359	---	APC	1493	SNP	Synonymous
				30%	chr5	149433596	TG	GA	100%	333%	12517.2	789	789	---	CSF1R	?	SNP	Downstream gene variant
				30%	chr10	43613843	G	T	49%	163%	9843.62	2031	979	---	RET	769	SNP	Synonymous variant (28%)
				30%	chr11	534242	A	G	50%	167%	9360.01	1515	765	COSM249860	HRAS	27	Silent	p.H27H
				30%	chr13	28610183	A	G	50%	167%	10234.6	5618	1005	---	FLT3	?	SNP	Splice region variant (38%)
				30%	chr17	7579472	G	C	90%	301%	26884.2	1793	1621	---	TP53	72	SNP	Missense (P/R) - Tolerated
				30%	chr18	48586344	C	T	50%	167%	10232	4919	1005	---	SMAD4	?	SNP	Intronic variant (5%)
PF290	110%	ubular Adenon		40%	chr1	115258748	C	A	10%	25%	998.62	5159	196	---	NRAS	12	missense	p.G12C
				40%	chr1	115258748	C	A	10%	25%	955.36	5188	198	COSM562	NRAS	12	Missense	p.G12C
				40%	chr3	178917005	A	G	49%	122%	6115.33	1265	615	---	PIK3CA	?	SNP	Downstream gene variant (22%)
				40%	chr4	1807894	G	A	100%	250%	20747.1	1300	1304	---	FGFR3	651	SNP	Synonymous variant (4%)
				40%	chr4	55141055	A	G	100%	250%	29312.1	1590	1616	---	PDGFRA	567	SNP	Synonymous variant (4%)
				40%	chr4	55152040	C	T	47%	118%	11273.7	3237	941	COSM22413	PDGFRA	824	Silent	p.V824V
				40%	chr4	55593464	A	C	51%	128%	12745.9	5401	1021	COSM28026	KIT	541	Missense	p.M541L
				40%	chr4	55962546	-	G	37%	93%	3825.86	3672	722	---	KDR	?	Insertion	Noncoding region, unknown
				40%	chr4	55972974	T	A	52%	130%	10809.3	3237	1039	---	KDR	472	Missense	Missense (Q>H) - Tolerated

				40%	chr4	55980239	C	T	100%	250%	15031	959	951	---	KDR	?	SNP	Intronic variant (48%)
				40%	chr5	112175767	CGGAAAG	-	39%	98%	10038.9	3764	762	---	APC	1492	Deletion	ATES?/A deletes a T,E,S at codons 1492, 1493, 1494 ?
				40%	chr5	149433596	TG	GA	100%	250%	12540.3	781	782	---	CSF1R	?	SNP	Downstream gene variant
				40%	chr7	55249063	G	A	49%	121%	4528.13	927	454	---	EGFR	?	SNP	Intronic variant (42%)
				40%	chr10	43613843	G	T	100%	250%	32033.8	2266	1997	---	RET	769	SNP	Synonymous variant (28%)
				40%	chr11	534242	A	G	45%	113%	8555.8	1611	734	COSM249860	HRAS	27	Silent	p.H27H
				40%	chr17	7579472	G	C	93%	232%	23579.6	1497	1383	---	TP53	72	Missense	Missense (P/R) - Tolerated
				40%	chr22	24176287	G	A	50%	124%	9693.33	1581	789	COSM1090	SMARCB1	?	Intronic Substitution	
PF293	28%	Hyperplastic		50%	chr2	212812106	C	T	9%	18%	40.96	137	12	---	ERBB4		SNP	intronic variant
				50%	chr3	178917005	A	G	39%	77%	335.53	101	39	---	PIK3CA	?	SNP	Downstream gene variant (22%)
				50%	chr4	1807894	G	A	100%	200%	27254.7	1706	1706	---	FGFR3	651	SNP	Synonymous variant (4%)
				50%	chr4	55141055	A	G	100%	200%	3027.02	166	170	---	PDGFRA	567	SNP	Synonymous variant (4%)
				50%	chr4	55152040	C	T	38%	77%	3275.83	802	307	COSM22413	PDGFRA	824	Silent	p.V824V
				50%	chr4	55593464	A	C	43%	85%	9189.63	1895	810	COSM28026	KIT	541	Missense	p.M541L
				50%	chr4	55962546	-	G	51%	102%	6397.37	2615	916	---	KDR	?	Insertion	Noncoding region, unknown
				50%	chr4	55972974	T	A	48%	97%	1195.18	242	119	---	KDR	472	Missense	Missense (Q>H) - Tolerated
				50%	chr4	55980239	C	T	100%	200%	1752.52	115	115	---	KDR	?	SNP	Intronic variant (48%)
				50%	chr5	112175398	C	T	5%	10%	35.7	222	11	---	APC	1369	Silent	p.P1369P
				50%	chr5	112175409	C	T	5%	10%	34.35	231	11	---	APC	1373	Missense	p.P1373L
				50%	chr5	112175770	G	A	41%	82%	1291.8	278	115	---	APC	1493	SNP	Synonymous variant
				50%	chr5	112175963	G	A	6%	12%	36.01	273	17	---	APC	1558	Missense	p.D1558N
				50%	chr5	149433596	TG	GA	94%	187%	2733.41	202	189	---	CSF1R	?	SNP	Downstream gene variant
				50%	chr7	55249063	G	A	31%	61%	228.64	95	29	---	EGFR	?	SNP	Intronic variant (42%)
				50%	chr7	128851599	G	A	5%	9%	42.15	875	41	---	SMO	462	Missense?	p.V462M or intronic variant?
				50%	chr7	140453136	A	T	22%	45%	383	219	49	COSM476	BRAF	600	Missense	p.V600E
				50%	chr9	21971136	G	A	6%	12%	52.83	255	15	---	CDKN2A	74	Silent	p.D74D
				50%	chr9	21971136	G	A	6%	12%	53.12	257	15	COSM12739	CDKN2A	74	Silent	p.D74D
				50%	chr9	139390805	G	A	11%	23%	109.2	245	28	---	NOTCH1	2462	silent	p.P2462P
				50%	chr10	43613843	G	T	98%	196%	21607.1	1419	1389	---	RET	769	SNP	Synonymous variant (28%)
				50%	chr10	43615661	C	T	16%	32%	63.41	76	12	---	RET			intronic variant
				50%	chr11	534242	A	G	54%	108%	13847.1	2863	1081	COSM249860	HRAS	27	Silent	p.H27H
				50%	chr12	25398207	C	T	5%	9%	50.35	1114	52	---	KRAS			splice donor variant - predicted to have high impact
				50%	chr13	28602308	G	A	5%	10%	31.23	433	22	---	FLT3			intronic variant
				50%	chr13	28602369	G	-	97%	194%	6677.12	430	418	---	FLT3	667	frameshift deletion	p.Q667?
				50%	chr14	105246554	C	T	5%	10%	74.87	605	29	---	AKT1			splice acceptor variant, predicted to have high impact
				50%	chr17	7578368	C	T	5%	9%	153.23	1449	71	---	TP53			intronic variant
				50%	chr17	7579472	G	C	100%	200%	1047.75	62	62	---	TP53	72	Missense	Missense (P/R) - Tolerated
				50%	chr19	1220357	G	A	8%	15%	51.26	248	19	---	STK11			intronic variant
				50%	chr19	1220400	G	A	6%	13%	33.22	239	15	---	STK11	107/165	Missense	E>K, exact codon unknown
				50%	chr19	1223111	G	A	6%	12%	42.75	409	24	---	STK11		SNP	downstream gene variant
				50%	chr22	24176287	G	A	38%	77%	588.68	149	57	COSM1090	SMARCB1	?	Intronic Substitution	p.?
PF295	-48%	Sessile Serrated Adenoma		30%	chr4	1807894	G	A	100%	333%	11885.5	740	742	---	FGFR3	651	SNP	Synonymous variant (4%)
				30%	chr4	55141055	A	G	100%	333%	17045.3	933	938	---	PDGFRA	567	SNP	Synonymous variant (4%)
				30%	chr4	55593464	A	C	50%	165%	12154.6	2166	990	COSM28026	KIT	541	Missense	p.M541L Tolerated
				30%	chr4	55946354	G	T	52%	173%	10313.8	1917	997	---	KDR	?	SNP	Intronic variant
				30%	chr4	55980239	C	T	100%	333%	8654.61	549	549	---	KDR	?	SNP	Intronic variant (48%)
				30%	chr5	112175770	G	A	100%	333%	36339.1	2081	1994	---	APC	1493	SNP	Synonymous
				30%	chr5	149433596	TG	GA	100%	333%	6289.21	402	401	---	CSF1R	?	SNP	Downstream gene variant
				30%	chr7	55221882	C	T	49%	163%	6566.54	1335	655	---	EGFR			intronic variant
				30%	chr7	55249063	G	A	51%	168%	2884.56	564	285	---	EGFR	?	SNP	Intronic variant (42%)
				30%	chr7	140453136	A	T	17%	56%	1111.05	955	161	COSM476	BRAF	600	Missense	p.V600E
				30%	chr10	43613843	G	T	100%	333%	20455.5	1273	1279	---	RET	769	SNP	Synonymous variant (28%)
				30%	chr11	534242	A	G	49%	164%	4751.91	800	395	COSM249860	HRAS	27	Silent	p.H27H
				30%	chr13	28602292	T	C	48%	160%	9507.84	2144	958	---	FLT3	?	SNP	Intronic variant (4%)
				30%	chr13	28610183	A	G	100%	333%	31794.4	2410	1992	---	FLT3	?	SNP	Splice region variant (38%)
				30%	chr17	7579472	G	C	52%	174%	5757.22	697	448	---	TP53	72	Missense	Missense (P/R) - Tolerated
PF296	-43%	Hyperplastic		35%	chr2	209113154	G	A	7%	21%	288.65	2916	145	---	IDH1	118	Missense	p.P118L, conflicting predicted results

				35%	chr4	1807894	G	A	100%	286%	31970.5	3095	1996	---	FGFR3	651	SNP	Synonymous variant (4%)
				35%	chr4	55141055	A	G	100%	286%	15902.5	879	885	---	PDGFRA	567	SNP	Synonymous variant (4%)
				35%	chr4	55561713	T	C	5%	14%	91.24	1847	91	---	KIT	35	Missense	p.S35P, predicted to be deleterious
				35%	chr4	55593464	A	C	52%	150%	13213.4	6914	1047	COSM28026	KIT	541	Missense	p.M541L
				35%	chr4	55946354	G	T	49%	141%	7393.52	1488	734	---	KDR	?	SNP	Intronic variant
				35%	chr4	55980239	C	T	98%	279%	5258.78	353	345	---	KDR	?	SNP	Intronic variant (48%)
				35%	chr5	112175589	C	T	5%	13%	31.02	604	28	---	APC	1433	Missense	Missense (P>L) Deleterious
				35%	chr5	112175770	G	A	99%	283%	15113.1	845	845	---	APC	1439	SNP	Synonymous variant
				35%	chr5	149433596	TG	GA	97%	278%	11052.9	732	713	---	CSF1R	?	SNP	Downstream gene variant
				35%	chr7	55221882	C	T	41%	116%	5630.79	1535	625	---	EGFR			intronic variant
				35%	chr7	55249063	G	A	52%	147%	2647.95	503	260	---	EGFR	?	SNP	Intronic variant (42%)
				35%	chr7	140453136	A	T	19%	55%	1271.91	878	169	COSM476	BRAF	600	Missense	p.V600E
				35%	chr10	43613843	G	T	100%	286%	32065.6	3848	1999	---	RET	769	SNP	Synonymous variant (28%)
				35%	chr11	534242	A	G	55%	156%	14059.7	4747	1092	COSM249860	HRAS	27	Silent	p.H27H
				35%	chr11	108236261	A	C	5%	13%	71.65	1948	88	---	ATM			3' UTR variant
				35%	chr13	28602292	T	C	50%	142%	10065.7	6272	993	---	FLT3	?	SNP	Intronic variant (4%)
				35%	chr13	28610183	A	G	100%	286%	31986.2	9446	1997	---	FLT3	?	SNP	Splice region variant (38%)
				35%	chr17	7579472	G	C	57%	163%	1737.34	188	131	---	TP53	72	Missense	Missense (P/R) - Tolerated
				35%	chr22	24176287	G	A	5%	15%	60.32	385	20	COSM1090	SMARCB1	?	Intronic Substitution	p.?
PF297	-30%	Sessile Serrated Adenoma		25%	chr4	1807894	G	A	100%	400%	13422.2	851	850	---	FGFR3	651	SNP	Synonymous variant (4%)
				25%	chr4	55141055	A	G	100%	400%	21148.5	1170	1173	---	PDGFRA	567	SNP	Synonymous variant (4%)
				25%	chr4	55593464	A	C	51%	205%	12768.8	2446	1024	COSM28026	KIT	541	Missense	p.M541L
				25%	chr4	55946354	G	T	48%	192%	9452.92	2089	953	---	KDR	?	SNP	Intronic variant
				25%	chr4	55980239	C	T	100%	400%	10203.4	646	644	---	KDR	?	SNP	Intronic variant (48%)
				25%	chr5	112175770	G	A	100%	400%	36289.1	2364	1990	---	APC	1493	SNP	Synonymous
				25%	chr5	149433596	TG	GA	100%	400%	7744.54	493	492	---	CSF1R	?	SNP	Downstream gene variant
				25%	chr7	55221882	C	T	52%	207%	8561.41	1596	826	---	EGFR			intronic variant
				25%	chr7	55249063	G	A	46%	182%	3149.9	721	329	---	EGFR	?	SNP	Intronic variant (42%)
				25%	chr7	140453136	A	T	17%	69%	1449.89	1212	208	COSM476	BRAF	600	Missense	p.V600E
				25%	chr10	43613843	G	T	100%	400%	23157	1449	1452	---	RET	769	SNP	Synonymous variant (28%)
				25%	chr11	534242	A	G	50%	202%	6839.78	1090	552	COSM249860	HRAS	27	Silent	p.H27H
				25%	chr13	28602292	T	C	46%	182%	8761.53	2449	909	---	FLT3	?	SNP	Intronic variant (4%)
				25%	chr13	28610183	A	G	100%	400%	31963.7	2770	1997	---	FLT3	?	SNP	Splice region variant (38%)
				25%	chr17	7579472	G	C	53%	212%	7742.51	908	602	---	TP53	72	Missense	Missense (P/R) - Tolerated
PF298	-21%	Hyperplastic		45%	chr4	1807894	G	A	100%	222%	14177.6	886	886	---	FGFR3	651	SNP	Synonymous variant (4%)
				45%	chr4	55141055	A	G	100%	222%	23918.8	1308	1321	---	PDGFRA	567	SNP	Synonymous variant (4%)
				45%	chr4	55593464	A	C	50%	111%	12229.5	3066	995	COSM28026	KIT	541	Missense	p.M541L
				45%	chr4	55946354	G	T	49%	110%	9959.21	3460	987	---	KDR	?	SNP	Intronic variant
				45%	chr4	55980239	C	T	100%	222%	11956.8	754	755	---	KDR	?	SNP	Intronic variant (48%)
				45%	chr5	112175770	G	A	100%	222%	36289.2	2952	1989	---	APC	1493	SNP	Synonymous
				45%	chr5	149433596	TG	GA	100%	222%	7274.77	460	460	---	CSF1R	?	SNP	Downstream gene variant
				45%	chr7	55221882	C	T	51%	112%	10132.7	1966	995	---	EGFR			intronic variant
				45%	chr7	55249063	G	A	52%	114%	4050.13	765	396	---	EGFR	?	SNP	Intronic variant (42%)
				45%	chr7	140453136	A	T	22%	48%	2557.3	1473	318	COSM476	BRAF	600	Missense	p.V600E
				45%	chr10	43613843	G	T	100%	222%	22619.7	1414	1418	---	RET	769	SNP	Synonymous variant (28%)
				45%	chr11	534242	A	G	52%	116%	6212.49	940	497	COSM249860	HRAS	27	Silent	p.H27H
				45%	chr13	28602292	T	C	54%	120%	11389.8	3206	1075	---	FLT3	?	SNP	Intronic variant (4%)
				45%	chr13	28610183	A	G	100%	222%	31963.7	3819	1997	---	FLT3	?	SNP	Splice region variant (38%)
				45%	chr17	7579472	G	C	56%	123%	8742.46	991	668	---	TP53	72	Missense	Missense (P/R) - Tolerated
279	Excluded due to insufficient DNA																	
263	Excluded due to insufficient DNA																	
269	Excluded due to insufficient DNA																	
272	Excluded due to insufficient DNA																	
291	Excluded due to insufficient DNA																	
292	Excluded due to insufficient DNA																	

All variants post quality control processing. Variants were eliminated based on the following: (1) quality score less than 10, (2)allelic frequency less than 5% or number of allele reads less than 10, (3) a known mispriming event for this panel [PMID:25017478], or (4) strand bias was recorded.
