# Supplementary material for: Subclonal diversity arises early even in small colorectal tumours and contributes to differential growth fates
Source: Gut. 2016 Sep 8;66(12):2132–40. doi: 10.1136/gutjnl-2016-312232 (PMC5342955; doi:10.1136/gutjnl-2016-312232)
Supplement: supplementary figures and tables [file gutjnl-2016-312232supp001.pdf]

Supplementary Table S1. Pathogenic mutation classification.
Mutations can be classified as public (≥30% allele frequency) or private (<30% allele frequency) and each polyp is classified as harboring multiple public, single public, public and private, or only private mutations.

Multiple Public

Sample	Adjusted Frequency	Gene.ID
PF270	100%	FBXW7
	100%	TP53
PF245	92%	RB1
	88%	APC
PF256	100%	KRAS
	57%	FBXW7
PF254	100%	TP53
	38%	FBXW7
PF250	100%	APC
	34%	KRAS
PF259	100%	APC
	31%	TP53
PF08	59%	EGFR
	48%	APC
PF25	58%	APC
	55%	APC
PF264	46%	APC
	41%	APC

Single Public

Sample	Adjusted Frequency	Gene.ID
PF289	100%	APC
PF249	100%	APC
PF255	100%	BRAF
PF278	89%	APC*
	89%	APC*
PF288	80%	APC
PF14	78%	APC
PF241	79%	KRAS
PF273	76%	APC
PF01	37%	APC
PF04	67%	APC
PF12	40%	APC
PF19	36%	APC
PF21	38%	APC
PF22	37%	APC
PF23	35%	APC
PF239	52%	BRAF
PF285	50%	APC
PF287	62%	APC
PF295	56%	BRAF
PF297	69%	BRAF
PF298	48%	BRAF

Public + Private

Sample	Adjusted Frequency	Gene.ID
PF290	98%	APC
	25%	NRAS
PF24	86%	APC
	12%	KRAS
PF296	55%	BRAF
	13%	APC
PF15	47%	APC
	43%	APC
	6%	KRAS
PF293	45%	BRAF
	12%	APC
	10%	APC
	9%	KRAS
PF13	33%	APC
	12%	APC
	5%	FBXW7

Only Private

Sample	Adjusted Frequency	Gene.ID
PF18	26%	RB1
	8%	KRAS
	5%	TP53
PF276	11%	FGFR3
	10%	FBXW7
	10%	APC
PF274	28%	APC
PF17	24%	BRAF
PF279	22%	APC
PF20	17%	APC
PF06	9%	APC

Supplementary Table S2. Detectable mutations generated by statistical inference.
Four sets of parameters were used to generate *in silico* polyps with detectable mutations. Baseline parameters used were mutation rate distribution = $5x10^{-4}$ mutations per crypt fission event and a fitness change distribution = N(0, 0.2). These two parameters were then modified to further investigate the finding that detectable mutations arose when the tumor was small.

Mutation Rate	Fitness Change Distribution	n*	Median Size	Mean Size	Standard Deviation (Size)	Median Fitness Change	Mean Fitness Change	Standard Deviation (Fitness Change)
$5x10^{-4}$	N(0, 0.2)	6,429	18	30	35	0.174	0.176	0.167
$5x10^{-2}$	N(0, 0.2)	315,878	18	30	45	0.162	0.164	0.173
$5x10^{-4}$	N(2, 0.2)	47,389	125	169	158	2.015	2.015	0.20
$5x10^{-2}$	N(2, 0.2)	837,017	25	37	38	2.011	2.011	0.20

*Total number of mutations detectable in the slice, generated across 1,000,000 polyps

	MSI Analysis System 2.0 (prototype)													
**Sample Id**	**NR-21**	**BAT-25**	**BAT-26**	**Mon-27**	**BAT-52**	**BAT-56**	**BAT-59**	**BAT-60**	**Penta C**	**Penta D**	**Positive**	**Inform**	**Result**	**Comments**
PF01	0	0	0	0	0	0	0	0	0	0	0	8	MSS	
PF02	0	0	0	0	0	0	0	0	0	0	0	8	MSS	
PF03	0	0	0	0	0	0	0	0	0	0	0	8	MSS	
PF04	0	0	0	0	0	0	0	0	0	0	0	8	MSS	
PF05	0	0	0	0	0	0	0	0	0	0	0	8	MSS	
PF06	0	0	0	0	0	0	0	0	0	0	0	8	MSS	
PF07	0	0	0	0	0	0	0	0	0	0	0	8	MSS	
PF08	0	0	0	0	0	0	0	0	0	0	0	8	MSS	
PF09	0	0	0	0	0	0	0	0	0	0	0	8	MSS	
PF10	0	0	0	0	0	0	0	0	0	0	0	8	MSS	
PF11	0	0	0	0	0	0	0	0	0	0	0	8	MSS	
PF12	0	0	0	0	0	0	0	0	0	0	0	8	MSS	
PF13	0	0	0	0	0	0	0	0	0	0	0	8	MSS	
PF14	0	0	0	0	0	0	0	0	0	0	0	8	MSS	
PF15	0	0	0	0	0	0	0	0	0	0	0	8	MSS	
PF16	0	0	0	0	0	0	0	0	0	0	0	8	MSS	
PF17	0	0	0	0	0	0	0	0	0	0	0	8	MSS	
PF18	0	0	0	0	1	0	0	1	0	0	2	6	MSI+	BAT-52 and BAT-60 with 3 alleles
PF19	0	0	0	1	0	0	1	0	0	1	2	7	MSI+	MONO-27 2 alleles, BAT-59 and Penta D 3 alleles
PF20	0	0	0	0	0	0	0	0	0	0	0	8	MSS	
PF21	0	0	0	0	0	0	0	0	0	0	0	8	MSS	
PF22	0	0	0	0	0	0	0	0	0	0	0	8	MSS	
PF23	0	0	0	0	0	0	0	0	0	0	0	8	MSS	
PF24	0	0	0	0	0	0	0	0	0	0	0	8	MSS	
PF25	0	0	0	0	0	0	0	0	0	0	0	8	MSS	
PF26	0	0	0	0	0	0	0	0	0	0	0	8	MSS	
PF 239	0	0	0	0	0	0	0	0	0	0	0	8	MSS	
PF 241	0	0	1	0	0	0	0	0	nd	nd	1	8	EQ	BAT-26 2 alleles
PF 245	0	0	0	0	0	0	0	0	0	0	0	8	MSS	
PF 249	0	1	0	0	0	0	0	0	0	0	1	8	EQ	BAT-25 2 alleles, 249 and 250 same
PF 250	0	1	0	0	0	0	0	0	0	0	1	8	EQ	
PF 252	0	0	0	0	0	0	0	0	nd	0	0	8	MSS	
PF 254	0	0	0	0	0	0	0	0	0	0	0	8	MSS	
PF 255	0	0	0	0	0	0	0	0	0	0	0	8	MSS	
PF 256	0	0	0	0	0	0	0	0	0	0	0	8	MSS	
PF 259	0	0	0	0	0	0	0	0	0	0	0	8	MSS	
PF 263	nd	nd	nd	nd	nd	nd	nd	nd	nd	nd	0	0	nd	no sample
PF 264	0	0	0	0	0	0	0	0	0	0	0	8	MSS	
PF 269	0	0	0	0	0	0	0	0	0	0	0	8	MSS	
PF 270	0	0	0	0	0	0	0	0	1	0	0	8	EQ	3 alleles for Penta C
PF 272	0	0	0	0	0	0	0	0	0	0	0	8	MSS	
PF 273	0	0	0	0	0	0	0	0	0	0	0	8	MSS	
PF 274	0	0	0	0	0	0	0	0	0	0	0	8	MSS	
PF 276	0	0	0	0	0	0	0	0	0	0	0	8	MSS	
PF 277	0	0	0	0	0	0	0	0	0	0	0	8	MSS	

PF 278	0	0	0	0	0	0	0	0	0	0	0	8	MSS	
PF 279	0	0	0	0	0	0	0	0	0	0	0	8	MSS	
PF 285	0	0	0	0	0	0	0	0	0	0	0	8	MSS	
PF 287	0	0	0	0	0	0	0	0	0	0	0	8	MSS	
PF 288	0	0	0	0	0	0	0	0	0	0	0	8	MSS	
PF 289	0	0	0	0	0	0	0	0	0	0	0	8	MSS	
PF 290	1	0	0	0	0	0	0	0	0	0	1	8	EQ	2 allels fro NR-21, 290-293 same
PF 291	1	0	0	0	nd	nd	nd	nd	nd	nd	1	4	EQ	2 allels fro NR-21
PF 292	1	0	0	0	0	0	0	0	0	0	1	8	EQ	2 allels fro NR-21
PF 293	1	0	0	0	0	0	nd	0	nd	nd	1	7	EQ	2 allels fro NR-21
PF 295	0	0	0	0	0	0	0	0	0	0	0	8	MSS	
PF 296	0	0	0	0	0	0	0	0	0	0	0	8	MSS	
PF 297	0	0	0	0	0	0	0	0	0	0	0	8	MSS	
PF 298	0	0	0	0	0	0	0	0	0	0	0	8	MSS	

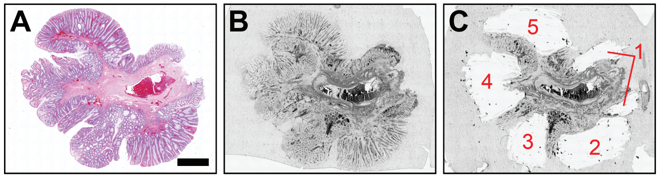

D. Mutations

Sample	APC p.E1464fs*8	KRAS p.G12D
Area 1	Insufficient	Not detected
Area 2	Present	0%
Area 3	Present	>99.9%
Area 4	Present	13.9%
Area 5	Present	Not detected
Whole	Present	1.1%

E.

					Micro-Dissected Regions				
Sample	Gene	Mutation	Cosmic ID	Bulk	Region 1	Region 2	Region 3	Region 4	Region 5
PF03	APC	p.R876*	18852	detected	detected	not detected	not detected	not detected	
PF03	APC	p.Q1429*	18836	detected	detected	not detected	not detected	not detected	
PF 11	CTNNB1	p.S45F	5667	not detected	not detected	not detected	not detected	not detected	
PF12	APC	p.E1322*	18702	detected	detected	detected	detected	detected	detected
PF14	APC	p.E1309fc*4	13113	detected	detected	detected			
PF20	APC	p.L1488fs*18	41618	detected	detected	detected	insufficent DNA		
PF24	APC	p.E1464fs*8	18838	detected	insufficent DNA	detected	detected	detected	detected
PF24	KRAS	p.G12D	521	1.1%	not detected	0%	>99.9%	13.9%	not detected
PF25	APC	p.R876*	18852	detected	detected	detected	detected	detected	detected
PF25	APC	p.R1450*	13127	detected	detected	detected	detected	detected	detected

Notes: PF03 was later discovered to be two separate polyps combined into 1 block and was thus not used in the final analysis. PF11 contained a CTNNB1 mutation that was likely a false positive from sequencing and was thus eliminated.

Supplementary Figure S1. Spatial distribution and mutation validation was performed on previously identified mutations for which commercial primers were available. (**A**) H&E stained section of PF24 is shown as an example. (**B**) Unstained section of PF24 used to guide microdissection. (**C**) Unstained section of PF24 following micro-dissection. (**D**) Quantitative PCR results for PF24 demonstrate that the public APC mutation was present in all regions for which sufficient DNA was available. This is in contrast to the private KRAS mutation which was only detected in regions 3 and 4, and at a low level in the bulk sample. (**E**) Quantitative PCR results for all polyps that underwent micro-dissection and mutation validation are shown. Blacked out cells indicate that those polyps did not have 5 micro-dissected regions. Only polyps which had remaining FFPE tissue were available for qPCR validation, which was a minority of cases.

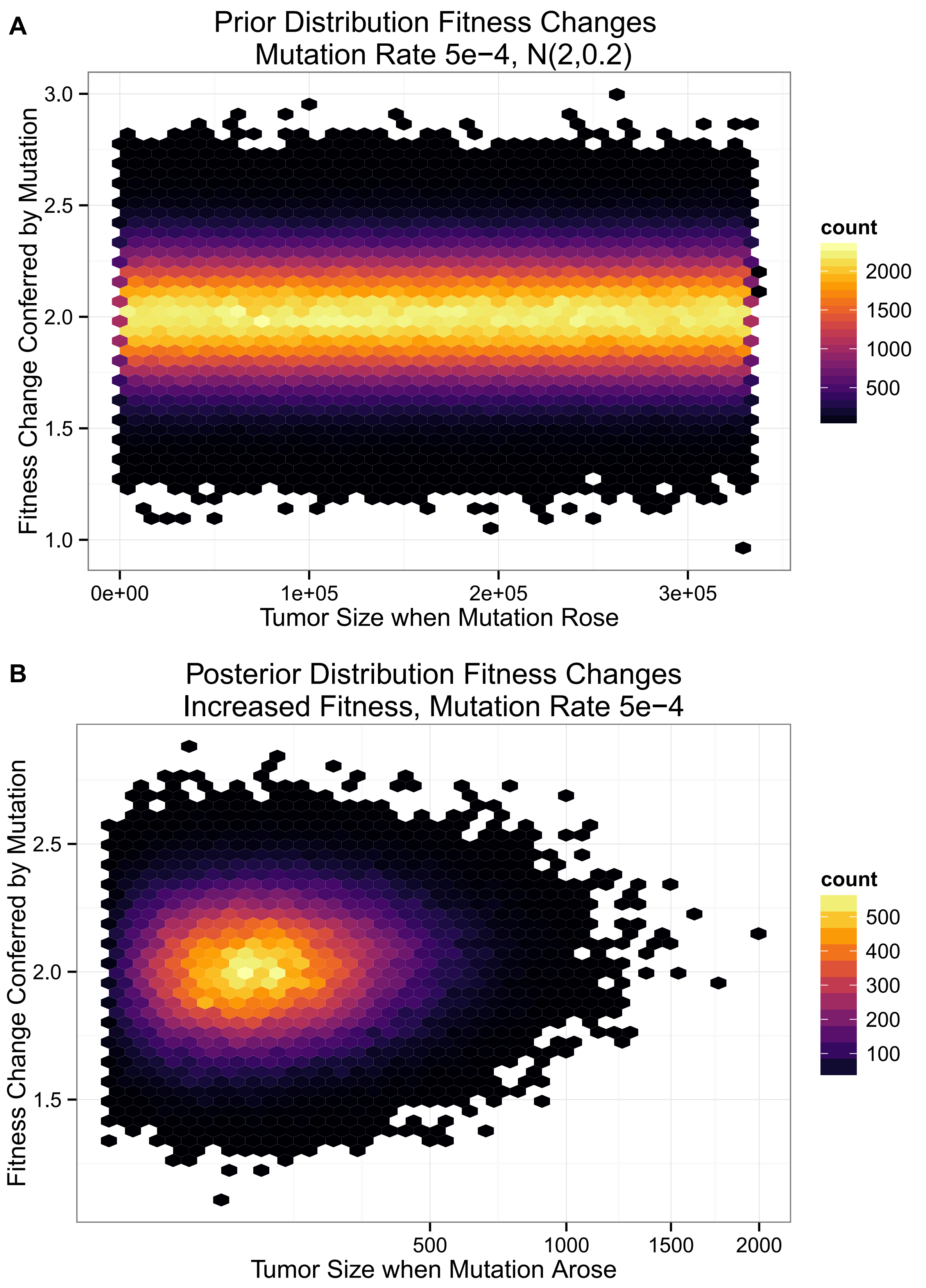

Supplementary Figure S2. Increased fitness change distribution still predicts that detectable mutations arise early. (**A**) Prior distribution of the fitness change conferred by all mutations acquired by *in silico* tumors demonstrates that mutations that affect fitness can occur at any size in the model, but center around a change of 2.0, which represents a two-fold increase in fitness. (**B**) Posterior distribution of the fitness change conferred by mutations that fit acceptance criteria and the size of the tumor when that mutation arose is shown.

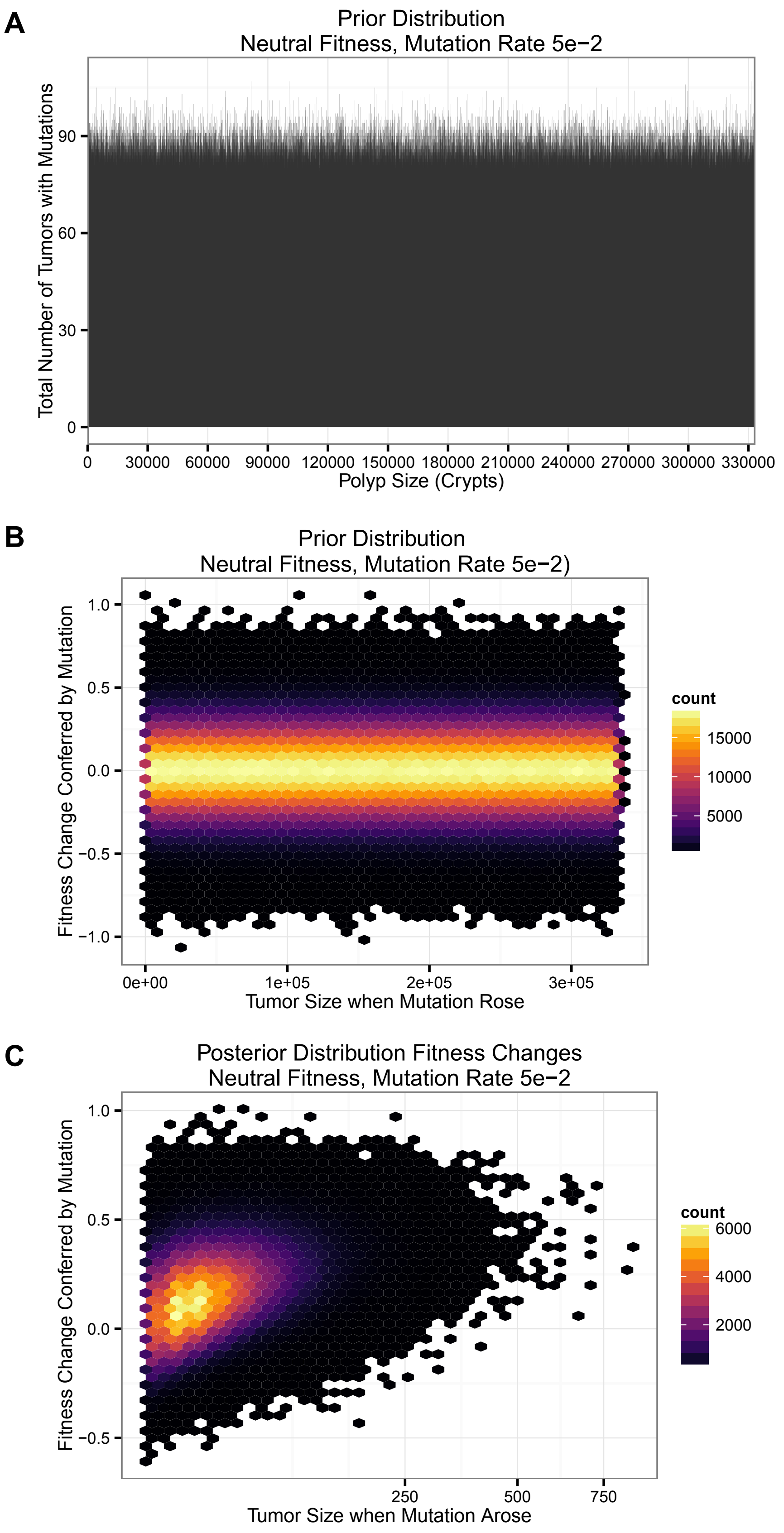

Supplementary Figure S3. Increased mutation rate distribution still predicts that detectable mutations arise early. (**A**) Prior distribution of all mutations acquired by in silico tumors demonstrates that mutations can occur at any size in the model. Note that this plot is generated from a random sampling of all mutations generated. (**B**) Prior distribution of the fitness change of all mutations acquired by in silico tumors demonstrates that fitness can be positive, neutral or negative. (**C**) Posterior distribution of the fitness change conferred by mutations that fit acceptance criteria and the size of the tumor when that mutation arose is shown.

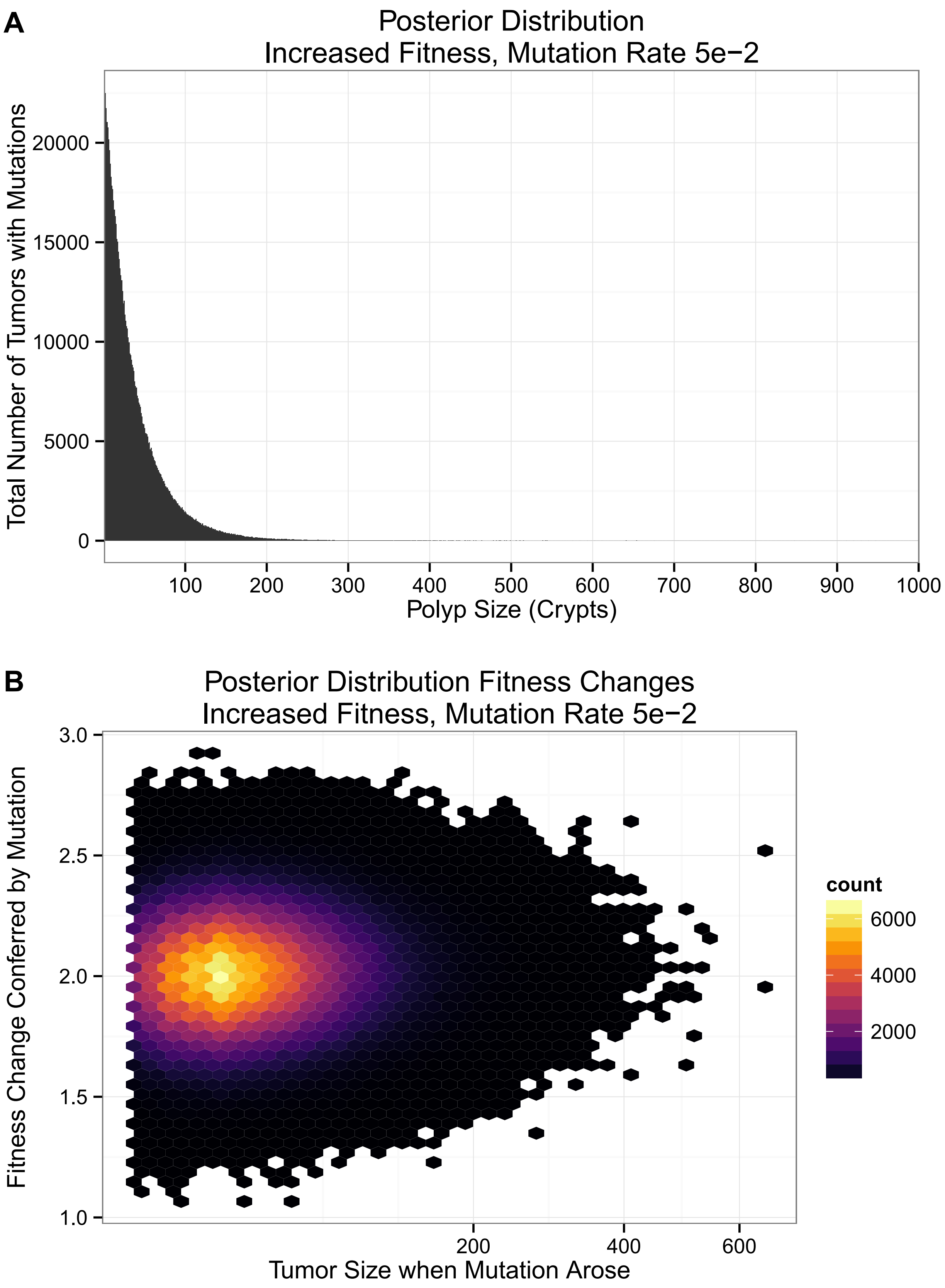

Supplementary Figure S4. Increased mutation rate distribution in combination with increased fitness change distribution still predict that detectable mutations arise early. (**A**) Posterior distribution of mutations that fit acceptance criteria and the size of the tumor when that mutation arose is shown. (**B**) Posterior distribution of the fitness change conferred by mutations that fit acceptance criteria and the size of the tumor when that mutation arose is shown.

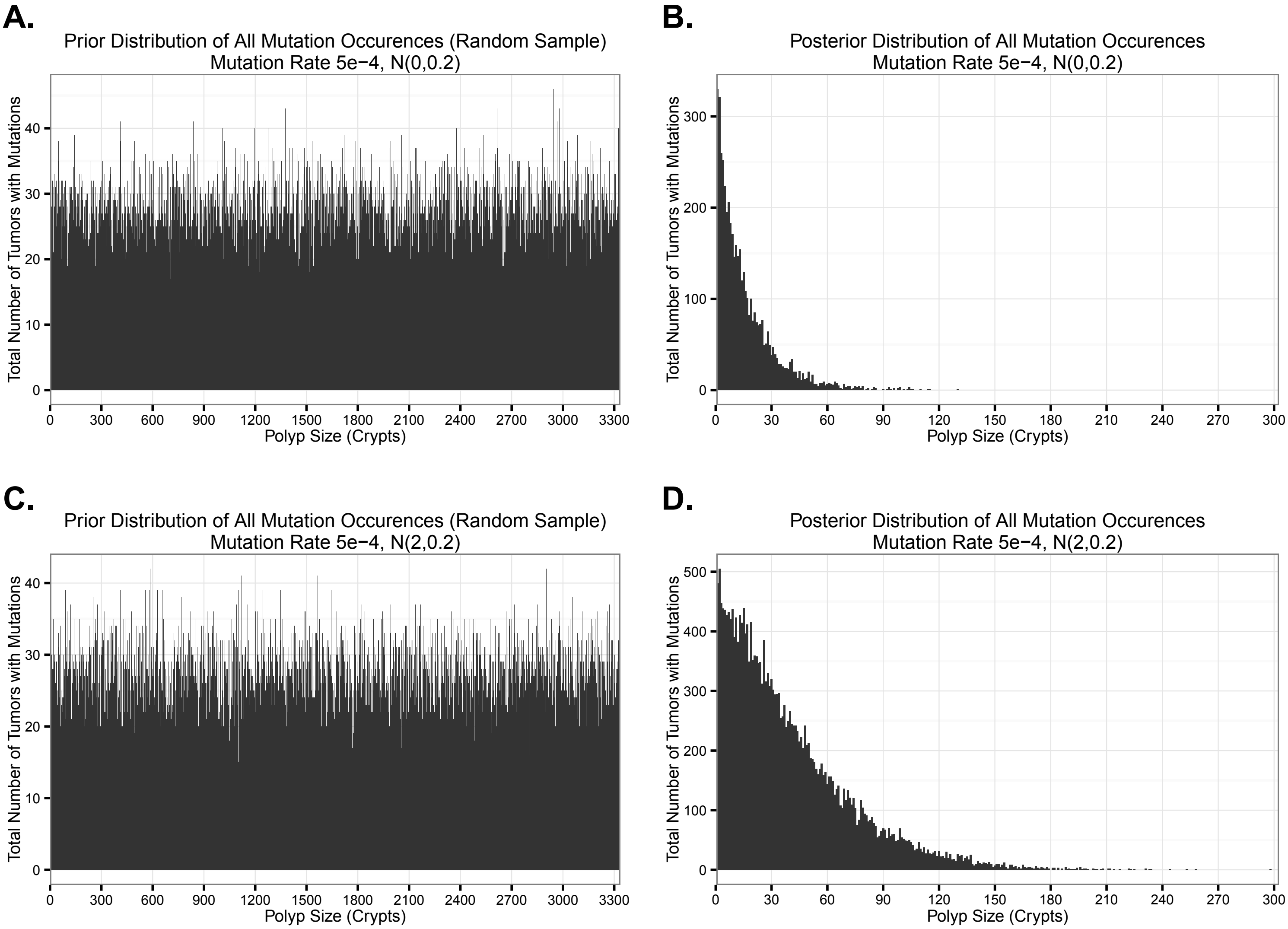

Supplemental Figure S5: Limiting final polyp size predicts that detectable mutations arise early. **(A)** Prior distribution of all mutations acquired by *in silico* tumors demonstrates that mutations can occur at any size in the baseline model when the final size is restricted to 3,333 crypts. **(B)** Posterior distribution of mutations that fit acceptance criteria from (A) and the size of the tumor when that mutation arose is shown, mean = 16 ± 16 crypts, median = 11. **(C)** Prior distribution of all mutations acquired by *in silico* tumors demonstrates that mutations can occur at any size when fitness change is forced to be positive and when the final size is restricted to 3,333 crypts. **(D)** Posterior distribution of mutations that fit acceptance criteria from (C) and the size of the tumor when that mutation arose is shown, mean = 40 ± 33 crypts, median = 31.
