# Supplementary material for: Subclonal diversity arises early even in small colorectal tumours and contributes to differential growth fates
Source: Gut. 2016 Sep 8;66(12):2132–40. doi: 10.1136/gutjnl-2016-312232 (PMC5342955; doi:10.1136/gutjnl-2016-312232)
Supplement: supplementary file [file gutjnl-2016-312232supp004.pdf]

Authors: Luli S. Zou, Bret R. Larget

Contact: luli.zou@gmail.com

Required files: tumor.C, tumor.h

Compilation method: g++ 4.9 or greater

Introduction:

This program allows the forward simulation of tumor growth given the model described in Sievers et al. (2016).

Tumors are sliced down the middle to replicate the experimental design of Sievers et al. (line 295 of tumor.C).

Computational time:

1,000,000 tumors can be generated in less than half a day using high throughput computing servers running 5,000 batches of 200 tumors.

Data generated:

Each tumor output file is labeled Òmutation_data_(seed number).csvÓ.

If the tumor dies with probability 20% in the beginning of the simulation, this .csv is empty.

Each .csv file contains the seed, mutation ID, mutation frequency in the tumor, mutation frequency in the slice, mutation frequency in the tumor,

when the mutation arose in the tumor, and the fitness change the mutation conferred to the crypt it arose in.

Required flags:

-s: integer, seed for random number generator. Must be set. Each tumor has a unique seed number which is set in line 281.

-t: integer, tumor size in crypts. Must be set, otherwise default is 0.

Possible flags:

-n: integer, number of tumors to make in a run. Default is 200 (~1 hour).

-m: double, expected number of mutations per crypt fission, i.e. the Poisson distribution parameter. Default is 5e-4.

-fm: double, the mean of the fitness change normal distribution. Default is 0.

-fsd: double, the standard deviation of the fitness change normal distribution. Default is 0.2.

-p: boolean, true = print tumor info after creation, false = do not print tumor info. Default is false.

Example:

Compilation line using Mac OS X 10.10.5 : g++ tumor.C -o tumor

Command line run: tumor -s 1 -t 333333 -m 5e-4

This creates 200 tumors of size 333,333, with unique seeds 1:200, and an expected mutation value of 5e-4, and generates a .csv file for each tumor.
