# Supplementary material for: Subclonal diversity arises early even in small colorectal tumours and contributes to differential growth fates
Source: Gut. 2016 Sep 8;66(12):2132–40. doi: 10.1136/gutjnl-2016-312232 (PMC5342955; doi:10.1136/gutjnl-2016-312232)
Supplement: supplementary file [file gutjnl-2016-312232supp005.pdf]

```
#ifndef __TUMOR_H
#define __TUMOR_H

#include <iostream>
#include <vector>
#include <random>
#include <string>

using namespace std;

class Location
{
private:
  int x,y,z;
public:
  Location() {}
  Location(vector<int> v)
  {
    x = v[0]; y = v[1]; z = v[2];
  }
  Location(int xx,int yy,int zz)
  {
    x = xx; y=yy; z=zz;
  }
  int getX() const { return x; }
  int getY() const { return y; }
  int getZ() const { return z; }
  void print(ostream&) const;

  Location& operator=(const Location& b)
  {
    x = b.getX();
    y = b.getY();
    z = b.getZ();
    return *this;
  }

  Location& operator+=(const Location& b)
  {
    x += b.getX();
    y += b.getY();
    z += b.getZ();
    return *this;
  }

  Location& operator-=(const Location& b)
  {
    x -= b.getX();
    y -= b.getY();
    z -= b.getZ();
    return *this;
  }

  const Location operator+(const Location& b) const
  {
    return Location(*this) += b;
  }

  bool operator==(const Location& b) const
  {
    if ( (x == b.getX()) && (y == b.getY()) && (z == b.getZ()) )
      return true;
    return false;
```

```
  }

  bool operator!=(const Location& b) const
  {
    return !(*this == b);
  }

  bool operator<(const Location& b) const
  {
    if ( x < b.getX() )
      return true;
    else if ( x > b.getX() )
      return false;
    else if ( y < b.getY() )
      return true;
    else if ( y > b.getY() )
      return false;
    else if ( z < b.getZ() )
      return true;
    else
      return false;
  }

  bool operator>(const Location& b) const
  {
    return ( b < *this );
  }

  bool operator<=(const Location& b) const
  {
    return !( b > *this );
  }

  bool operator>=(const Location& b) const
  {
    return !( b < *this );
  }
};

class Mutation
{
private:
  int id;
  int tumorSize;
  int numTumorCrypts;
  int numSliceCrypts;
  double fitnessChange;
public:
  Mutation() {}
  Mutation(int i,int ts, double fc)
  {
    id = i;
    tumorSize = ts;
    numTumorCrypts = 0;
    numSliceCrypts = 0;
    fitnessChange = fc;
  }
  void addTumorCrypt() { ++numTumorCrypts; }
  void subtractTumorCrypt() { --numTumorCrypts; }
  void addSliceCrypt() { ++numSliceCrypts; }
  void print(ostream&);
  int getID() { return id; }
  int getNumTumorCrypts() { return numTumorCrypts; }
```

```
  int getNumSliceCrypts() { return numSliceCrypts; }
  int getTumorSize() { return tumorSize; }
  double getFitnessChange() { return fitnessChange; }
};

class Crypt
{
private:
  const int index;
  Location location;
  double fitness;
  double deathProbability;
  vector<Mutation*> mutations;
public:
  Crypt(int i, Location p, double f, vector<Mutation*> m) : index(i)
  {
    location = p;
    fitness = f;
    mutations = m;
    setDeathProbability();
  }
  ~Crypt() {}
  double getDeathProbability() { return deathProbability; }
  void setDeathProbability()
  {
    deathProbability = 0.2 / fitness;
    if ( deathProbability < 0 )
      deathProbability = 1;
    if ( deathProbability > 1 )
      deathProbability = 1;
  }
  int getIndex() const { return index; }
  Location getLocation() const { return location; }
  void setLocation(Location p) { location = p; }
  double getFitness() const { return fitness; }
  void setFitness(double f) { fitness = f; }
  vector<Mutation*> getMutations() const { return mutations; }
  void addMutation(Mutation* m) { mutations.push_back(m); }
  int getX() const { return location.getX(); }
  int getY() const { return location.getY(); }
  int getZ() const { return location.getZ(); }
  bool operator<(const Crypt& b) const
  {
    return ( location < b.getLocation() );
  }
  void print(ostream&);
};

class Tumor
{
private:
  vector<Crypt*> crypts;
  vector<Mutation*> mutations;
  map<Location , Crypt*> cryptMap;
  int nextCryptIndex;
  double expectedMutations;
  double fitnessMean;
  double fitnessSD;
  int sliceSize;

public:
  Tumor(double em,double fm, double fsd)
  {
```

```
    nextCryptIndex = 0;
    Location loc(0,0,0);
    vector<Mutation*> m;
    crypts.push_back(new Crypt(nextCryptIndex++,loc,1.0,m) );
    cryptMap[loc] = *(crypts.begin());
    expectedMutations = em;
    fitnessMean = fm;
    fitnessSD = fsd;
    sliceSize = 0;
  }
  void print(ostream&);
  void change(mt19937_64&);
  void addCrypt(mt19937_64&,Crypt*);
  void deleteCrypt(int);
  Location randomDirection(mt19937_64&);
  int size() { return crypts.size(); }
  int numMutations() { return mutations.size(); }
  vector<Mutation*> getMutations() { return mutations; }
  map<Location , Crypt*> getCryptMap() { return cryptMap; }
  vector<Crypt*> getCrypts() { return crypts; }

  //SLICE
  void slice() {
    for (vector<Crypt*>::iterator c = crypts.begin(); c != crypts.end(); ++c) {
      Location test = (*c)->getLocation();
      if (test.getX() ==0) {
        sliceSize++;
        vector<Mutation*> copyOfMutations = (*c)->getMutations();
        for (vector<Mutation*>::iterator m = copyOfMutations.begin(); m != 
copyOfMutations.end(); ++m)
          (*m)->addSliceCrypt();
      }
    }
  }
  int getSliceSize() { return sliceSize; }


};

#endif
```
