# Supplementary material for: Subclonal diversity arises early even in small colorectal tumours and contributes to differential growth fates
Source: Gut. 2016 Sep 8;66(12):2132–40. doi: 10.1136/gutjnl-2016-312232 (PMC5342955; doi:10.1136/gutjnl-2016-312232)
Supplement: supplementary file [file gutjnl-2016-312232supp006.pdf]

```
// 3D Tumor Generation
// Authors: Luli S. Zou, Bret R. Larget
// Used in Sievers et. al. 2016
// View README before compiling/using

#include <iostream>
#include <iomanip>
#include <vector>
#include <random>
#include <string>
#include <sstream>
#include <list>
#include <map>
#include <fstream>

#include "tumor.h"

using namespace std;

void Location::print(ostream& f) const
{
  f << '(' << getX() << ',' << getY() << ',' << getZ() << ')';
}

void Crypt::print(ostream& f)
{
  f << index << ": ";
  f << "location = (" << getX() << ',' << getY() << ',' << getZ() << "), ";
  f << "fitness = " << setprecision(6) << fitness << ", ";
  f << "mutations = (";
  for ( vector<Mutation*>::iterator p=mutations.begin(); p!=mutations.end(); ++p )
  {
    if ( p != mutations.begin() )
      f << ',';
    f << *p;
  }
  f << ')' << endl;
}

void Mutation::print(ostream& f)
{
  f << id << ": ";
  f << "tumor size at creation = " << tumorSize << ", ";
  f << "# crypts = " << numTumorCrypts << endl;
}

void Tumor::print(ostream& f)
{
  //f << "Crypts:" << endl;
  //for ( vector<Crypt*>::iterator p=crypts.begin(); p!=crypts.end(); ++p )
  //{
  //  (*p)->print(f);
  //}
  //f << endl;
  f << "Mutations:" << endl;
  for ( vector<Mutation*>::iterator p=mutations.begin(); p!=mutations.end(); ++p )
  {
    (*p)->print(f);
  }
  f << endl;
  //f << "Map:" << endl;
  //for ( map<Location, Crypt*>::iterator p=cryptMap.begin(); p!=cryptMap.end(); ++p
)
```

```
  //{
  //  p->first.print(f);
  //  f << " --> " << (p->second)->getIndex() << endl;
  //}
  //f << endl;
}

Location Tumor::randomDirection(mt19937_64& rng)
{
  vector<int> v(3,0);
  uniform_int_distribution<> rint(0,17);
  int x=rint(rng);
  switch (x)
  {
  case 0 : v[0] -= 1; break;
  case 1 : v[0] += 1; break;
  case 2 : v[1] -= 1; break;
  case 3 : v[1] += 1; break;
  case 4 : v[2] -= 1; break;
  case 5 : v[2] += 1; break;
  case 6 : v[0] -= 1; v[1] -= 1; break;
  case 7 : v[0] -= 1; v[1] += 1; break;
  case 8 : v[0] += 1; v[1] -= 1; break;
  case 9 : v[0] += 1; v[1] += 1; break;
  case 10 : v[0] -= 1; v[2] -= 1; break;
  case 11 : v[0] -= 1; v[2] += 1; break;
  case 12 : v[0] += 1; v[2] -= 1; break;
  case 13 : v[0] += 1; v[2] += 1; break;
  case 14 : v[1] -= 1; v[2] -= 1; break;
  case 15 : v[1] -= 1; v[2] += 1; break;
  case 16 : v[1] += 1; v[2] -= 1; break;
  case 17 : v[1] += 1; v[2] += 1; break;
  }
  return Location(v);
}

void Tumor::change(mt19937_64& rng)
{
  // pick a random crypt
  int x=0;
  int n=crypts.size();
  if ( n > 1 )
  {
    uniform_int_distribution<> rint(0,n-1);
    x = rint(rng);
  }
  Crypt* parent = crypts[x];
  // decide to either kill crypt or initiate crypt fission
  uniform_real_distribution<double> runif(0.0,1.0);
  if ( runif(rng) < parent->getDeathProbability() )
  {
    //Update number of crypts that carry the mutation
    vector<Mutation*> parent_muts = parent->getMutations();
    for (vector<Mutation*>::iterator m = parent_muts.begin(); m !=
parent_muts.end(); ++m)
        (*m)->subtractTumorCrypt();
    deleteCrypt(x);
  }
  else
  {
    addCrypt(rng,parent);
  }
}
```

```
void Tumor::addCrypt(mt19937_64& rng,Crypt* parent)
{
  Location parentLocation = parent->getLocation();
  Location dir = randomDirection(rng);
  vector<Crypt*> displacedCrypts;
  Location newLocation = parentLocation;
  map<Location,Crypt*>::iterator mp;
  do
  {
    newLocation += dir;
    mp = cryptMap.find(newLocation);
    if ( mp != cryptMap.end() )
    {
      displacedCrypts.push_back(mp->second);
      mp->second->setLocation(newLocation + dir); // yes, the map is now wrong
    }
  } while ( mp != cryptMap.end() );
  // now we need to reverse_iterate over displacedCrypts to correct the map
  for ( vector<Crypt*>::reverse_iterator r=displacedCrypts.rbegin(); r !=
displacedCrypts.rend(); ++r )
  {
    cryptMap[(*r)->getLocation()] = *r;
  }
  // finally, create the new crypt and add it to the map
  newLocation = parentLocation + dir;

 //Possibility of mutations occurring
  vector<Mutation*> new_muts = parent->getMutations();
  double new_fitness = parent->getFitness();
  poisson_distribution<int> pd(expectedMutations);
  int numMuts = pd(rng);
  if (numMuts > 0) {
    normal_distribution<double> nd(fitnessMean, fitnessSD);
    double fitChange = nd(rng);
    new_fitness += fitChange;
    for (int i = 0; i<numMuts; i++) {
      Mutation* mut = new Mutation(mutations.size(), size(), fitChange);
      new_muts.push_back(mut);
      mutations.push_back(mut);
    }
  }
  //Update number of crypts that carry the mutation
  for (vector<Mutation*>::iterator m = new_muts.begin(); m != new_muts.end(); ++m)
      (*m)->addTumorCrypt();

  crypts.push_back( new Crypt(nextCryptIndex++,newLocation,new_fitness,new_muts) );
  cryptMap[newLocation] = crypts.back();
}

void Tumor::deleteCrypt(int k)
{
  // erase location from the map
  cryptMap.erase(crypts[k]->getLocation());
  // delete the actual crypt
  delete crypts[k];
  // overwrite deleted crypt pointer with last pointer
  crypts[k] = crypts.back();
  // eliminate end of the vector
  crypts.pop_back();
}

void usage(ostream& f)
```

```
{
  f << "Bad input" << endl;
  exit(1);
}

void setValue(string value,unsigned int &x)
{
  istringstream s(value);
  s >> x;
}

void setValue(string value,double &x)
{
  istringstream s(value);
  s >> x;
}


void processCommandLine(int argc,
                        char* argv[],
                        unsigned int &seed,
                        unsigned int &tumorSize,
                        unsigned int &numTumors,
                        double &expectedMutations,
                        double &fitnessMean,
                        double &fitnessSD,
                        bool &printTumor)
{
  if ( argc == 1 )
  {
    random_device rd;
    seed = rd();
  }
  else
  {
    int i = 1;
    while ( i < argc )
    {
      string key = argv[i++];

      if ( key=="-s" || key=="--seed" ) {
        if ( i==argc )
          usage( cerr );
        setValue(argv[i++],seed);
      }
      else if ( key=="-t" || key=="--tumor-size" ) {
        if ( i==argc )
          usage( cerr );
        setValue(argv[i++],tumorSize);
      }
      else if ( key=="-n" || key=="--number-tumors" ) {
        if ( i==argc )
          usage( cerr );
        setValue(argv[i++],numTumors);
      }
      else if ( key =="-m" || key=="--expected-mutations") {
        if ( i==argc)
          usage( cerr );
        setValue(argv[i++],expectedMutations);
      }
      else if ( key =="-fm" || key=="--fitness-mean") {
        if ( i==argc)
          usage( cerr );
```

```
        setValue(argv[i++],fitnessMean);
      }
      else if ( key =="-fsd" || key=="--fitness-SD") {
        if ( i==argc)
          usage( cerr );
        setValue(argv[i++],fitnessSD);
      }
      else if ( key=="-p" || key=="--print-tumor")
        printTumor = true;
      else
        usage(cerr);
    }
  }
}

int main(int argc, char* argv[])
{
  // Process command line
  unsigned int seed;
  unsigned int tumorSize=0;
  unsigned int numTumors=200;
  double expectedMutations = 5e-4;
  double fitnessMean = 0;
  double fitnessSD = 0.2;
  bool printTumor = false;

processCommandLine(argc,argv,seed,tumorSize,numTumors,expectedMutations,fitnessMean,
fitnessSD,printTumor);

  int make = 0;
  while (make < numTumors) {
  // Initialize random number generator
    cout << "Seed set to " << seed+make << endl;
    mt19937_64 rng(seed+make);

    Tumor tumor(expectedMutations,fitnessMean,fitnessSD);

    while ( tumor.size() < tumorSize  && tumor.size() > 0 )
      {
        tumor.change(rng);
        if ( tumor.size() % 10000 == 0 ) {
          cerr << "tumor size = " << tumor.size() << endl;
        }
      }
    if (printTumor) {
      tumor.print(cout);
    }
    tumor.slice(); //Slice the tumor
    ostringstream filename;
    filename << "mutation_data_" << seed+make << ".csv";
    string fn = filename.str();
    ofstream Mutation_Data (fn);
    Mutation_Data << "seed,ID,slice_freq,tum_freq,tum_size,fit_change" << endl;
    vector<Mutation*> copyOfMutations = tumor.getMutations();
    //DATA PRINTING
    for (vector<Mutation*>::iterator m = copyOfMutations.begin(); m !=
copyOfMutations.end(); ++m) {
      Mutation_Data << seed+make << ","
                    << (*m)->getID() << ","
                    << double((*m)->getNumSliceCrypts())/tumor.getSliceSize() << ","
                    << double((*m)->getNumTumorCrypts())/tumor.size() << ","
                    << (*m)->getTumorSize() << ","
                    << (*m)->getFitnessChange() << endl;
```

```
    }
    make++;
  }

  return 0;
}
```
